# Structural Analysis of (p)ppGpp Reveals Its Versatile Binding Pattern for Diverse Types of Target Proteins

**DOI:** 10.3389/fmicb.2020.575041

**Published:** 2020-11-05

**Authors:** Gajraj Singh Kushwaha, Anupam Patra, Neel Sarovar Bhavesh

**Affiliations:** ^1^Transcription Regulation Group, International Centre for Genetic Engineering and Biotechnology (ICGEB), New Delhi, India; ^2^KIIT Technology Business Incubator (KIIT-TBI), Kalinga Institute of Industrial Technology (KIIT) (Deemed to be University), Bhubaneswar, India

**Keywords:** stringent response, (p)ppGpp, secondary messenger nucleotide, interaction analysis, structural dynamics, (p)ppGpp synthetase

## Abstract

(p)ppGpp, highly phosphorylated guanosine, are global regulatory nucleotides that modulate several biochemical events in bacterial physiology ranging from core central dogma to various metabolic pathways. Conventionally, (p)ppGpp collectively refers to two nucleotides, ppGpp, and pppGpp in the literature. Initially, (p)ppGpp has been discovered as a transcription regulatory molecule as it binds to RNA polymerase and regulates transcriptional gene regulation. During the past decade, several other target proteins of (p)ppGpp have been discovered and as of now, more than 30 proteins have been reported to be regulated by the binding of these two signaling nucleotides. The regulation of diverse biochemical activities by (p)ppGpp requires fine-tuned molecular interactions with various classes of proteins so that it can moderate varied functions. Here we report a structural dynamics of (p)ppGpp in the unbound state using well-defined computational tools and its interactions with target proteins to understand the differential regulation by (p)ppGpp at the molecular level. We carried out replica exchange molecular dynamics simulation studies to enhance sampling of conformations during (p)ppGpp simulation. The detailed comparative analysis of torsion angle conformation of ribose sugar of unbound (p)ppGpp and bound states of (p)ppGpp was carried out. The structural dynamics shows that two linear phosphate chains provide plasticity to (p)ppGpp nucleotides for the binding to diverse proteins. Moreover, the intermolecular interactions between (p)ppGpp and target proteins were characterized through various physicochemical parameters including, hydrogen bonds, van der Waal’s interactions, aromatic stacking, and side chains of interacting residues of proteins. Surprisingly, we observed that interactions of (p)ppGpp to target protein have a consensus binding pattern for a particular functional class of enzymes. For example, the binding of (p)ppGpp to RNA polymerase is significantly different from the binding of (p)ppGpp to the proteins involved in the ribosome biogenesis pathway. Whereas, (p)ppGpp binding to enzymes involved in nucleotide metabolism facilitates the functional regulation through oligomerization. Analysis of these datasets revealed that guanine base-specific contacts are key determinants to discriminate functional class of protein. Altogether, our studies provide significant information to understand the differential interaction pattern of (p)ppGpp to its target and this information may be useful to design antibacterial compounds based on (p)ppGpp analogs.

## Introduction

Bacterial physiology is regulated by various types of secondary messenger nucleotides and these nucleotides regulate almost all major biochemical events (Hengge et al., 2019). These nucleotides are key players of the signaling network intended to responsive cellular behavior to various environmental conditions. The stringent response is such a pleiotropic and global regulatory process which modulates at least one-third of bacterial physiological processes (Cashel and Gallant, 1969; Cashel et al., 1996; Hauryliuk et al., 2015). It is meticulously regulated by the synthesis of two signaling nucleotides namely, pppGpp and ppGpp (together called (p)ppGpp) (Potrykus and Cashel, 2008; Hauryliuk et al., 2015; Steinchen and Bange, 2016). (p)ppGpp messenger nucleotides are highly phosphorylated and bind to several protein targets to regulate biochemical events (Dalebroux and Swanson, 2012; Kanjee et al., 2012; Zhang et al., 2018). Historically, these nucleotides were discovered by two-dimensional thin-layer chromatography of samples from amino acid starved *Escherichia coli* culture. The concentration of these nucleotides was enhanced drastically in *E coli* cells during amino acid starvation and these nucleotides were inhibiting rRNA synthesis (Cashel and Gallant, 1969). Initially, the functional role of (p)ppGpp was discovered as transcriptional regulatory as it binds to bacterial RNA polymerase to down-regulate rRNA gene expression (Cashel and Gallant, 1969; Ross et al., 2013; Zuo et al., 2013). Later on, several enzymes from various pathways were identified which are regulated by binding of (p)ppGpp nucleotides. Most of those proteins are part of the core process of molecular machinery such as replication, transcription, translation, and cellular metabolism (Rojas et al., 1984; Wang et al., 2007, 2019; Srivatsan and Wang, 2008; Kriel et al., 2012; Bærentsen et al., 2019; Kushwaha et al., 2019b). The resultant effects of (p)ppGpp mediated regulation has been shown in virulence, host invasion, biofilm formation, persistence, long term survival, pathogenesis, antibiotic resistance, and antibiotic tolerance (Primm et al., 2000; Abranches et al., 2009; Dalebroux et al., 2010; Kudrin et al., 2017; Prusa et al., 2018). Therefore, being a modulator of several processes, (p)ppGpp has been considered as a master regulator for the survival of bacteria during unfavorable conditions.

Conventionally, term (p)ppGpp is used for two nucleotides, guanosine 5′-diphosphate-3′-diphosphate (ppGpp) and guanosine 5′-triphosphate-3′-diphosphate (pppGpp). These nucleotides are synthesized by (p)ppGpp synthetase by transferring pyrophosphate groups from ATP to GDP/GTP to form ppGpp and pppGpp, respectively (Mechold et al., 2002; Hogg et al., 2004; Tamman et al., 2020). There are primarily two types of (p)ppGpp synthetases that have been identified, multi-domain long RelA type and small alarmone synthetases. The long-form (p)ppGpp synthetases are found in two forms; mono functional comprises active synthetase and inactive hydrolase domain while bifunctional (p)ppGpp synthetases have both synthetase and hydrolase active domain. These are classified and named as RelA/SpoT Homolog (RSH) proteins (Atkinson et al., 2011; Hauryliuk et al., 2015). Several regulatory mechanisms have been proposed to explain the activation of (p)ppGpp synthetase enzymes (Hogg et al., 2004; Shyp et al., 2012; Steinchen et al., 2015; Beljantseva et al., 2017; Hauryliuk and Atkinson, 2017; Winther et al., 2018; Kushwaha et al., 2019a; Ronneau and Hallez, 2019). Subsequently, these nucleotide binds to various proteins to modulate the functional activity of respective biochemical reactions, hence, the structural information at the molecular level is essential to understand the fundamental differences associated with these biomolecular interactions. Although the structural studies on (p)ppGpp have been carried out in the bound form as complexes with its binding protein, the structural conformation and dynamics on (p)ppGpp in unbound states have not been reported so far. The computational methods have been an efficient choice to understand the structural dynamics of secondary messenger nucleotides (Stern et al., 2010; Wang et al., 2017). Here, we report a detailed structural analysis on (p)ppGpp in the unbound state using extensive conformation sampling molecular dynamics simulation along with their comparison with structural conformation in bound states. Additionally, a systematic analysis of the interactions between (p)ppGpp and binding proteins has been carried out to probe the particular binding pattern of these interactions for various types of proteins.

## Materials and Methods

### Replica Exchange Molecular Dynamics (REMD) Simulation on Unbound (p)ppGpp

The molecular dynamics simulation studies provide structural dynamic information in the solution state of a molecule. Although, glycosidic bond and ribose sugar in the nucleotide structure exhibit conformational flexibility and classical molecular dynamics simulation on nucleotides is challenging because of energy barriers and sampling of conformations are limited. Therefore, Replica Exchange Molecular Dynamics (REMD) simulation was carried out on ppGpp and pppGpp nucleotides in solution states. In REMD, several identical replicas run in parallel at different temperatures and these allow enhanced sampling of high energy conformations. Moreover, these replicas are allowed to swap their states based on Boltzmann-weighted probability at neighboring temperature state. This process is repeated iteratively during the simulation and subsequently enhanced sampling of conformations is achieved at various temperatures.

**FIGURE 1:**
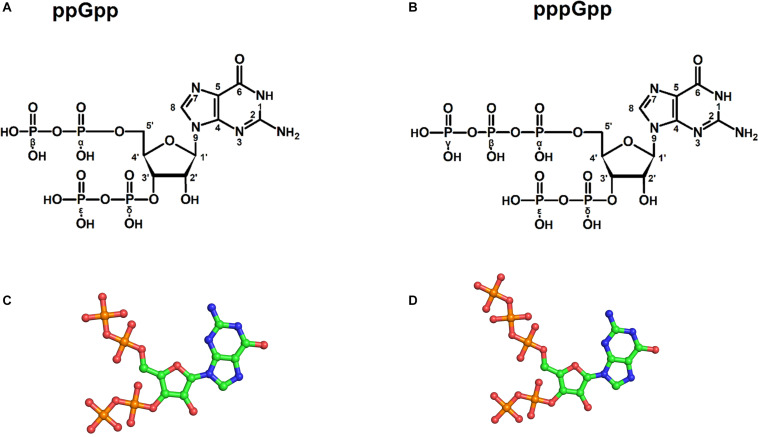
Structures of (p)ppGpp signaling nucleotides: a two-dimensional chemical sketch of ppGpp **(A)**, and pppGpp **(B)**. Ball and stick model of ppGpp **(C)**, and pppGpp **(D)**. The color coding for atoms in ball stick model is displayed as follows; green-carbon, blue-nitrogen, red-oxygen, and orange-phosphorous atoms.

The structural coordinates of ppGpp and pppGpp were extracted from Protein Data Bank (Berman et al., 2000) and were prepared for simulation using the Maestro program from Schrodinger suite (Figures 1A–D). LigPrep tool was used to retain original chirality and biological pH 7.0 ± 2.0 of the (p)ppGpp nucleotides. For each nucleotide, the lowest energy conformer was used for the simulation process. The simulation system was built using the system builder tool in Maestro. The explicit solvent model TIP3P (Jorgensen et al., 1983)was included in an orthorhombic periodic boundary condition (PBC) computational box. The initial absolute box volume was 1000 Å^3^ for both molecules and upon addition of buffer, the box volume was expanded to 145262 and 150766 Å^3^ for ppGpp and pppGpp, respectively. Negative charges, due to phosphates groups of (p)ppGpp molecule, were neutralized by the addition of 20 mM Mg^2+^ in the simulation system. The Optimized Potential for Liquid Simulations 3 enhanced (OPLS3e) force field was selected for the simulation (Roos et al., 2019). Next, the prepared systems of ppGpp and pppGpp were loaded to Desmond workspace for energy minimization and replica-exchange simulation. The system was energy minimized before running the simulations. The replica-exchange parameters were set in a replica-exchange panel in Desmond. A tempering method was selected with nine replicas covering of temperature range from 273 to 373 K. The simulation time was fixed as 200 ns with a recording interval of 200 ps trajectory with energy of 1.2. The ensemble was selected as NPT for replica exchange. Finally, both REMD simulations were carried out for 200 ns. The REMD simulation result statistics were analyzed using the Desmond simulation interaction diagram report. The molecular properties of simulation trajectory were plotted using root-mean-square fluctuation (RMSF), root-mean-square deviation (RMSD), radius of gyration (rGyr), intramolecular hydrogen bonds (intraHB), molecular surface area (MolSA), solvent-accessible surface area (SASA), and polar surface area (PSA) parameters.

### Structural Comparison of (p)ppGpp in Unbound and Bound State

The energy minimized three-dimensional structural coordinates of ppGpp and pppGpp were compared with coordinates extracted from crystal structures of (p)ppGpp-protein complexes. The structural alignment was carried out in PyMol using an atom alignment algorithm. The distribution frequency of glycosidic bonds in unbound states of ppGpp and pppGpp were plotted at 300 K simulation pose in Schrodinger. The torsion angle and phase angle values were calculated from Pseudo-Rotational Online Service and Interactive Tool (PROSIT) (Sun et al., 2004, 2005).

### Interactions Analysis of (p)ppGpp and Proteins

The structures of (p)ppGpp-protein complexes were downloaded from Protein Data Bank (PDB). The PDB ligand code G4P for ppGpp, 0o2, and C1Z for pppGpp was used as a search term to obtain structures of ppGpp-protein and pppGpp-protein complexes, respectively. As shown in Table 1, there were 26 structures of ppGpp-protein while eight structures of pppGpp-protein complexes were found in the database. Four crystal structures of ppGpp-riboswitch complex were also found. The protein structures in complex with ppGpp and pppGpp were uploaded to the Arpeggio server (Jubb et al., 2017). Arpeggio server, based on Python, extract the interaction data between atoms located within 5 Å radial cutoff. The interaction results of each structures were downloaded from the server and converted to tabular form. The columns containing interactions involving proximal, clashes, covalent bond, halogen bonds, hydrophobic, carbonyl were removed before calculations. The nine interaction parameters were included in the interaction calculations including, van der Waal’s clashes, van der Waal’s interactions, hydrogen bonds, weak hydrogen bonds, ionic, metal complex, aromatic, polar, and weak polar interactions. The stereochemical parameters for the definition of interatomic interactions in the Arpeggio program are employed from the CREDO database (Marcou and Rognan, 2007; Schreyer and Blundell, 2009). The hydrogen bonds were considered as the distance at 2.8 to 3.5 Å and the angle between 120 and 180° while weak hydrogen bonds were shorter than 2.8 Å and having an angle less than 120°(Marcou and Rognan, 2007). The van der Waal’s interactions are defined as interactions between two non-hydrogen bonding atoms which are present in their van der Waal’s radii of corresponding atoms. The distance parameters for van der Waal’s interactions and clashes in the Arpeggio program are taken from Open Babel. All these interactions for 33 complexes of (p)ppGpp-protein are provided in the table (Supplementary Table S1). The occurrence of these non-covalent interactions between (p)ppGpp and functional class of protein was plotted to identify the binding pattern of (p)ppGpp to respective class. For the sake of clarity, the two-dimensional interaction diagrams of each representative (p)ppGpp-protein complex were also plotted using the PoseView tool from the Protein Plus server (Stierand et al., 2006; Fährrolfes et al., 2017).

**TABLE 1:** List of available structures in complex with (p)ppGpp (ligand code G4P C1Z, and 0o2) in Protein Data Bank. The references are provided within the main text.

S.No	Macromolecule Name	Resolution (Å)	PDB ID
1	SAS1 (RelQ)	2.94	5DED
2	SAS2 (RelP)	2.78	6EX0
3	Rel*_*Tt*_*	2.75	6S2T
4	SPO0B-associated GTP-binding protein	2.60	1LNZ
5	GTP-binding protein TypA/BipA	3.31	4ZCM
6	GTPase RbgA	1.80	6G14
7	GTPase RbgA	1.65	6G15
8	GTP-binding protein	4.00	5A9Y
9	Amidophosphoribosyltransferase PurF	1.95	6CZF
10	Hypoxanthine phosphoribosyltransferase	2.10	6D9S
11	Xanthine phosphoribosyltransferase	1.80	6W1I
12	Guanylate kinase	1.65	4QRH
13	Nucleosidase PpnN	2.77	6GFM
14	Putative phosphoribosyltransferase	1.50	5VOG
15	RNA polymerase	2.70	1SMY
16	RNA polymerase	3.90	4JK1
17	RNA polymerase	4.20	4JKR
18	RNA polymerase	2.71	5TMC
19	RNA polymerase	4.20	4JK2
20	RNA polymerase	4.29	5VSW
21	RNA polymerase	3.58	6WRG
22	RNA polymerase	3.62	6WRD
23	Exopolyphosphatase	2.71	2J4R
24	Guanosine pentaphosphate phosphohydrolase	2.76	6PC1
25	Peptide chain release factor 3	3.00	3VR1
26	Acetyltransferase A	2.34	4HNX
27	Acetyltransferase A	2.81	4XPD
28	Acetyltransferase A	3.95	4Y49
29	PRPP riboswitch	3.10	6CK4
30	ppGpp Riboswitch	2.20	6DMC
31	ppGpp Riboswitch	2.65	6DMD
32	ppGpp Riboswitch	2.70	6DME
33	DNA primase	2.01	4EDV
34	DNA primase	2.00	4EDT
35	Lysine decarboxylase, inducible	2.00	3N75
36	Aldo-keto reductase family protein	3.62	6GTM
37	Stringent starvation protein A	2.80	5U51
38	RNA pyrophosphohydrolase	2.06	6VCL

## Results and Discussion

### Structural Dynamics of Unbound (p)ppGpp

Molecular dynamics simulation is one of the most commonly used approaches to understand structural dynamics of biomolecules in solution state (Karplus and Petsko, 1990; Karplus and McCammon, 2002; Hollingsworth and Dror, 2018). It has been an efficient choice for the characterization of structural dynamics of free nucleotides and nucleic acid (Stern et al., 2010; Kobayashi et al., 2013; Šponer et al., 2014; Wang et al., 2017; Mlýnský and Bussi, 2018; Cassone et al., 2019). However, classical MD simulations studies on small molecules such as free nucleotides are challenging due to the presence of high energy barriers of the glycosidic bond between nucleotide base and ribose sugar as well as conformational flexibility of ribose moiety (Wang et al., 2017; Wang and Berne, 2018; Yang et al., 2019). To overcome the sampling issue, the replica-exchange method has been explored previously on small biomolecules to enhance sampling to cover more conformation space (Smith et al., 2016; Wang and Berne, 2018). Recently, cyclic nucleotides and small nucleic acid have been characterized for structural dynamics using REMD enhanced sampling methods (Šponer et al., 2014; Wang et al., 2017; Mlýnský and Bussi, 2018).

**FIGURE 2:**
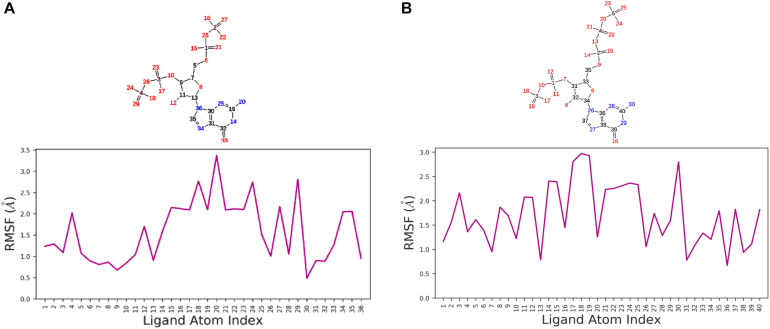
Replica exchange molecular dynamics simulation analysis. Atom numbering at top panel and root-mean-square fluctuation (RMSF) plot for ppGpp **(A)** and pppGpp **(B)** displays the fluctuation in individual atoms with reference to an initial state.

**FIGURE 3:**
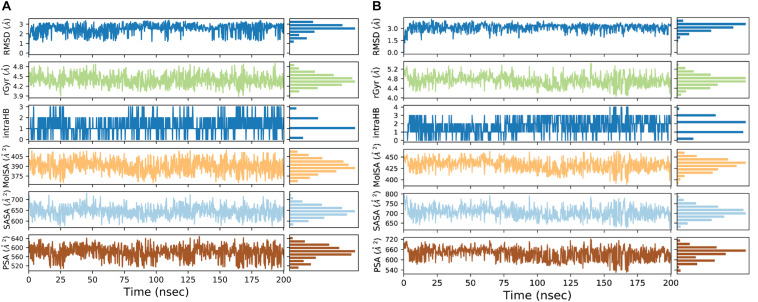
Molecular properties graph for the description of various parameters of ppGpp **(A)**, and pppGpp **(B)** during simulation run.

We used a well-defined protocol for REMD simulation to obtain the structural dynamics information of unbound ppGpp and pppGpp nucleotides in the solution state. A total of eight replicas simulation were exchanged at the 273, 285, 298, 310, 323, 335, 348, 360, 373 K during simulation covering temperature range from 273 to 373 K (Supplementary Figure S1). As shown in the graph, the RMSF, a parameter for displacement measurement of an atom in a molecular simulation trajectory in comparison with reference position, indicates that the major structural changes in (p)ppGpp molecules are observed (Figures 2A,B). There are 18 torsion bonds in pppGpp and 15 torsion bonds in ppGpp which can rotate therefore higher RMSF values are observed in these corresponding atoms. Although the negatively charged phosphate groups were neutralized by two Mg^2+^ ions during the simulation, yet higher flexibility of phosphate atoms was observed in the RMSF plot. In contrast to (p)ppGpp, the phosphate groups in cyclic messenger nucleotides show lesser flexibility due to the unavailability of free phosphate chains (Stern et al., 2010; Wang et al., 2017; Cassone et al., 2019). Based on our results, we propose that the dynamics in two free phosphate chains may provide greater flexibility to (p)ppGpp nucleotides for binding to diverse proteins. Surprisingly, the higher value of RMSF was observed in the NH_2_ group of guanine ring of (p)ppGpp in REMD simulation. The detailed analysis of molecular properties shows the overall quality and conformational dynamics of (p)ppGpp during the simulation (Figures 3A,B). These graphs show that molecular properties in ppGpp and pppGpp are similar, however, there are minor differences in these values that may be because of additional phosphate group present in pppGpp. The higher RMSD and rGyr show the conformational flexibility which indicates that (p)ppGpp nucleotides show dynamics in the unbound states which is necessary to accommodate in the various types of the binding site. The other important parameter of (p)ppGpp nucleotides is the SASA which depicts the surface area of molecules accessible by the water molecules. The higher SASA values of (p)ppGpp contribute water-mediated interactions to the proteins and it is consistent with structures of (p)ppGpp-protein-complexes. The molecular parameters including rGyr, intraHB, MolSA, SASA, and PSA exhibit slightly higher value for pppGpp as compare to ppGpp because of an additional γ phosphate group present in pppGpp. In addition to inter-molecular non-covalent interactions, water-mediated hydrogen bonds play a significant role in stabilizing small molecules in the binding site of the protein.

### Structural Comparison of (p)ppGpp in Unbound and Bound State

**FIGURE 4:**
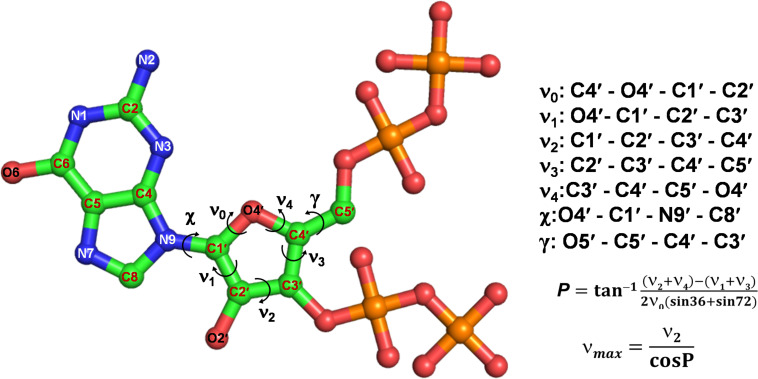
Different torsion angles in the structure of (p)ppGpp. The glycosidic torsion angle (χ) depicts the rotation between guanine and ribose ring, the angle γ denotes rotation between ribose ring and C4 branch of ribose sugar. The torsion angles of ribose rings are denoted fromν_0_ to ν_4_.

**FIGURE 5:**
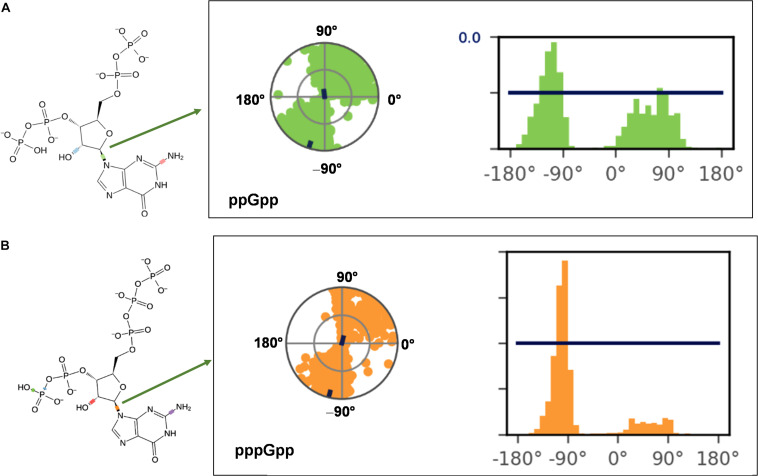
Probable frequency distribution of glycosidic torsion angle (χ) conformation in ppGpp **(A)** and pppGpp **(B)** during REMD simulation. The radial plot denotes the conformation of glycosidic bond throughout the simulation time course. The beginning of the simulation started at the center of a circle and radially outward portrays time evolution during the simulation run. The bar diagram representation of the distribution data of torsion angle. *X*-axis denotes the angle of glycosidic bond and *Y*-axis shows the number of times the bond was observed at this angle i.e., percentage frequency distribution. The value of χ falls into ranges of +90 to +180° (or 180 to 270°) corresponds to *anti* conformation while the value χ founds in the ranges of –90 to +90° refers to *syn* conformation of glycosidic bond.

The three-dimensional structures of unbound (p)ppGpp were obtained by energy minimization in Maestro. The structural coordinates of bound (p)ppGpp were extracted from structures of (p)ppGpp-protein complexes. The structural alignment of unbound state and bound state of (p)ppGpp shows substantial similarity in the guanine ring region with RMSD of atoms less than one. However, the phosphate chain shows divergence upon structural alignment which is in agreement with our simulation RMSF plot.

(p)ppGpp nucleotides exhibit a substantial extent of conformational flexibility because of various rotatable torsion angles present in the structure particularly a ribose ring (Figure 4). Therefore, we analyzed the glycosidic bond conformation of (p)ppGpp in unbound and bound states. The glycosidic conformation signifies the orientation of base and sugar in nucleotides and is measured as a torsion angle (χ) between O4’-C1’-N9-C4 of guanine. As shown in Figure 5, the distribution frequency of glycosidic bond conformation in both nucleotides observed during REMD was mostly in *syn* conformation, however, some occurrences were observed in *anti* conformation. Our results on the frequency distribution of glycosidic conformation in unbound states are similar to those observed in the bound states in the crystal structure. As most of the structures of (p)ppGpp-protein complexes have *anti* conformation of glycosidic bond except in few cases it was found in *syn* conformation such as nucleosidase (6GFM) (Zhang et al., 2019), lysine decarboxylase (3N75) (Kanjee et al., 2011). The sugar pucker in (p)ppGpp nucleotides in the bound state is observed majorly in *endo* conformation (Table 2). Whereas, the sugar pucker conformation energy minimized unbound states are found as *exo* for ppGpp and pppGpp (Table 2). The five endocyclic torsion angles, ν_0_, ν_1_, ν_2_, ν_3_, and ν_4_, of backbone atoms, define the conformation description of the ribose sugar (Figure 4). The ribose sugar of (p)ppGpp nucleotides shows substantial conformational flexibility in unbound and bound states (Table 2). Additional parameters to characterize ribose sugar conformation are pseudorotational phase angle (*P*) and maximum puckering amplitude (ν_max_) which show that ribose sugar adopts north conformation in both states (Sun et al., 2004). The comparative description of these torsion angles is given in the Table 2 for unbound states and few bound states of (p)ppGpp. All these parameters indicate that ribose moiety in (p)ppGpp behave dynamically in both unbound and bound states.

**TABLE 2:** Pseudorotational phase angle and puckering amplitude for (p)ppGpp nucleotides.

(p)ppGpp state	PDB	ν_0_	ν_1_	ν_2_	ν_3_	ν_4_	*P*	ν_max_	χ	γ	Sugar pucker
Unbound	ppGpp	−6.88	26.92	−35.87	33.24	−16.53	188.23	36.24	−99.58	−172.55	C3′-exo
Unbound	pppGpp	−7.88	26.79	−34.64	31.48	−14.82	186.23	34.85	84.22	−177.41	C3′-exo
(p)ppGpp synthetase	6EX0	−8.46	−13.71	31.52	−37.17	27.46	31.47	36.96	171.65	61.18	C3′-endo
RNA polymerase	5VSW	−13.05	25.53	−27.83	19.75	−4.28	170.36	28.23	−116.08	−177.02	C2′-endo
GTPase RbgA	6G14	−22.30	33.40	−32.51	18.75	2.27	158.59	34.92	−112.79	53.43	C2′-endo
Nucleosidase	6GFM	−32.38	44.35	−38.72	19.11	8.42	151.01	44.27	65.89	−143.14	C2′-endo

### Interactions Between (p)ppGpp and Proteins

**FIGURE 6:**
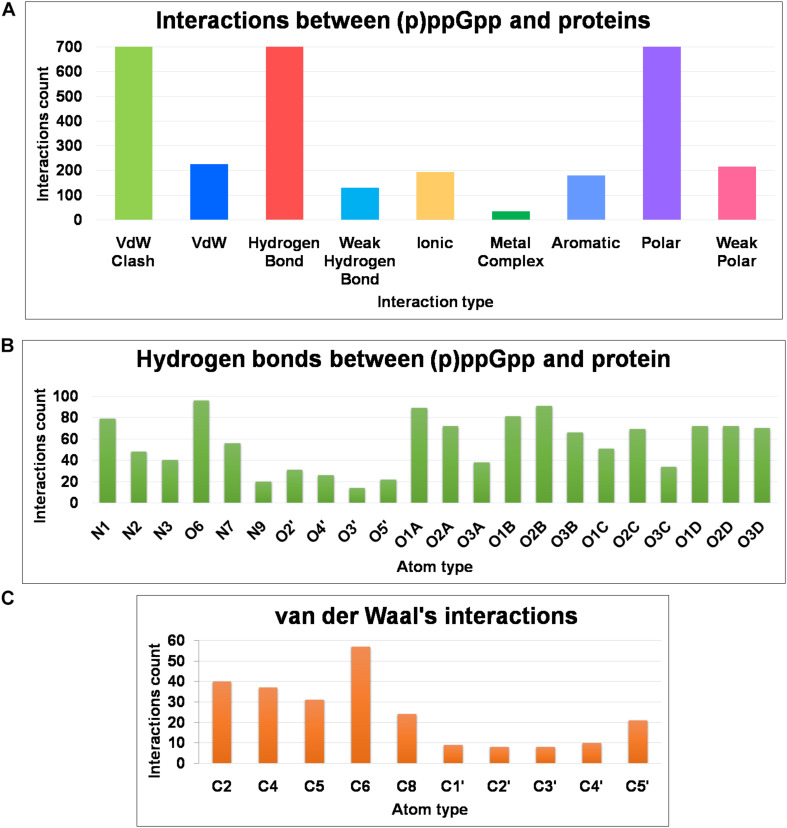
Frequency distribution of non-covalent interactions, between (p)ppGpp and protein, extracted from available crystal structures of (p)ppGpp-protein complexes. **(A)** Various types of inter-molecular interactions and their occurrences. **(B)** Atom-level distribution frequency of hydrogen bonds found in the (p)ppGpp-protein complexes. **(C)** Atom-level distribution frequency of van der Waal’s interactions found between (p)ppGpp and protein.

The structural diversity of secondary messenger nucleotides deliver generous adaptability for the binding to the cognate proteins. Based on their structural architecture, these nucleotides may be divided into two classes; linear and cyclic secondary nucleotides. The interaction analysis of cyclic secondary nucleotides with their binding protein complexes have been characterized extensively (Moodie and Thornton, 1993; Wang et al., 2017; Cassone et al., 2019; He et al., 2020). These studies provide significant information about the binding mode and interaction pattern of cyclic nucleotides and their protein complexes (He et al., 2020). Similarly, linear secondary messenger nucleotides, (p)ppGpp binds to a diverse class of proteins and should exhibit a particular binding mode for each functional class of protein. We have examined these interaction patterns using various computational tools to assess the binding stereochemistry between (p)ppGpp and respective protein. A detailed survey has been carried out on available structures of (p)ppGpp-protein complexes and the non-covalent interactions were quantified (Figure 6A). In addition to conventional interatomic interactions, various other types of molecular interaction fingerprints were included in the study which provides additional information for binding stability of ligand (Marcou and Rognan, 2007). Other types of non-conventional interactions that were included in the analysis are weak polar and weak hydrogen bonds that denote the hydrogen bonds without considering angles. A total of five major types of bonding parameters were included in the analysis interactions between (p)ppGpp and proteins. Among these interactions, hydrogen bonds contribute most as five nitrogen and 17 oxygen atoms in the (p)ppGpp have properties to make hydrogen bonds (Figure 6B). In the structures of many complexes, the phosphate group of (p)ppGpp nucleotide is observed as making interaction to protein through Mg^2+^ ions. As seen by the hydrogen bonds histogram, the major interatomic polar interactions were observed in guanine ring and phosphate group atoms. The aromatic ring of guanine hadπ-π base stacking interactions with the side chain of tyrosine in the structures of (p)ppGpp synthetases and nucleotide metabolic enzymes. The ribose moiety of (p)ppGpp makes comparatively fewer interactions with protein atoms which may be the consequence of the structural restrain of the ribose ring. Similarly, van der Waal’s interactions were found majorly in the region of the guanine ring of (p)ppGpp (Figure 6C). The available structures of the complexes between (p)ppGpp and protein span several functional classes. Here the characterization of (p)ppGpp-protein interactions focuses only on four major functional classes.

### (p)ppGpp Synthetase

**FIGURE 7:**
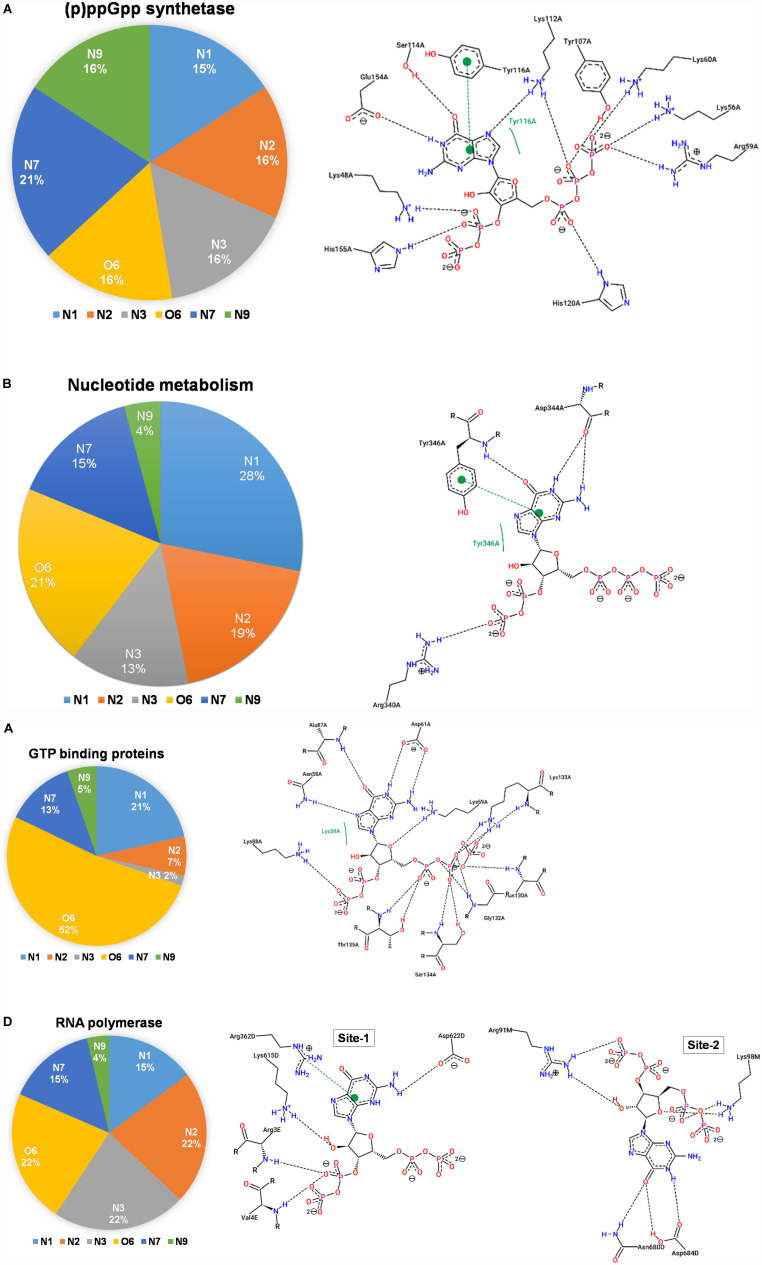
Interactions between (p)ppGpp atoms and protein are identified in the crystal structures of (p)ppGpp-protein complexes. The left panel of each figure displays the frequency distribution of interactions formed by guanine atoms of (p)ppGpp with protein atoms. The right panel of each figure indicates the two-dimensional interaction diagram between (p)ppGpp and protein. The interaction plots, generated by PoseView tool, show hydrogen bonds between (p)ppGpp and protein. **(A)** (p)ppGpp synthetase (5DED), **(B)** nucleotide metabolic enzymes (6GFM), **(C)** GTPase (6G15), and **(D)** RNA polymerase (5VSW).

The (p)ppGpp synthetases are apparent (p)ppGpp binding proteins as these enzymes synthesize ppGpp and pppGpp from ATP and GDP/GTP, respectively. However, most of the structures determined so far are in complexes with substrate analogs as these complexes explain the catalytic mechanism. A brief structural review on (p)ppGpp synthetase proteins has been described previously (Kushwaha et al., 2019b) hence current study focus only on (p)ppGpp and (p)ppGpp synthetase complexes. There are three structures of (p)ppGpp synthetases available in the Protein Data Bank in complex with (p)ppGpp. It includes two single domain (p)ppGpp synthetases, small alarmone synthetase-1 (SAS1) from *Bacillus subtilis* (Steinchen et al., 2015); small alarmone synthetase-2 (SAS2) from *Staphylococcus aureus* (Manav et al., 2018) and one long (p)ppGpp synthetase Rel*_*Tt*_* from *Thermus thermophiles* (Tamman et al., 2020). The (p)ppGpp binding site is primarily comprised of polar residues which hold the (p)ppGpp strongly by making several hydrogen bonds. The most distinguishing feature of these complexes is aromatic stacking with the guanine ring which is stacked by the side chain-ring of tyrosine. The negatively charged phosphate groups are stabilized by ionic interactions with the guanidinium group of several arginine residues. The Mg^2+^ ions also contribute to stabilizing these phosphate groups. As shown the Figure 7A, the nitrogen and oxygen atoms of the guanine ring significantly contribute to the hydrogen bond interactions with (p)ppGpp synthetase proteins. This type of binding pattern of (p)ppGpp may be considered as an optimum binding pattern for (p)ppGpp interactions with proteins.

### Nucleotide Metabolic Enzymes

There are six crystal structures of (p)ppGpp and nucleotide metabolic enzymes complexes available in the Protein Data Bank which includes Xanthine phosphoribosyltransferase (XPRT) (Anderson et al., 2020), Pyrimidine/purine nucleotide 5′-monophosphate nucleosidase (nucleosidase, PpnN) (Zhang et al., 2019), Hypoxanthine phosphoribosyltransferase (HPRT) (Anderson et al., 2019), Amidophosphoribosyltransferase (PurF) (Wang et al., 2019), Guanylate kinase (Liu et al., 2015; Table 1). A most striking feature of the structures of these enzyme-(p)ppGpp complexes are oligomeric stoichiometry in these complexes. The (p)ppGpp nucleotides facilitates the oligomeric cooperation through binding at an oligomeric interface. Similar to (p)ppGpp synthetases, (p)ppGpp binding site in these enzymes is primarily comprised of residues containing side chains with the polar groups for hydrogen bonding and aromatic ring for stacking interactions. The characteristic feature of (p)ppGpp binding to enzymes involved in nucleotide metabolism is aromatic stacking interaction between guanine base of (p)ppGpp and the aromatic ring-containing side chain of residues of corresponding binding proteins (Figure 7B). For example, Phe126 in XPRT, Trp153 in XGPRT, Tyr346 in PpnN, Phe152 in HPRT, Tyr83 in guanylate kinase makes stacking interactions. However, the aromatic stacking was not observed in the structure of PurF-ppGpp complex. The ribose ring generally interacted with protein though water-mediated hydrogen bonds interactions. The negatively charged phosphate groups of (p)ppGpp nucleotides are stabilized by mostly guanidine moiety of arginine side chains and Mg^2+^ ions. Therefore, these interactions are considered as ionic interactions. The observed binding mode of (p)ppGpp to the nucleotide enzymes is similar due to an analogy of substrate nucleotide structure to the (p)ppGpp. As shown in the graph, the N1, N2, N7, and O6 atoms of the aromatic guanine ring of (p)ppGpp is a major contributor to hydrogen bond interaction with protein residues.

### GTP Binding GTPase Proteins

There are five crystal structures of GTP binding GTPase proteins in complex with (p)ppGpp available till now. These include Obg GTP binding protein (Buglino et al., 2002), GTPase BipA/TypA (Fan et al., 2015), GTPase BipA (Kumar et al., 2015), and GTPase RbgA (Pausch et al., 2018). The (p)ppGpp binding site in GTPases proteins are shallower in comparison to (p)ppGpp synthetases and nucleotide metabolic enzymes. In contrast to (p)ppGpp synthetases, and nucleotide metabolic enzymes, aromatic stacking and guanidinium moiety was not observed in the structures of GTPases in complexes with (p)ppGpp. Interestingly, the major interaction of (p)ppGpp guanine ring was contributed by the O6 atom of (p)ppGpp (Figure 7C). The metal ions were also not observed to neutralize the negatively charged phosphate group of (p)ppGpp. Therefore, the major forces for the stabilization of (p)ppGpp are hydrogen bonds and van der Waal’s interactions.

### RNA Polymerase

RNA polymerase was the first protein complex reported to regulate its activity by binding of (p)ppGpp (Potrykus and Cashel, 2008). There are two (p)ppGpp binding sites that have been reported in the RNA polymerase which modulates allosteric transcription regulation (Molodtsov et al., 2018). Site 1 is located at the interface formed by β′ and ω subunits of RNA polymerase. This site is comparatively shallower and the guanine ring of (p)ppGpp interacts with the side chain of arginine, isoleucine, histidine, and aspartic acid residues. The second (p)ppGpp binding site is found at the secondary channel and it acts synergistically by the binding of a transcription regulator protein, DksA. Therefore, complete binding of (p)ppGpp to site 2 is accomplished by the interactions between (p)ppGpp and residues of β′ rim and DksA. In the site 2, the guanine ring of (p)ppGpp is stabilized by interactions between (p)ppGpp and side chains of aspartic acid, tyrosine, asparagine, and isoleucine while negatively charged phosphate group is neutralized by basic residues such as lysine and arginine (Figure 7D). The binding of (p)ppGpp to site 2, exhibit a allosteric change in the corresponding areas of RNAP and DksA to facilitate transcription regulation.

### Other (p)ppGpp Binding Proteins

There are several crystal structures of various other functional class of (p)ppGpp binding proteins reported including translation peptide chain release factor 3 (PDB: 3VR1) (Kihira et al., 2012), acetyltransferase A (PDB: 4HNX, 4XPD, 4Y49), PPX/GppA phosphatases (PDB: 2J4R, 6PC1) (Kristensen et al., 2008; Song et al., 2020), DNA primase (PDB: 4EDV, 4EDT) (Rymer et al., 2012), lysine decarboxylase (PDB: 3N75) (Kanjee et al., 2011), aldo-keto reductase (PDB: 6GTM)and RNA pyrophosphohydrolase (PDB: 6VCL) (Gao et al., 2020). The PPX/GppA phosphatases are pppGpp hydrolyzing enzymes that remove the γ-phosphate group from pppGpp to make ppGpp nucleotide, therefore, pppGpp serves as a substrate for these enzymes. DnaG is a DNA dependent RNA polymerase primase, responsible for primer synthesis during DNA replication. DnaG primase binds to various nucleotides including (p)ppGpp nucleotides. Lysine decarboxylase is an acid response protein that catalyzes the decarboxylation of L-lysine. (p)ppGpp binds to Ldcl and inhibits its enzymatic activity. RppH is Nudix hydrolase enzyme involved in RNA processing. It hydrolyzes various nucleotides including (p)ppGpp alarmone. The detailed interactions analysis and binding pattern of these (p)ppGpp-complexes are given in Supplementary Figure S2.

### Role of Magnesium Ion in (p)ppGpp and Protein Interactions

Magnesium ion (Mg^2+^) plays a significant role in the structural stability of nucleic acid and nucleotides by neutralizing highly negative charged phosphate groups (Black et al., 1994; Pechlaner and Sigel, 2012; Leonarski et al., 2017). Interestingly, highly phosphorylated (p)ppGpp has two linear chains of phosphate groups therefore Mg^2+^ ions assist in the specific interaction of (p)ppGpp to their target protein. As shown in the graph (Figure 6), we have observed several metal interactions in the structures of (p)ppGpp-protein complexes. In (p)ppGpp synthetases structures, Mg^2+^ ions provide robust physical support to the flexible phosphate chains. In the case of SAS1, one Mg^2+^ ion firmly stabilizes two phosphate chains with the side chain of Lys 32 while in the case of long-form of (p)ppGpp synthetase, Rel*_*Tt*_*, it binds to only one chain of phosphate (Tamman et al., 2020). In addition to physical stability, Mg^2+^ ions assist in the deprotonation of 3′ OH of GDP/GTP by acidic residues in Rel*_*Seq*_* and SAS1 (Hogg et al., 2004; Steinchen et al., 2015). Similarly, in the case of nucleotide metabolic enzymes, Mg^2+^ ions provide not only physical stability to one chain of (p)ppGpp but it also mediates interactions between (p)ppGpp and protein side chains. Each phosphate chain of (p)ppGpp is making interactions with one Mg^2+^ ion in the complex of RNA polymerase-(p)ppGpp. Altogether, we have observed Mg^2+^ ions in several other complexes of (p)ppGpp-protein structures except for few complexes which may be the limitation of electron density interpretation as discussed earlier (Leonarski et al., 2017).

## Conclusion

Secondary messenger nucleotides are key signaling molecules that modulate several cellular functions particularly in response to environmental changes. These nucleotides bind to several enzymes involve in various functional activities. The versatility of the binding mode of secondary nucleotides is facilitated by the conformation flexibility of glycosidic bond, ribose sugar puckering. In contrast to the cyclic nucleotides, (p)ppGpp have linear phosphate chains that provide additional flexibility to adapt various conformations according to the stereochemistry of the binding site of a respective target protein. Overall, our results support the hypothesis that the conformation flexibility of glycosidic bond, ribose sugar puckering, and phosphate groups provide structural plasticity to the (p)ppGpp for binding to the various functional class of proteins. The structures of unbound ppGpp and pppGpp in solution states obtained by MD simulation are similar to that observed in the structures in bound form, hence, these structures resemble biologically active conformations of (p)ppGpp. The gyration conformation profile of glycosidic bond is in agreement with the conformation of (p)ppGpp observed in the bound state. The analysis of (p)ppGpp-protein interactions reveals that the binding pattern of these nucleotides governs the regulation for a particular class of target proteins.

## Data Availability Statement

The original contributions presented in the study are included in the article/Supplementary Material, further inquiries can be directed to the corresponding author/s.

## Author Contributions

GK and NB conceptualized the study. GK and AP performed the study. All authors analyzed the data, wrote and edited the manuscript, and agreed on the final version of the manuscript.

## Conflict of Interest

The authors declare that the research was conducted in the absence of any commercial or financial relationships that could be construed as a potential conflict of interest.
