# Supplementary material for: Structural Analysis of (p)ppGpp Reveals Its Versatile Binding Pattern for Diverse Types of Target Proteins
Source: Front Microbiol. 2020 Nov 5;11:575041. doi: 10.3389/fmicb.2020.575041 (PMC7674647; doi:10.3389/fmicb.2020.575041)
Supplement: Supplementary file 1 [file Data_Sheet_1.PDF]

PDB	Atom 1	Atom 2	VdW Clash	VdW	Hydrogen Bond	Weak Hydrogen Bond	Ionic	Metal Complex	Aromatic	Polar	Weak Polar	Interacting entities
1NLZ	B/902/MG	B/600/C5'	0	0	0	0	0	0	0	0	0	INTER
	B/600/O2B	B/169/N	0	0	1	0	0	0	0	1	0	INTER
	B/600/O1B	B/168/CA	1	0	0	0	0	0	0	0	1	INTER
	B/312/N	B/600/O6	0	0	1	0	0	0	0	0	0	INTER
	B/311/N	B/600/O6	0	1	1	0	0	0	0	1	0	INTER
	B/311/N	B/600/C6	0	0	0	0	0	0	0	0	0	INTER
	B/310/C	B/600/O6	0	0	0	0	0	0	0	0	0	INTER
	B/282/OD1	B/600/O6	1	0	0	0	0	0	0	0	0	INTER
	B/310/CA	B/600/O6	0	1	0	0	0	0	0	0	1	INTER
	B/600/O1A	B/172/CB	0	0	0	1	0	0	0	0	1	INTER
	B/600/O1A	B/172/N	0	0	0	0	0	0	0	1	0	INTER
	B/170/C	B/600/O3A	0	1	0	0	0	0	0	0	0	INTER
	B/170/CA	B/600/O3A	1	0	0	0	0	0	0	0	1	INTER
	B/170/CA	B/600/O5'	0	0	0	1	0	0	0	0	0	INTER
	B/600/O3B	B/171/CB	0	0	0	1	0	0	0	0	1	INTER
	B/600/O2B	B/171/CB	0	1	0	1	0	0	0	0	1	INTER
	B/600/O2B	B/171/CE	1	0	0	0	0	0	0	0	1	INTER
	B/600/O2B	B/171/N	1	0	1	0	0	0	0	1	0	INTER
	B/600/O3A	B/171/N	0	0	0	0	0	0	0	1	0	INTER
	B/600/O2B	B/170/N	0	0	1	0	0	0	0	1	0	INTER
	B/600/O3A	B/170/N	1	0	1	0	0	0	0	1	0	INTER
	B/600/O3B	B/172/OG	1	0	0	0	0	0	0	1	0	INTER
	B/600/O3B	B/172/CA	0	0	0	0	0	0	0	0	0	INTER
	B/600/O3B	B/172/CB	0	0	0	0	0	0	0	0	1	INTER
	B/600/O3B	B/172/N	1	0	1	0	0	0	0	1	0	INTER
	B/600/O3B	B/901/MG	0	0	0	0	0	1	0	0	0	INTER
	B/902/MG	B/600/O2A	0	0	0	0	1	0	0	0	0	INTER
	B/902/MG	B/600/O1B	0	0	0	0	0	1	0	0	0	INTER
	B/168/N	B/600/O2B	0	0	1	0	0	0	0	1	0	INTER
	B/168/N	B/600/O1B	1	0	1	0	0	0	0	1	0	INTER
	B/600/O1A	B/170/CA	0	0	0	1	0	0	0	0	0	INTER
	B/173/OG1	B/600/O1A	1	0	0	0	0	0	0	1	0	INTER

	B/600/C8	B/173/CB	0	0	0	0	0	0	0	0	0	INTER
	B/600/C8	B/173/CG2	0	0	0	0	0	0	0	0	0	INTER
	B/600/O1A	B/172/O	0	0	0	0	0	0	0	0	0	INTER
	B/600/O1A	B/172/C	0	0	0	0	0	0	0	0	0	INTER
	B/600/O1A	B/173/C	0	0	0	0	0	0	0	0	0	INTER
	B/600/O1A	B/173/N	1	0	1	0	0	0	0	1	0	INTER
	B/600/O1A	B/173/CA	0	0	0	0	0	0	0	0	1	INTER
	B/600/O1A	B/173/CB	0	1	0	0	0	0	0	0	1	INTER
	B/600/C8	B/173/OG1	0	0	0	1	0	0	0	0	0	INTER
	B/600/N7	B/311/CB	0	0	0	1	0	0	0	0	0	INTER
	B/600/N7	B/282/ND2	1	0	1	0	0	0	0	1	0	INTER
	B/918/O	B/600/O2'	1	0	1	0	0	0	0	1	0	SELECTION_WATER
	B/600/C5'	B/168/O	0	0	0	1	0	0	0	0	0	INTER
	B/600/C5'	B/168/OG	0	1	0	0	0	0	0	0	1	INTER
	B/600/O4'	B/283/NZ	0	0	1	0	0	0	0	1	0	INTER
	B/1029/O	B/600/O1C	1	0	1	0	0	0	0	1	0	SELECTION_WATER
	B/1029/O	B/600/PC	0	1	0	0	0	0	0	0	0	SELECTION_WATER
	B/1029/O	B/600/O2C	0	0	0	0	0	0	0	1	0	SELECTION_WATER
	B/943/O	B/600/O2C	1	0	1	0	0	0	0	1	0	SELECTION_WATER
	B/283/CE	B/600/N9	0	0	0	1	0	0	0	0	0	INTER
	B/600/O6	B/310/OG	1	0	1	0	0	0	0	1	0	INTER
	B/600/O6	B/282/C	0	0	0	0	0	0	0	0	0	INTER
	B/600/O6	B/283/N	0	0	1	0	0	0	0	1	0	INTER
	B/600/O6	B/283/CA	0	0	0	1	0	0	0	0	1	INTER
	B/600/N1	B/283/CA	0	0	0	1	0	0	0	0	0	INTER
	B/600/N1	B/285/OD2	1	0	0	0	0	0	0	1	0	INTER
	B/285/OD1	B/600/N1	0	0	0	0	0	0	0	1	0	INTER
	B/285/OD1	B/600/N2	1	0	1	0	0	0	0	1	0	INTER
4ZCM	A/1004/MG	A/1003/O3D	0	0	0	0	1	0	0	0	0	INTER
	A/1004/MG	A/1003/O1D	0	0	0	0	1	0	0	0	0	INTER
	A/1003/O2B	A/16/N	1	0	1	0	0	0	0	1	0	INTER
	A/1003/O1A	A/16/N	1	0	0	0	0	0	0	1	0	INTER
	A/1003/O1A	A/15/C	1	0	0	0	0	0	0	0	0	INTER

	A/1003/O1B	A/18/N	0	1	1	0	0	0	0	1	0	INTER
	A/1003/O2B	A/17/N	0	0	1	0	0	0	0	0	0	INTER
	A/1003/O2A	A/17/CA	0	0	0	1	0	0	0	0	0	INTER
	A/1003/O1A	A/17/CA	1	0	0	0	0	0	0	0	1	INTER
	A/1003/O1A	A/17/N	1	0	1	0	0	0	0	1	0	INTER
	A/1003/O1B	A/19/N	1	0	1	0	0	0	0	1	0	INTER
	A/1003/O1B	A/19/CB	0	0	0	0	0	0	0	0	1	INTER
	A/1003/O2A	A/20/N	0	0	1	0	0	0	0	1	0	INTER
	A/1003/O2A	A/20/OG1	0	1	0	0	0	0	0	1	0	INTER
	A/1004/MG	A/1003/O3B	0	0	0	0	1	0	0	0	0	INTER
	A/15/N	A/1003/O2B	1	0	1	0	0	0	0	1	0	INTER
	A/15/CA	A/1003/O2B	1	0	0	0	0	0	0	0	1	INTER
	A/1003/O1B	A/18/CG	0	0	0	1	0	0	0	0	1	INTER
	A/15/CA	A/1003/O1A	1	0	0	0	0	0	0	0	1	INTER
	A/1003/C5	A/129/CG	0	1	0	0	0	0	0	0	0	INTER
	A/1003/N9	A/129/CE	0	0	0	0	0	0	0	0	1	INTER
	A/1003/C6	A/129/CG	1	0	0	0	0	0	0	0	0	INTER
	A/1003/C4	A/129/CE	0	1	0	0	0	0	0	0	0	INTER
	A/1003/N3	A/129/CG	0	0	0	1	0	0	0	0	0	INTER
	A/168/N	A/1003/O6	0	1	1	0	0	0	0	1	0	INTER
	A/128/OD1	A/1003/O6	1	0	0	0	0	0	0	0	0	INTER
	A/1003/N1	A/131/OD1	0	0	0	0	0	0	0	1	0	INTER
	A/1003/N1	A/129/CG	0	0	0	1	0	0	0	0	0	INTER
	A/1003/N1	A/129/CA	0	0	0	1	0	0	0	0	0	INTER
	A/1003/O6	A/167/N	1	0	1	0	0	0	0	1	0	INTER
	A/1003/O6	A/166/C	0	1	0	0	0	0	0	0	0	INTER
	A/1003/O6	A/166/CA	0	1	0	0	0	0	0	0	1	INTER
	A/168/CB	A/1003/C6	1	0	0	0	0	0	0	0	0	INTER
5A9Y	A/1603/N2	A/131/OD1	0	0	1	0	0	0	0	1	0	INTER
	A/1603/N1	A/131/OD1	1	0	0	0	0	0	0	1	0	INTER
	A/1603/C5	A/167/CB	1	0	0	0	0	0	0	0	0	INTER
	A/1603/PA	A/20/OG1	0	1	0	0	0	0	0	0	0	INTER
	A/1603/O2A	A/20/OG1	1	0	1	0	0	0	0	1	0	INTER

A/1603/PA	A/17/CA	0	1	0	0	0	0	0	0	0	INTER
A/1603/O2A	A/17/CA	0	0	0	1	0	0	0	0	0	INTER
A/1603/O1A	A/17/CA	1	0	0	0	0	0	0	0	1	INTER
A/1603/O1A	A/18/N	1	0	1	0	0	0	0	1	0	INTER
A/1603/C4'	A/16/O	0	0	0	0	0	0	0	0	1	INTER
A/1603/C5'	A/16/O	0	1	0	0	0	0	0	0	1	INTER
A/1603/O5'	A/16/O	1	0	0	0	0	0	0	0	0	INTER
A/1603/PA	A/16/O	1	0	0	0	0	0	0	0	0	INTER
A/1603/O2A	A/16/O	1	0	0	0	0	0	0	0	0	INTER
A/1603/O5'	A/16/C	1	0	0	0	0	0	0	0	0	INTER
A/1603/PA	A/16/C	1	0	0	0	0	0	0	0	0	INTER
A/1603/O1A	A/16/CA	1	0	0	0	0	0	0	0	1	INTER
A/1603/O1A	A/16/O	1	0	0	0	0	0	0	0	0	INTER
A/1603/O1A	A/16/C	1	0	0	0	0	0	0	0	0	INTER
A/1603/O1A	A/17/N	1	0	0	0	0	0	0	1	0	INTER
A/16/O	A/1603/O4'	1	0	0	0	0	0	0	0	0	INTER
A/1603/O5'	A/16/ND1	1	0	1	0	0	0	0	1	0	INTER
A/1603/O5'	A/16/CB	1	0	0	0	0	0	0	0	1	INTER
A/1603/O5'	A/16/CG	1	0	0	0	0	0	0	0	0	INTER
A/1603/O3A	A/16/ND1	0	0	0	0	0	0	0	1	0	INTER
A/1603/O3A	A/16/CE1	1	0	0	0	0	0	0	0	0	INTER
A/1603/PA	A/16/ND1	0	1	0	0	0	0	0	0	0	INTER
A/1603/PA	A/16/CG	0	1	0	0	0	0	0	0	0	INTER
A/2004/O	A/1603/O2A	1	0	1	0	0	0	0	1	0	SELECTION_WATER
A/1603/O1A	A/16/ND1	0	0	0	0	1	0	0	1	0	INTER
A/1603/O1A	A/16/CB	0	0	0	0	0	0	0	0	1	INTER
A/1603/O1A	A/16/CE1	0	0	0	0	1	0	0	0	0	INTER
A/1603/O1A	A/16/CG	1	0	0	0	1	0	0	0	0	INTER
A/1603/O1A	A/16/NE2	0	0	0	0	1	0	0	0	0	INTER
A/1603/O1B	A/16/ND1	0	0	0	0	1	0	0	0	0	INTER
A/1603/O1B	A/16/CE1	1	0	0	0	1	0	0	0	0	INTER
A/1603/O1B	A/16/CG	0	0	0	0	1	0	0	0	0	INTER
A/1603/O1B	A/16/NE2	1	0	0	0	1	0	0	1	0	INTER

	A/1603/O1A	A/16/CD2	1	0	0	0	1	0	0	0	0	INTER
	A/1603/O1B	A/16/CD2	0	0	0	0	1	0	0	0	0	INTER
	A/1603/N7	A/129/CE	0	0	0	1	0	0	0	0	0	INTER
	A/167/CB	A/1603/C6	1	0	0	0	0	0	0	0	0	INTER
	A/167/CB	A/1603/O6	1	0	0	0	0	0	0	0	1	INTER
	A/167/N	A/1603/O6	1	0	1	0	0	0	0	1	0	INTER
	A/128/OD1	A/1603/O6	1	0	0	0	0	0	0	0	0	INTER
	A/20/OG1	A/1603/O3B	1	0	0	0	0	0	0	1	0	INTER
	A/20/N	A/1603/O3B	0	0	0	0	0	0	0	1	0	INTER
	A/20/CB	A/1603/O3B	0	1	0	0	0	0	0	0	1	INTER
	A/19/C	A/1603/O3B	1	0	0	0	0	0	0	0	0	INTER
	A/167/CB	A/1603/N7	0	1	0	0	0	0	0	0	1	INTER
	A/19/N	A/1603/O3B	1	0	1	0	0	0	0	1	0	INTER
	A/18/N	A/1603/O3B	0	0	1	0	0	0	0	1	0	INTER
6G14	A/301/O5'	A/135/OG1	0	0	0	0	0	0	0	0	0	INTER
	A/301/C2'	A/135/OG1	0	0	0	1	0	0	0	0	0	INTER
	A/301/O2'	A/88/CD	0	0	0	1	0	0	0	0	0	INTER
	A/61/OD2	A/301/N1	0	0	1	0	0	0	0	0	0	INTER
	A/301/O5'	A/132/CA	0	0	0	1	0	0	0	0	0	INTER
	A/301/O2A	A/134/OG	0	0	0	0	0	0	0	1	0	INTER
	A/301/O2B	A/133/N	1	0	1	0	0	0	0	1	0	INTER
	A/301/O2B	A/133/CG	0	0	0	0	0	0	0	0	1	INTER
	A/301/O2B	A/133/CB	0	0	0	0	0	0	0	0	1	INTER
	A/449/O	A/301/O1B	1	0	1	0	0	0	0	1	0	SELECTION_WATER
	A/301/O3A	A/132/CA	0	1	0	0	0	0	0	0	1	INTER
	A/301/PA	A/132/CA	0	1	0	0	0	0	0	0	0	INTER
	A/301/O2A	A/401/O	1	0	1	0	0	0	0	1	0	SELECTION_WATER
	A/301/O2A	A/462/O	1	0	1	0	0	0	0	1	0	SELECTION_WATER
	A/130/O	A/301/C5'	0	0	0	1	0	0	0	0	0	INTER
	A/59/NZ	A/301/O4'	0	0	0	0	0	0	0	1	0	INTER
	A/59/CE	A/301/O4'	0	0	0	0	0	0	0	0	1	INTER
	A/59/CE	A/301/N9	0	0	0	1	0	0	0	0	0	INTER
	A/301/O3D	A/88/NZ	0	1	1	0	1	0	0	1	0	INTER

A/440/O	A/301/O1D	1	0	1	0	0	0	0	1	0	SELECTION_WATER
A/440/O	A/301/PD	1	0	0	0	0	0	0	0	0	SELECTION_WATER
A/301/O1C	A/407/O	1	0	1	0	0	0	0	1	0	SELECTION_WATER
A/301/O1C	A/478/O	1	0	1	0	0	0	0	1	0	SELECTION_WATER
A/301/O1D	A/407/O	0	0	1	0	0	0	0	1	0	SELECTION_WATER
A/301/PC	A/478/O	0	1	0	0	0	0	0	0	0	SELECTION_WATER
A/301/O3'	A/478/O	1	0	1	0	0	0	0	1	0	SELECTION_WATER
A/301/O2D	A/440/O	1	0	1	0	0	0	0	1	0	SELECTION_WATER
A/301/O5'	A/401/O	0	0	0	0	0	0	0	0	0	SELECTION_WATER
A/61/OD2	A/301/N2	1	0	1	0	0	0	0	1	0	INTER
A/301/O4'	A/487/O	0	0	0	0	0	0	0	1	0	SELECTION_WATER
A/301/N2	A/557/O	0	0	1	0	0	0	0	1	0	SELECTION_WATER
A/301/O3B	A/130/CA	0	0	0	0	0	0	0	0	1	INTER
A/301/O3A	A/130/CA	0	0	0	1	0	0	0	0	1	INTER
A/301/O3A	A/132/N	1	0	1	0	0	0	0	1	0	INTER
A/301/O2B	A/131/N	0	0	0	0	0	0	0	1	0	INTER
A/301/O2B	A/132/N	0	1	1	0	0	0	0	1	0	INTER
A/301/O3B	A/130/N	1	0	1	0	0	0	0	1	0	INTER
A/301/O3B	A/419/O	0	1	1	0	0	0	0	1	0	SELECTION_WATER
A/436/O	A/301/O3B	1	0	1	0	0	0	0	1	0	SELECTION_WATER
A/301/O2A	A/419/O	0	0	0	0	0	0	0	1	0	SELECTION_WATER
A/133/NZ	A/301/O3B	0	0	0	0	1	0	0	0	0	INTER
A/301/O2B	A/133/NZ	1	0	1	0	1	0	0	1	0	INTER
A/449/O	A/301/O2A	1	0	1	0	0	0	0	1	0	SELECTION_WATER
A/301/O1A	A/135/OG1	1	0	0	0	0	0	0	1	0	INTER
A/301/O1A	A/135/CB	0	0	0	0	0	0	0	0	1	INTER
A/58/ND2	A/301/N7	1	0	1	0	0	0	0	1	0	INTER
A/59/CA	A/301/N1	0	0	0	1	0	0	0	0	0	INTER
A/59/CA	A/301/O6	0	1	0	1	0	0	0	0	1	INTER
A/59/N	A/301/O6	0	0	1	0	0	0	0	1	0	INTER
A/88/N	A/301/O6	0	0	1	0	0	0	0	1	0	INTER
A/87/N	A/301/N1	0	0	0	0	0	0	0	0	0	INTER
A/87/N	A/301/O6	1	0	1	0	0	0	0	1	0	INTER

	A/86/OD1	A/301/N1	0	0	0	0	0	0	0	1	0	INTER
	A/86/OD1	A/301/O6	1	0	0	0	0	0	0	0	0	INTER
	A/86/CA	A/301/O6	0	0	0	0	0	0	0	0	1	INTER
	A/86/C	A/301/O6	0	0	0	0	0	0	0	0	0	INTER
	A/61/OD1	A/301/N1	1	0	1	0	0	0	0	1	0	INTER
	A/301/O1A	A/132/CA	0	1	0	1	0	0	0	0	1	INTER
	A/134/N	A/301/O1B	1	0	1	0	0	0	0	1	0	INTER
	A/134/N	A/301/O1A	0	0	0	0	0	0	0	1	0	INTER
	A/135/N	A/301/O1A	1	0	1	0	0	0	0	1	0	INTER
	A/432/O	A/301/O1B	1	0	1	0	0	0	0	1	0	SELECTION_WATER
	A/134/OG	A/301/O1B	1	0	1	0	0	0	0	1	0	INTER
	A/134/OG	A/301/O1A	1	0	1	0	0	0	0	1	0	INTER
	A/134/CB	A/301/O1B	0	0	0	0	0	0	0	0	1	INTER
6W1I	A/203/O1D	B/80/CZ	0	0	0	0	1	0	0	0	0	INTER
	A/203/O1D	B/80/NH1	1	0	1	0	1	0	0	1	0	INTER
	A/203/O1D	B/80/NH2	0	0	0	0	1	0	0	0	0	INTER
	A/203/O1D	A/316/O	1	0	1	0	0	0	0	1	0	SELECTION_WATER
	A/203/O3C	A/316/O	0	0	1	0	0	0	0	1	0	SELECTION_WATER
	A/203/O3C	A/382/O	0	0	1	0	0	0	0	1	0	SELECTION_WATER
	A/321/O	A/203/C5'	0	0	0	1	0	0	0	0	0	SELECTION_WATER
	A/203/O3D	B/80/CZ	0	0	0	0	1	0	0	0	0	INTER
	A/203/O3D	B/80/NH1	0	0	0	0	1	0	0	0	0	INTER
	A/203/O3D	B/80/NH2	1	0	1	0	1	0	0	1	0	INTER
	A/203/O3D	A/382/O	1	0	1	0	0	0	0	1	0	SELECTION_WATER
	A/201/NA	A/203/C1'	1	0	0	0	0	0	0	0	0	INTER
	A/203/O2'	A/429/O	0	0	1	0	0	0	0	1	0	SELECTION_WATER
	A/126/CE2	A/203/N3	0	0	0	0	0	0	1	0	0	INTER
	A/126/CE2	A/203/C2	0	0	0	0	0	0	1	0	0	INTER
	A/126/CE2	A/203/N1	0	0	0	0	0	0	1	0	0	INTER
	A/126/CD2	A/203/N3	0	0	0	0	0	0	1	0	0	INTER
	A/126/CD2	A/203/C2	0	0	0	0	0	0	1	0	0	INTER
	A/126/CZ	A/203/C2	0	0	0	0	0	0	1	0	0	INTER
	A/126/CZ	A/203/N1	0	0	0	0	0	0	1	0	1	INTER

A/126/CE1	A/203/N1	0	0	0	0	0	0	1	0	0	INTER
A/400/O	A/203/O5'	0	0	1	0	0	0	0	1	0	SELECTION_WATER
A/203/O1B	A/129/ND2	0	1	1	0	0	0	0	1	0	INTER
A/126/CZ	A/203/C6	0	0	0	0	0	0	1	0	0	INTER
A/126/CE1	A/203/C6	0	0	0	0	0	0	1	0	0	INTER
A/126/CD1	A/203/C6	0	0	0	0	0	0	1	0	0	INTER
A/126/CD1	A/203/C5	0	0	0	0	0	0	1	0	0	INTER
A/203/O4'	A/389/O	0	1	1	0	0	0	0	1	0	SELECTION_WATER
A/203/N3	B/85/CD2	0	0	0	0	0	0	0	0	1	INTER
A/203/C4	B/85/CD2	0	1	0	0	0	0	0	0	0	INTER
A/203/N3	A/429/O	1	0	1	0	0	0	0	1	0	SELECTION_WATER
A/203/C1'	A/429/O	0	0	0	1	0	0	0	0	0	SELECTION_WATER
A/331/O	A/203/N2	1	0	1	0	0	0	0	1	0	SELECTION_WATER
A/20/O	A/203/N2	0	1	1	0	0	0	0	1	0	INTER
A/20/O	A/203/N1	1	0	0	0	0	0	0	1	0	INTER
A/203/N2	B/350/O	0	0	1	0	0	0	0	1	0	SELECTION_WATER
A/203/N2	A/27/OD1	0	0	1	0	0	0	0	1	0	INTER
A/203/C8	A/389/O	0	0	0	0	0	0	0	0	1	SELECTION_WATER
A/312/O	A/203/O3B	1	0	1	0	0	0	0	1	0	SELECTION_WATER
A/433/O	A/203/O2A	0	1	1	0	0	0	0	1	0	SELECTION_WATER
A/100/OG1	A/203/O3B	0	0	0	0	0	0	0	1	0	INTER
A/98/OG	A/203/O2A	0	0	1	0	0	0	0	1	0	INTER
A/203/O3B	A/389/O	1	0	1	0	0	0	0	1	0	SELECTION_WATER
A/20/N	A/203/O6	0	1	1	0	0	0	0	1	0	INTER
A/19/CA	A/203/O6	0	0	0	1	0	0	0	0	0	INTER
A/156/CE	A/203/O6	1	0	0	0	0	0	0	0	1	INTER
A/156/NZ	A/203/O6	1	0	1	0	0	0	0	1	0	INTER
A/156/CD	A/203/O6	0	0	0	0	0	0	0	0	1	INTER
A/156/CE	A/203/N7	1	0	0	0	0	0	0	0	1	INTER
A/156/NZ	A/203/N7	1	0	1	0	0	0	0	1	0	INTER
A/201/NA	A/203/C3'	1	0	0	0	0	0	0	0	0	INTER
A/201/NA	A/203/O2'	0	0	0	0	0	1	0	0	0	INTER
A/201/NA	A/203/C2'	1	0	0	0	0	0	0	0	0	INTER

A/203/O2D	A/81/NZ	0	0	0	0	1	0	0	0	0	INTER
A/201/NA	A/203/O3C	1	0	0	0	0	0	0	0	0	INTER
A/201/NA	A/203/PC	1	0	0	0	0	0	0	0	0	INTER
A/202/NA	A/203/PC	1	0	0	0	0	0	0	0	0	INTER
A/203/O2D	A/59/N	1	0	1	0	0	0	0	1	0	INTER
A/203/O2C	A/81/CE	0	0	0	0	0	0	0	0	1	INTER
A/203/O2C	A/353/O	1	0	1	0	0	0	0	1	0	SELECTION_WATER
A/81/NZ	A/203/O1D	0	0	0	0	1	0	0	0	0	INTER
A/203/O1D	A/396/O	1	0	1	0	0	0	0	1	0	SELECTION_WATER
A/203/O3C	A/81/NZ	0	0	0	0	0	0	0	1	0	INTER
A/203/O2C	A/81/NZ	1	0	1	0	0	0	0	1	0	INTER
A/321/O	A/203/O2C	1	0	1	0	0	0	0	1	0	SELECTION_WATER
A/203/O2D	A/347/O	1	0	1	0	0	0	0	1	0	SELECTION_WATER
A/203/O1C	A/347/O	0	1	1	0	0	0	0	1	0	SELECTION_WATER
A/203/O1C	A/202/NA	0	0	0	0	0	1	0	0	0	INTER
A/203/O3D	A/60/OG	1	0	1	0	0	0	0	1	0	INTER
A/203/O3D	A/60/CB	0	0	0	0	0	0	0	0	1	INTER
A/203/O2D	A/60/N	1	0	1	0	0	0	0	1	0	INTER
A/203/O2D	A/60/CB	0	0	0	0	0	0	0	0	0	INTER
A/27/ND2	A/203/O3D	0	0	0	0	0	0	0	0	0	INTER
B/303/O3C	B/19/CE	1	0	0	0	0	0	0	0	1	INTER
B/303/O2D	B/16/CA	1	0	0	0	0	0	0	0	1	INTER
B/303/O2D	B/16/N	1	0	1	0	0	0	0	1	0	INTER
B/301/MG	B/303/PC	1	0	0	0	0	0	0	0	0	INTER
B/303/O2D	B/558/O	0	1	1	0	0	0	0	1	0	SELECTION_WATER
B/301/MG	B/303/O2C	0	0	0	0	0	1	0	0	0	INTER
B/302/K	B/303/C6	0	1	0	0	0	0	0	0	0	INTER
B/302/K	B/303/O6	0	1	0	0	0	0	0	0	0	INTER
B/303/O3G	B/558/O	1	0	1	0	0	0	0	1	0	SELECTION_WATER
B/303/O3G	B/78/OH	0	1	1	0	0	0	0	1	0	INTER
B/303/C5'	B/78/CE2	0	1	0	0	0	0	0	0	0	INTER
B/39/OG	B/303/O6	1	0	1	0	0	0	0	1	0	INTER
B/39/CB	B/303/O6	1	0	0	0	0	0	0	0	1	INTER

B/105/N	B/303/N3	0	1	1	0	0	0	0	1	0	INTER
B/104/CG2	B/303/N1	0	0	0	0	0	0	0	0	1	INTER
B/104/CG2	B/303/C6	0	1	0	0	0	0	0	0	0	INTER
B/74/CD	B/303/N1	0	1	0	0	0	0	0	0	0	INTER
B/15/CA	B/303/O1C	0	1	0	1	0	0	0	0	1	INTER
B/406/O	B/303/O1C	1	0	1	0	0	0	0	1	0	SELECTION_WATER
B/303/O1C	B/19/NZ	1	0	1	0	0	0	0	1	0	INTER
B/303/PC	B/19/NZ	0	1	0	0	0	0	0	0	0	INTER
B/303/O1C	B/19/CE	0	0	0	0	0	0	0	0	1	INTER
B/303/N2	B/105/OE1	1	0	1	0	0	0	0	1	0	INTER
B/105/CG	B/303/N3	0	0	0	1	0	0	0	0	0	INTER
B/78/OH	B/303/O2C	1	0	0	0	0	0	0	1	0	INTER
B/558/O	B/303/O2C	1	0	1	0	0	0	0	1	0	SELECTION_WATER
B/74/OE1	B/303/N1	1	0	0	0	0	0	0	1	0	INTER
B/74/OE2	B/303/N1	0	0	0	0	0	0	0	1	0	INTER
B/303/N2	B/74/OE2	1	0	1	0	0	0	0	1	0	INTER
B/84/C	B/303/O6	0	1	0	0	0	0	0	0	0	INTER
B/84/CA	B/303/O6	0	0	0	0	0	0	0	0	1	INTER
B/84/N	B/303/O6	0	0	0	0	0	0	0	1	0	INTER
B/559/O	B/303/O3G	0	0	0	0	0	0	0	1	0	SELECTION_WATER
B/559/O	B/303/O2G	1	0	1	0	0	0	0	1	0	SELECTION_WATER
B/301/MG	B/303/O1B	1	0	0	0	1	1	0	0	0	INTER
B/301/MG	B/303/O3G	1	0	0	0	1	1	0	0	0	INTER
B/301/MG	B/303/O2G	0	0	0	0	1	0	0	0	0	INTER
B/301/MG	B/303/PG	1	0	0	0	0	0	0	0	0	INTER
B/560/O	B/303/O1B	1	0	1	0	0	0	0	1	0	SELECTION_WATER
B/301/MG	B/303/PB	1	0	0	0	0	0	0	0	0	INTER
B/506/O	B/303/O2B	0	1	1	0	0	0	0	1	0	SELECTION_WATER
B/560/O	B/303/O2B	1	0	1	0	0	0	0	1	0	SELECTION_WATER
B/560/O	B/303/PB	1	0	0	0	0	0	0	0	0	SELECTION_WATER
B/303/N9	B/83/CD2	0	0	0	0	0	0	1	0	0	INTER
B/302/K	B/303/C5	1	0	0	0	0	0	0	0	0	INTER
B/302/K	B/303/C8	1	0	0	0	0	0	0	0	0	INTER

B/302/K	B/303/N7	1	0	0	0	0	0	0	0	0	INTER
B/411/O	B/303/C8	0	0	0	0	0	0	0	0	1	SELECTION_WATER
B/19/CG	B/303/O3D	0	0	0	1	0	0	0	0	0	INTER
B/20/N	B/303/O1D	1	0	1	0	0	0	0	1	0	INTER
B/303/O3D	B/19/N	1	0	1	0	0	0	0	1	0	INTER
B/303/PD	B/560/O	1	0	0	0	0	0	0	0	0	SELECTION_WATER
B/303/O3C	B/560/O	0	0	1	0	0	0	0	1	0	SELECTION_WATER
B/303/O2D	B/445/O	0	0	0	0	0	0	0	0	0	SELECTION_WATER
B/303/O2D	B/525/O	1	0	1	0	0	0	0	1	0	SELECTION_WATER
B/301/MG	B/303/O1D	0	0	0	0	1	0	0	0	0	INTER
B/303/O3D	B/18/N	1	0	1	0	0	0	0	1	0	INTER
B/559/O	B/303/O2D	0	0	0	0	0	0	0	1	0	SELECTION_WATER
B/301/MG	B/303/PD	1	0	0	0	0	0	0	0	0	INTER
B/301/MG	B/303/O3C	0	1	0	0	0	0	0	0	0	INTER
B/301/MG	B/303/O2D	0	0	0	0	0	1	0	0	0	INTER
B/303/O1D	B/19/N	0	0	0	0	0	0	0	1	0	INTER
B/303/O1D	B/445/O	1	0	1	0	0	0	0	1	0	SELECTION_WATER
B/560/O	B/303/O1D	1	0	1	0	0	0	0	1	0	SELECTION_WATER
B/303/O2'	B/103/OE2	1	0	0	0	0	0	0	1	0	INTER
B/303/C2'	B/103/OE2	0	0	0	0	0	0	0	0	1	INTER
B/55/OH	B/303/O5'	0	0	0	0	0	0	0	1	0	INTER
B/55/OH	B/303/O1A	1	0	0	0	0	0	0	1	0	INTER
B/303/O2A	B/43/NH2	0	0	0	0	1	0	0	0	0	INTER
B/303/O3A	B/46/NH1	0	0	1	0	0	0	0	1	0	INTER
B/303/O1G	B/46/NH1	0	0	0	0	1	0	0	0	0	INTER
B/303/O1G	B/46/NH2	0	0	1	0	1	0	0	0	0	INTER
B/303/O2A	B/46/NH1	0	0	0	0	1	0	0	0	0	INTER
B/303/O2A	B/43/CZ	0	0	0	0	1	0	0	0	0	INTER
B/303/O2A	B/43/NE	1	0	1	0	1	0	0	1	0	INTER
B/303/O2A	B/46/NH2	0	0	0	0	1	0	0	0	0	INTER
B/526/O	B/303/O3A	0	0	0	0	0	0	0	1	0	SELECTION_WATER
B/526/O	B/303/O1G	1	0	1	0	0	0	0	1	0	SELECTION_WATER
B/526/O	B/303/O2A	1	0	1	0	0	0	0	1	0	SELECTION_WATER

	B/83/OH	B/303/O2A	1	0	1	0	0	0	0	1	0	INTER
	B/43/CZ	B/303/O1A	0	0	0	0	1	0	0	0	0	INTER
	B/43/NE	B/303/O1A	0	0	0	0	1	0	0	0	0	INTER
	B/303/O1A	B/561/O	1	0	1	0	0	0	0	1	0	SELECTION_WATER
	B/43/NH2	B/303/O1A	1	0	1	0	1	0	0	1	0	INTER
	B/303/C8	B/55/OH	0	0	0	1	0	0	0	0	1	INTER
	B/303/C8	B/55/CE1	0	0	0	0	0	0	1	0	0	INTER
	B/303/N7	B/55/CE1	0	0	0	1	0	0	1	0	0	INTER
	B/303/N7	B/39/OG	0	0	0	0	0	0	0	1	0	INTER
	B/83/CD2	B/303/C8	0	0	0	0	0	0	1	0	0	INTER
	B/83/CD2	B/303/N7	0	0	0	0	0	0	1	0	0	INTER
5VOG	A/203/O2C	A/202/O1	1	0	1	0	0	0	0	1	0	INTER
	A/327/O	A/203/O2D	1	0	1	0	0	0	0	1	0	SELECTION_WATER
	A/203/O2C	A/317/O	1	0	1	0	0	0	0	1	0	SELECTION_WATER
	A/203/O2C	A/202/C1	0	1	0	0	0	0	0	0	1	INTER
	A/203/O2C	A/204/MG	1	0	0	0	1	1	0	0	0	INTER
	A/203/PC	A/204/MG	1	0	0	0	0	0	0	0	0	INTER
	A/203/O1A	A/105/OG1	1	0	0	0	0	0	0	1	0	INTER
	A/203/O1A	A/105/N	0	0	1	0	0	0	0	1	0	INTER
	A/203/O2C	A/202/O2	0	0	0	0	0	0	0	1	0	INTER
	A/203/C3'	A/202/O2	0	0	0	0	0	0	0	0	1	INTER
	A/203/O3D	A/352/O	1	0	1	0	0	0	0	1	0	SELECTION_WATER
	A/102/N	A/203/O2B	0	1	1	0	0	0	0	1	0	INTER
	A/203/O2'	A/202/O2	0	0	0	0	0	0	0	1	0	INTER
	A/203/O2'	A/98/OE2	1	0	0	0	0	0	0	1	0	INTER
	A/203/C2	A/156/CE2	0	0	0	0	0	0	1	0	0	INTER
	A/203/N1	A/156/CE2	0	0	0	0	0	0	1	0	0	INTER
	A/38/N	A/203/O1D	0	0	0	0	0	0	0	1	0	INTER
	A/316/O	A/203/O1D	1	0	1	0	0	0	0	1	0	SELECTION_WATER
	A/38/CA	A/203/O1D	0	0	0	0	0	0	0	0	0	INTER
	A/317/O	A/203/O1D	0	1	1	0	0	0	0	1	0	SELECTION_WATER
	A/202/C1	A/203/O1D	0	0	0	0	0	0	0	0	0	INTER
	A/204/MG	A/203/O1D	0	0	0	0	0	1	0	0	0	INTER

A/203/O3A	A/202/C2	0	0	0	0	0	0	0	0	1	INTER
A/203/O3B	A/99/O	1	0	0	0	0	0	0	0	0	INTER
A/103/CA	A/203/O2B	0	0	0	0	0	0	0	0	1	INTER
A/103/N	A/203/O2B	1	0	1	0	0	0	0	1	0	INTER
A/203/O3B	A/367/O	1	0	1	0	0	0	0	1	0	SELECTION_WATER
A/203/O3B	A/202/C1	0	0	0	1	0	0	0	0	0	INTER
A/203/O1B	A/105/CB	0	0	0	1	0	0	0	0	1	INTER
A/38/N	A/203/O2D	1	0	1	0	0	0	0	1	0	INTER
A/203/O3A	A/202/C1	1	0	0	0	0	0	0	0	1	INTER
A/203/PD	A/204/MG	1	0	0	0	0	0	0	0	0	INTER
A/106/N	A/203/O1B	1	0	1	0	0	0	0	1	0	INTER
A/203/O1B	A/105/CA	0	0	0	0	0	0	0	0	1	INTER
A/203/O1B	A/105/N	1	0	1	0	0	0	0	1	0	INTER
A/203/O1B	A/104/N	0	0	0	0	0	0	0	1	0	INTER
A/203/O1B	A/103/C	1	0	0	0	0	0	0	0	0	INTER
A/203/O1B	A/103/CA	1	0	0	0	0	0	0	0	1	INTER
A/103/N	A/203/O1B	0	0	0	0	0	0	0	1	0	INTER
A/39/N	A/203/O1D	1	0	1	0	0	0	0	1	0	INTER
A/202/O2	A/203/O1D	0	0	0	0	0	0	0	1	0	INTER
A/100/CA	A/203/O2B	0	0	0	1	0	0	0	0	1	INTER
A/101/N	A/203/O2B	1	0	1	0	0	0	0	1	0	INTER
A/203/O3'	A/351/O	0	0	0	0	0	0	0	1	0	SELECTION_WATER
A/203/O3D	A/351/O	1	0	1	0	0	0	0	1	0	SELECTION_WATER
A/203/C1'	A/398/O	0	0	0	1	0	0	0	0	0	SELECTION_WATER
A/203/O2A	A/101/CB	0	0	0	1	0	0	0	0	0	INTER
A/203/O3C	A/451/O	0	0	1	0	0	0	0	1	0	SELECTION_WATER
A/203/O3'	A/384/O	0	0	1	0	0	0	0	0	0	SELECTION_WATER
A/203/C4'	A/384/O	0	0	0	1	0	0	0	0	0	SELECTION_WATER
A/203/O3D	A/451/O	0	0	1	0	0	0	0	1	0	SELECTION_WATER
A/203/O2C	A/364/O	1	0	1	0	0	0	0	1	0	SELECTION_WATER
A/203/O1C	A/328/O	1	0	1	0	0	0	0	1	0	SELECTION_WATER
A/384/O	A/203/O1C	1	0	1	0	0	0	0	1	0	SELECTION_WATER
A/203/O1A	A/62/OH	1	0	0	0	0	0	0	1	0	INTER

A/203/O1A	A/62/CE2	0	0	0	0	0	0	0	0	1	INTER
A/203/O2A	A/102/CB	0	0	0	0	0	0	0	0	1	INTER
A/203/O2A	A/309/O	1	0	1	0	0	0	0	1	0	SELECTION_WATER
A/203/O2A	A/102/OG	1	0	1	0	0	0	0	1	0	INTER
A/102/N	A/203/O2A	1	0	1	0	0	0	0	1	0	INTER
A/445/O	A/203/O4'	0	1	1	0	0	0	0	1	0	SELECTION_WATER
A/153/CD2	A/203/N1	0	0	0	0	0	0	1	0	0	INTER
A/203/C5	A/153/CH2	0	0	0	0	0	0	1	0	0	INTER
A/203/C6	A/153/CH2	0	0	0	0	0	0	1	0	0	INTER
A/203/C6	A/153/CZ2	0	0	0	0	0	0	1	0	0	INTER
A/203/C6	A/153/CE2	0	0	0	0	0	0	1	0	0	INTER
A/153/CZ3	A/203/C4	0	0	0	0	0	0	1	0	0	INTER
A/203/C2	A/153/CH2	0	0	0	0	0	0	1	0	0	INTER
A/203/C2	A/153/CZ2	1	0	0	0	0	0	1	0	0	INTER
A/324/O	A/203/N2	1	0	1	0	0	0	0	1	0	SELECTION_WATER
A/203/C2	A/153/CE2	0	0	0	0	0	0	1	0	0	INTER
A/203/N1	A/153/CH2	0	0	0	0	0	0	1	0	0	INTER
A/203/N1	A/153/CZ2	0	0	0	0	0	0	1	0	1	INTER
A/203/N1	A/153/CE2	0	0	0	0	0	0	1	0	0	INTER
A/203/N1	A/153/NE1	0	0	0	0	0	0	1	0	0	INTER
A/358/O	A/203/N2	1	0	1	0	0	0	0	1	0	SELECTION_WATER
A/154/O	A/203/N2	1	0	1	0	0	0	0	1	0	INTER
A/154/O	A/203/C2	0	1	0	0	0	0	0	0	0	INTER
A/351/O	A/203/O2'	1	0	1	0	0	0	0	1	0	SELECTION_WATER
A/154/O	A/203/N1	1	0	1	0	0	0	0	1	0	INTER
A/203/N3	A/398/O	1	0	1	0	0	0	0	1	0	SELECTION_WATER
A/153/CH2	A/203/N9	0	0	0	0	0	0	1	0	0	INTER
A/153/CH2	A/203/C4	1	0	0	0	0	0	1	0	0	INTER
A/153/CH2	A/203/N3	0	0	0	0	0	0	1	0	0	INTER
A/153/CZ2	A/203/N9	0	0	0	0	0	0	0	0	0	INTER
A/153/CZ2	A/203/C4	0	0	0	0	0	0	1	0	0	INTER
A/153/CZ2	A/203/N3	0	0	0	0	0	0	1	0	1	INTER
A/203/N7	A/153/CZ3	0	0	0	0	0	0	1	0	0	INTER

	A/203/C5	A/153/CZ3	0	1	0	0	0	0	1	0	0	INTER
	A/203/C5	A/153/CE3	0	0	0	0	0	0	1	0	0	INTER
	A/203/C6	A/153/CZ3	0	0	0	0	0	0	1	0	0	INTER
	A/203/C6	A/153/CE3	0	0	0	0	0	0	1	0	0	INTER
	A/203/C6	A/153/CD2	0	0	0	0	0	0	1	0	0	INTER
	A/129/NZ	A/203/N7	0	1	1	0	0	0	0	1	0	INTER
	A/203/O6	A/129/NZ	1	0	1	0	0	0	0	1	0	INTER
	A/203/O6	A/129/CE	1	0	0	0	0	0	0	0	1	INTER
	A/154/N	A/203/O6	1	0	1	0	0	0	0	1	0	INTER
6CZF	A/47/CA	A/502/O3'	0	0	0	0	0	0	0	0	0	INTER
	A/45/NE	A/502/O3B	0	0	0	0	1	0	0	0	0	INTER
	A/45/CZ	A/502/O3B	0	0	0	0	1	0	0	0	0	INTER
	A/45/NH1	A/502/O3B	1	0	1	0	1	0	0	1	0	INTER
	A/45/NH1	A/502/O1B	0	0	0	0	1	0	0	1	0	INTER
	A/502/O3B	A/45/CD	0	1	0	0	0	0	0	0	1	INTER
	A/502/O3B	A/59/CD2	0	0	0	0	1	0	0	0	0	INTER
	A/62/CZ	A/502/O2B	0	0	0	0	1	0	0	0	0	INTER
	A/62/NH1	A/502/O2B	0	1	1	0	1	0	0	1	0	INTER
	A/502/O3B	A/59/NE2	0	0	0	0	1	0	0	0	0	INTER
	A/502/O3B	A/62/CZ	0	0	0	0	1	0	0	0	0	INTER
	A/502/O3B	A/62/NH1	1	0	1	0	1	0	0	1	0	INTER
	A/502/O2B	A/62/NH2	1	0	1	0	1	0	0	1	0	INTER
	A/502/O1C	A/45/NH2	0	1	1	0	1	0	0	1	0	INTER
	A/502/O1C	A/45/NH1	0	0	0	0	1	0	0	0	0	INTER
	A/502/O2C	A/45/CZ	0	0	0	0	1	0	0	0	0	INTER
	A/502/O2C	A/45/NH2	0	0	1	0	1	0	0	1	0	INTER
	A/502/O2C	A/45/NH1	1	0	1	0	1	0	0	1	0	INTER
	A/48/OD1	A/502/N3	0	0	1	0	0	0	0	1	0	INTER
	A/655/O	A/502/O1A	0	0	1	0	0	0	0	1	0	SELECTION_WATER
	A/83/O	A/502/N2	0	1	0	0	0	0	0	1	0	INTER
	A/82/O	A/502/N2	1	0	1	0	0	0	0	1	0	INTER
	A/502/N1	A/83/C	0	1	0	0	0	0	0	0	0	INTER
	A/502/N1	A/84/N	0	0	0	0	0	0	0	1	0	INTER

	A/82/O	A/502/N1	1	0	0	0	0	0	0	1	0	INTER
	A/82/C	A/502/N2	1	0	0	0	0	0	0	0	0	INTER
	A/502/N2	A/48/OD1	1	0	1	0	0	0	0	1	0	INTER
	A/502/N2	A/81/O	1	0	1	0	0	0	0	1	0	INTER
6D9S	A/131/NZ	A/201/O6	1	0	1	0	0	0	0	1	0	INTER
	A/103/OD2	A/201/C5	0	0	0	0	0	0	0	0	0	INTER
	A/201/C1'	A/343/O	0	0	0	1	0	0	0	0	0	SELECTION_WATER
	A/201/O2'	A/304/O	1	0	1	0	0	0	0	1	0	SELECTION_WATER
	A/338/O	A/201/O2D	1	0	1	0	0	0	0	1	0	SELECTION_WATER
	A/201/N3	A/343/O	1	0	1	0	0	0	0	1	0	SELECTION_WATER
	A/201/O1C	A/343/O	0	1	1	0	0	0	0	1	0	SELECTION_WATER
	A/201/O1C	A/325/O	1	0	1	0	0	0	0	1	0	SELECTION_WATER
	A/346/O	A/201/O2C	0	0	0	0	0	0	0	1	0	SELECTION_WATER
	A/201/O1C	A/202/MG	0	0	0	0	0	1	0	0	0	INTER
	A/201/N1	A/153/O	0	1	0	0	0	0	0	1	0	INTER
	A/201/N2	A/153/O	0	0	1	0	0	0	0	1	0	INTER
	A/201/O6	A/152/CA	0	0	0	1	0	0	0	0	0	INTER
	A/201/O6	A/153/N	0	0	1	0	0	0	0	1	0	INTER
	A/153/CG2	A/201/O6	0	0	0	1	0	0	0	0	0	INTER
	A/152/CE1	A/201/C6	0	0	0	0	0	0	1	0	0	INTER
	A/152/CE1	A/201/N1	0	1	0	0	0	0	1	0	1	INTER
	A/152/CZ	A/201/N1	0	0	0	0	0	0	1	0	0	INTER
	A/152/CE1	A/201/C2	1	0	0	0	0	0	1	0	0	INTER
	A/152/CE1	A/201/N3	0	0	0	0	0	0	1	0	0	INTER
	A/152/CZ	A/201/C2	0	0	0	0	0	0	1	0	0	INTER
	A/152/CZ	A/201/N2	0	1	0	0	0	0	0	0	0	INTER
	A/336/O	A/201/O1C	0	0	1	0	0	0	0	1	0	SELECTION_WATER
	A/159/O	A/201/N2	1	0	1	0	0	0	0	1	0	INTER
	A/201/C2	A/152/CD1	0	0	0	0	0	0	1	0	0	INTER
	A/201/C6	A/152/CD1	0	0	0	0	0	0	1	0	0	INTER
	A/201/N1	A/152/CD1	0	0	0	0	0	0	1	0	0	INTER
	A/201/O1C	A/346/O	1	0	1	0	0	0	0	1	0	SELECTION_WATER
	A/201/O1D	A/159/OD1	1	0	0	0	0	0	0	0	0	INTER

A/201/O1D	A/165/NH1	1	0	1	0	1	0	0	1	0	INTER
A/201/O2D	A/165/NH1	0	0	0	0	1	0	0	0	0	INTER
A/201/O3D	A/165/NH1	0	0	0	0	1	0	0	0	0	INTER
A/201/O1D	A/343/O	1	0	1	0	0	0	0	1	0	SELECTION_WATER
A/201/O2D	A/304/O	1	0	1	0	0	0	0	1	0	SELECTION_WATER
A/201/O2D	A/44/CA	0	0	0	0	0	0	0	0	1	INTER
A/201/O2D	A/44/N	1	0	1	0	0	0	0	1	0	INTER
A/325/O	A/201/O1D	1	0	1	0	0	0	0	1	0	SELECTION_WATER
A/351/O	A/201/O1D	0	0	1	0	0	0	0	1	0	SELECTION_WATER
A/351/O	A/201/O3D	0	1	1	0	0	0	0	1	0	SELECTION_WATER
A/165/NH2	A/201/O1D	1	0	1	0	1	0	0	1	0	INTER
A/165/CZ	A/201/O1D	0	0	0	0	1	0	0	0	0	INTER
A/165/NH2	A/201/O3D	0	0	0	0	1	0	0	0	0	INTER
A/201/PC	A/202/MG	1	0	0	0	0	0	0	0	0	INTER
A/201/O3C	A/202/MG	0	0	0	0	0	0	0	0	0	INTER
A/201/O1D	A/202/MG	1	0	0	0	1	1	0	0	0	INTER
A/201/PD	A/202/MG	0	1	0	0	0	0	0	0	0	INTER
A/43/N	A/201/O2D	0	0	1	0	0	0	0	1	0	INTER
A/43/N	A/201/O3D	1	0	1	0	0	0	0	1	0	INTER
A/43/CG	A/201/O3D	0	0	0	1	0	0	0	0	0	INTER
A/201/O3B	A/323/O	1	0	1	0	0	0	0	1	0	SELECTION_WATER
A/201/O1B	A/323/O	1	0	1	0	0	0	0	1	0	SELECTION_WATER
A/201/PB	A/323/O	1	0	0	0	0	0	0	0	0	SELECTION_WATER
A/201/O3A	A/103/CB	0	1	0	1	0	0	0	0	1	INTER
A/201/O3A	A/104/N	1	0	1	0	0	0	0	1	0	INTER
A/105/N	A/201/O3B	1	0	1	0	0	0	0	1	0	INTER
A/201/N7	A/103/CB	0	0	0	1	0	0	0	0	0	INTER
A/101/CD1	A/201/N7	0	0	0	1	0	0	0	0	0	INTER
A/201/O3B	A/104/N	1	0	1	0	0	0	0	1	0	INTER
A/201/O3B	A/103/N	1	0	1	0	0	0	0	1	0	INTER
A/201/O1B	A/105/N	0	0	0	0	0	0	0	1	0	INTER
A/201/O3A	A/104/OG	0	0	0	0	0	0	0	1	0	INTER
A/201/O1B	A/104/OG	1	0	0	0	0	0	0	1	0	INTER

	A/201/PB	A/104/OG	0	1	0	0	0	0	0	0	0	INTER
	A/301/O	A/201/O1A	1	0	1	0	0	0	0	1	0	SELECTION_WATER
	A/201/O1A	A/104/OG	0	0	0	0	0	0	0	1	0	INTER
	A/301/O	A/201/O1B	1	0	1	0	0	0	0	1	0	SELECTION_WATER
	A/323/O	A/201/O2B	0	0	1	0	0	0	0	0	0	SELECTION_WATER
6GFM	A/346/OH	A/501/C4	0	0	0	0	0	0	0	0	0	INTER
	A/346/CZ	A/501/C4	0	0	0	0	0	0	1	0	0	INTER
	A/346/CZ	A/501/C5	0	0	0	0	0	0	1	0	0	INTER
	A/346/CZ	A/501/C6	0	0	0	0	0	0	1	0	0	INTER
	A/346/CZ	A/501/N3	0	0	0	0	0	0	1	0	0	INTER
	A/346/CZ	A/501/C2	0	0	0	0	0	0	1	0	0	INTER
	A/346/CZ	A/501/N1	0	0	0	0	0	0	1	0	0	INTER
	A/346/CE1	A/501/C2	0	0	0	0	0	0	1	0	0	INTER
	A/346/CE1	A/501/N1	0	0	0	0	0	0	1	0	0	INTER
	A/501/O3A	A/601/O	0	0	1	0	0	0	0	1	0	SELECTION_WATER
	A/501/PB	A/601/O	1	0	0	0	0	0	0	0	0	SELECTION_WATER
	A/501/PA	A/601/O	1	0	0	0	0	0	0	0	0	SELECTION_WATER
	A/501/O3B	A/601/O	1	0	1	0	0	0	0	1	0	SELECTION_WATER
	A/501/O1A	A/601/O	0	0	1	0	0	0	0	1	0	SELECTION_WATER
	A/501/O2B	A/601/O	1	0	1	0	0	0	0	1	0	SELECTION_WATER
	A/501/C5'	A/601/O	1	0	0	0	0	0	0	0	1	SELECTION_WATER
	A/501/O5'	A/601/O	1	0	1	0	0	0	0	1	0	SELECTION_WATER
	A/609/O	A/501/O2G	1	0	1	0	0	0	0	1	0	SELECTION_WATER
	A/501/PG	A/601/O	1	0	0	0	0	0	0	0	0	SELECTION_WATER
	A/340/NH1	A/501/O2D	0	1	1	0	1	0	0	1	0	INTER
	A/340/CZ	A/501/O2D	0	1	0	0	1	0	0	0	0	INTER
	A/340/CZ	A/501/O1C	0	0	0	0	1	0	0	0	0	INTER
	A/501/O3D	A/340/NH2	0	0	0	0	1	0	0	0	0	INTER
	A/654/O	A/501/O3D	0	1	1	0	0	0	0	1	0	SELECTION_WATER
	A/501/O3D	A/340/NH1	0	0	0	0	1	0	0	0	0	INTER
	A/501/O3D	A/340/CZ	0	0	0	0	1	0	0	0	0	INTER
	A/346/OH	A/501/O2'	0	0	1	0	0	0	0	1	0	INTER
	A/656/O	A/501/O2'	0	0	0	0	0	0	0	0	0	SELECTION_WATER

A/501/PC	A/340/NH2	0	1	0	0	0	0	0	0	0	INTER
A/340/NH2	A/501/O2D	1	0	1	0	1	0	0	1	0	INTER
A/501/PD	A/340/NH2	0	1	0	0	0	0	0	0	0	INTER
A/501/O1C	A/340/NH2	1	0	1	0	1	0	0	1	0	INTER
A/667/O	A/501/O2A	0	0	0	0	0	0	0	1	0	SELECTION_WATER
A/636/O	A/501/O1G	1	0	1	0	0	0	0	1	0	SELECTION_WATER
A/501/O1A	A/688/O	0	0	0	0	0	0	0	1	0	SELECTION_WATER
A/501/O1B	A/683/O	0	0	0	0	0	0	0	1	0	SELECTION_WATER
A/639/O	A/501/O2B	1	0	1	0	0	0	0	1	0	SELECTION_WATER
A/346/N	A/501/O6	1	0	1	0	0	0	0	1	0	INTER
A/345/CA	A/501/O6	0	0	0	1	0	0	0	0	1	INTER
A/501/N1	A/346/CD1	0	0	0	0	0	0	1	0	0	INTER
A/501/N1	A/346/CG	0	0	0	0	0	0	1	0	0	INTER
A/346/CE2	A/501/N9	0	0	0	0	0	0	1	0	0	INTER
A/346/CE2	A/501/C4	0	0	0	0	0	0	1	0	0	INTER
A/346/CE2	A/501/C5	1	0	0	0	0	0	1	0	0	INTER
A/346/CE2	A/501/N7	0	0	0	0	0	0	1	0	0	INTER
A/346/CD2	A/501/C5	0	0	0	0	0	0	1	0	0	INTER
A/346/CD2	A/501/N7	0	0	0	0	0	0	1	0	0	INTER
A/346/CE2	A/501/C6	0	0	0	0	0	0	1	0	0	INTER
A/346/CD1	A/501/C6	0	0	0	0	0	0	1	0	0	INTER
A/346/CD2	A/501/C6	0	1	0	0	0	0	1	0	0	INTER
A/346/CG	A/501/C6	0	0	0	0	0	0	1	0	0	INTER
A/501/O6	A/616/O	1	0	1	0	0	0	0	1	0	SELECTION_WATER
A/628/O	A/501/O6	1	0	1	0	0	0	0	1	0	SELECTION_WATER
A/144/CA	A/501/N7	0	1	0	1	0	0	0	0	1	INTER
A/616/O	A/501/N7	1	0	1	0	0	0	0	1	0	SELECTION_WATER
A/344/O	A/501/N2	1	0	1	0	0	0	0	1	0	INTER
A/344/O	A/501/C2	1	0	0	0	0	0	0	0	0	INTER
A/344/O	A/501/N1	1	0	0	0	0	0	0	1	0	INTER
A/601/O	A/501/O2G	0	0	0	0	0	0	0	1	0	SELECTION_WATER
A/692/O	A/501/O2G	0	0	0	0	0	0	0	0	0	SELECTION_WATER
A/501/O3G	A/601/O	1	0	1	0	0	0	0	1	0	SELECTION_WATER

1SMY	N/9217/O	N/9100/N9	0	0	1	0	0	0	0	1	0	SELECTION_WATER
	N/9100/O1A	N/9208/O	0	0	0	0	0	0	0	1	0	SELECTION_WATER
	N/9211/O	N/9100/C2'	1	0	0	0	0	0	0	0	1	SELECTION_WATER
	N/9100/O4'	N/9208/O	0	1	1	0	0	0	0	1	0	SELECTION_WATER
	N/9100/O2C	N/9218/O	1	0	1	0	0	0	0	1	0	SELECTION_WATER
	N/9100/C3'	N/9212/O	0	0	0	0	0	0	0	0	1	SELECTION_WATER
	N/9100/O2'	N/9212/O	1	0	1	0	0	0	0	1	0	SELECTION_WATER
	N/9100/C2'	N/9212/O	0	0	0	0	0	0	0	0	1	SELECTION_WATER
	N/9100/O3C	N/9218/O	1	0	1	0	0	0	0	1	0	SELECTION_WATER
	N/2390/O	N/9100/O2D	1	0	1	0	0	0	0	1	0	SELECTION_WATER
	N/9100/PC	N/9218/O	1	0	0	0	0	0	0	0	0	SELECTION_WATER
	N/9100/O1D	N/9936/O	0	1	1	0	0	0	0	1	0	SELECTION_WATER
	N/9218/O	N/9100/O1D	0	1	1	0	0	0	0	1	0	SELECTION_WATER
	N/9100/O3D	N/9936/O	1	0	1	0	0	0	0	1	0	SELECTION_WATER
	N/9100/O3D	N/9218/O	0	0	0	0	0	0	0	0	0	SELECTION_WATER
	N/9100/PD	N/9936/O	1	0	0	0	0	0	0	0	0	SELECTION_WATER
	N/9100/PD	N/9218/O	0	1	0	0	0	0	0	0	0	SELECTION_WATER
	N/9100/O2D	N/9936/O	0	0	1	0	0	0	0	1	0	SELECTION_WATER
	N/9212/O	N/9100/O3C	0	0	0	0	0	0	0	1	0	SELECTION_WATER
	N/9212/O	N/9100/O3'	1	0	1	0	0	0	0	1	0	SELECTION_WATER
	N/9100/C8	N/9211/O	0	1	0	0	0	0	0	0	1	SELECTION_WATER
	N/9210/O	N/9100/O3A	0	0	1	0	0	0	0	0	0	SELECTION_WATER
	N/9210/O	N/9100/O3B	1	0	1	0	0	0	0	1	0	SELECTION_WATER
	N/9210/O	N/9100/PB	0	1	0	0	0	0	0	0	0	SELECTION_WATER
	N/9100/O2A	N/9217/O	0	0	1	0	0	0	0	1	0	SELECTION_WATER
	N/9100/O1A	N/9217/O	1	0	1	0	0	0	0	1	0	SELECTION_WATER
	N/9217/O	N/9100/N7	1	0	1	0	0	0	0	1	0	SELECTION_WATER
	N/9217/O	N/9100/C8	1	0	0	0	0	0	0	0	1	SELECTION_WATER
	N/9217/O	N/9100/C5	0	1	0	0	0	0	0	0	0	SELECTION_WATER
	N/9100/PA	N/9217/O	1	0	0	0	0	0	0	0	0	SELECTION_WATER
	N/9210/O	N/9100/O2A	1	0	1	0	0	0	0	1	0	SELECTION_WATER
	N/9100/O2B	N/9210/O	0	1	1	0	0	0	0	1	0	SELECTION_WATER
	N/9209/O	N/9100/O1A	0	1	1	0	0	0	0	1	0	SELECTION_WATER

	N/9209/O	N/9100/O2B	0	0	1	0	0	0	0	1	0	SELECTION_WATER
	N/9100/O2C	N/9211/O	0	0	0	0	0	0	0	1	0	SELECTION_WATER
	N/9100/C3'	N/9211/O	1	0	0	0	0	0	0	0	1	SELECTION_WATER
	N/9100/C5'	N/9211/O	1	0	0	0	0	0	0	0	1	SELECTION_WATER
	N/9100/C4'	N/9211/O	0	0	0	0	0	0	0	0	1	SELECTION_WATER
4JK1	D/1503/C4'	D/362/NH2	0	0	0	0	0	0	0	0	0	INTER
	D/417/NH1	D/1503/O1B	0	0	0	0	1	0	0	0	0	INTER
	D/1503/O1A	D/417/NH2	0	0	1	0	1	0	0	0	0	INTER
	D/1503/N2	D/615/CD	1	0	0	0	0	0	0	0	0	INTER
	D/615/CG	D/1503/N2	1	0	0	0	0	0	0	0	0	INTER
	D/615/NZ	D/1503/N3	0	0	1	0	0	0	0	1	0	INTER
	D/1503/O1D	D/362/NE	0	0	0	0	1	0	0	1	0	INTER
	D/1503/O1D	D/362/CG	0	0	0	1	0	0	0	0	0	INTER
	D/1503/O2D	D/362/NE	0	0	0	0	1	0	0	0	0	INTER
	D/1503/O2D	D/362/CD	0	0	0	1	0	0	0	0	1	INTER
	D/364/ND1	D/1503/O2D	0	0	0	0	1	0	0	0	0	INTER
	D/1503/O2D	D/364/CE1	0	0	0	0	1	0	0	0	0	INTER
	D/362/CZ	D/1503/O3C	0	1	0	0	0	0	0	0	0	INTER
	D/362/NE	D/1503/O3C	0	0	0	0	0	0	0	1	0	INTER
	D/619/CD1	D/1503/O3D	1	0	0	1	0	0	0	0	1	INTER
4JK2	D/1503/O5'	D/362/NH2	0	0	0	0	0	0	0	0	0	INTER
	D/1503/C5'	D/362/NH1	1	0	0	0	0	0	0	0	0	INTER
	D/1503/O2A	D/362/NH1	0	0	0	0	1	0	0	0	0	INTER
	D/1503/O2A	D/362/NH2	1	0	1	0	1	0	0	1	0	INTER
	D/1503/O2A	D/362/CZ	0	0	0	0	1	0	0	0	0	INTER
	D/1503/C3'	D/362/NH1	1	0	0	0	0	0	0	0	0	INTER
	D/1503/O3'	D/362/NH1	1	0	1	0	0	0	0	1	0	INTER
	D/1503/O2C	D/619/CD1	0	0	0	1	0	0	0	0	0	INTER
	D/1503/N3	D/615/NZ	0	0	1	0	0	0	0	0	0	INTER
	D/615/CD	D/1503/N2	1	0	0	0	0	0	0	0	0	INTER
	D/1503/O2'	D/615/NZ	1	0	1	0	0	0	0	1	0	INTER
	D/1503/O1C	D/615/NZ	0	0	0	0	1	0	0	0	0	INTER
	D/1503/O2C	D/615/NZ	0	0	0	0	1	0	0	0	0	INTER

	D/622/OD2	D/1503/O1D	0	1	0	0	0	0	0	0	0	INTER
	D/622/CG	D/1503/O1D	0	1	0	0	0	0	0	0	0	INTER
	D/1503/O2D	D/362/NH1	0	0	0	0	1	0	0	0	0	INTER
	D/1503/O2D	D/362/NH2	0	0	0	0	1	0	0	1	0	INTER
	D/1503/O2D	D/362/CZ	1	0	0	0	1	0	0	0	0	INTER
	D/1503/O2D	D/362/NE	1	0	0	0	1	0	0	1	0	INTER
	D/1503/PD	D/362/NE	0	1	0	0	0	0	0	0	0	INTER
	D/1503/PD	D/362/CD	1	0	0	0	0	0	0	0	0	INTER
	D/362/CD	D/1503/O3C	0	1	0	1	0	0	0	0	1	INTER
	D/1503/O1D	D/362/NE	0	0	0	0	1	0	0	1	0	INTER
	D/1503/O3C	D/362/NH1	1	0	0	0	0	0	0	1	0	INTER
	D/362/CZ	D/1503/O3C	1	0	0	0	0	0	0	0	0	INTER
	D/362/NE	D/1503/O3C	0	0	0	0	0	0	0	1	0	INTER
	D/1503/O1D	D/362/CD	1	0	0	0	0	0	0	0	1	INTER
	D/362/CG	D/1503/O1D	1	0	0	0	0	0	0	0	1	INTER
	D/619/CD1	D/1503/O3D	1	0	0	0	0	0	0	0	1	INTER
4JKR	D/619/CG1	D/2001/C6	0	0	0	0	0	0	0	0	0	INTER
	D/2001/O6	D/622/OD2	1	0	0	0	0	0	0	0	0	INTER
	D/2001/O6	D/619/CA	0	1	0	1	0	0	0	0	1	INTER
	D/2001/O3B	D/615/NZ	0	0	1	0	1	0	0	0	0	INTER
	D/2001/O2B	D/615/NZ	1	0	1	0	1	0	0	1	0	INTER
	D/2001/O2B	D/615/CE	0	0	0	0	0	0	0	0	0	INTER
	D/619/CG1	D/2001/N7	0	0	0	1	0	0	0	0	0	INTER
	D/2001/O2'	D/362/NH1	1	0	1	0	0	0	0	1	0	INTER
	D/2001/O3D	D/417/NH2	0	0	0	0	1	0	0	0	0	INTER
5TMC	D/1605/O6	D/737/ND2	0	0	0	0	0	0	0	0	0	INTER
	D/1606/MG	D/1605/O1C	0	1	0	0	0	0	0	0	0	INTER
	D/1605/N1	D/1231/OE2	0	0	0	0	0	0	0	1	0	INTER
	D/1605/N2	D/1740/O	0	0	1	0	0	0	0	1	0	SELECTION_WATER
	D/1605/C5	D/737/OD1	0	1	0	0	0	0	0	0	0	INTER
	D/1605/O1D	D/783/NH1	0	0	0	0	1	0	0	0	0	INTER
	D/1606/MG	D/1605/O2D	0	0	0	0	1	0	0	0	0	INTER
	D/1606/MG	D/1605/O1D	0	0	0	0	1	0	0	0	0	INTER

	D/1606/MG	D/1605/O3D	0	0	0	0	0	1	0	0	0	INTER
	D/1605/O2C	D/1730/O	0	1	1	0	0	0	0	1	0	SELECTION_WATER
	D/1605/O2C	D/1707/O	1	0	1	0	0	0	0	1	0	SELECTION_WATER
	D/1605/O1D	D/908/NZ	0	0	0	0	1	0	0	0	0	INTER
	D/1605/O3D	D/908/NZ	1	0	1	0	0	0	0	1	0	INTER
	D/1707/O	D/1605/O3D	0	0	0	0	0	0	0	1	0	SELECTION_WATER
	D/1605/PD	D/1606/MG	0	0	0	0	0	0	0	0	0	INTER
	D/1605/O3C	D/1606/MG	1	0	0	0	0	1	0	0	0	INTER
	D/1605/O2C	D/1606/MG	0	0	0	0	0	1	0	0	0	INTER
	D/1605/PC	D/1606/MG	1	0	0	0	0	0	0	0	0	INTER
	D/1605/O2D	D/783/NH1	0	0	1	0	1	0	0	1	0	INTER
	D/1605/O2D	D/783/CZ	0	0	0	0	1	0	0	0	0	INTER
	D/783/NH2	D/1605/O2D	1	0	1	0	1	0	0	1	0	INTER
	D/1029/CZ	D/1605/O2B	0	0	0	0	1	0	0	0	0	INTER
	D/1029/NH1	D/1605/O2B	0	1	1	0	1	0	0	1	0	INTER
	D/1029/NH2	D/1605/O2B	0	0	1	0	1	0	0	1	0	INTER
	C/879/NH1	D/1605/O2B	0	0	0	0	1	0	0	0	0	INTER
	D/1605/O1B	C/879/NE	0	0	0	0	1	0	0	0	0	INTER
	D/1605/PA	D/1601/MG	1	0	0	0	0	0	0	0	0	INTER
	C/879/NH2	D/1605/O2B	0	0	0	0	1	0	0	0	0	INTER
	C/879/CZ	D/1605/O2B	0	0	0	0	1	0	0	0	0	INTER
	C/879/NE	D/1605/O2B	0	0	0	0	1	0	0	1	0	INTER
	D/1605/O1A	D/1729/O	0	0	0	0	0	0	0	1	0	SELECTION_WATER
	D/1605/O1A	D/1601/MG	0	0	0	0	0	1	0	0	0	INTER
	D/1605/O2B	D/1601/MG	1	0	0	0	1	0	0	0	0	INTER
	C/879/NE	D/1605/O3B	0	0	0	0	1	0	0	0	0	INTER
	C/878/CB	D/1605/O1B	1	0	0	0	0	0	0	0	1	INTER
	D/1733/O	D/1605/O1C	0	0	1	0	0	0	0	1	0	SELECTION_WATER
	D/1605/O1B	D/1029/NH1	0	0	0	0	1	0	0	0	0	INTER
	D/1605/O1B	C/878/OG	1	0	1	0	0	0	0	1	0	INTER
	C/879/CD	D/1605/O1B	0	0	0	1	0	0	0	0	0	INTER
	M/202/N3	D/680/CB	0	0	0	1	0	0	0	0	0	INTER
5VSW	M/202/C2	D/680/CB	1	0	0	0	0	0	0	0	0	INTER

M/202/C4'	M/98/NZ	1	0	0	0	0	0	0	0	0	INTER
M/91/CB	M/202/N3	0	0	0	1	0	0	0	0	0	INTER
M/202/O1D	M/91/NH2	0	0	0	0	1	0	0	0	0	INTER
M/202/O1D	M/91/CZ	0	1	0	0	1	0	0	0	0	INTER
M/202/O2C	M/91/NH2	0	0	0	0	1	0	0	0	0	INTER
M/202/O2C	M/91/CZ	1	0	0	0	1	0	0	0	0	INTER
M/202/O2C	M/91/NE	0	0	0	0	1	0	0	1	0	INTER
M/202/O2'	M/91/CD	0	0	0	1	0	0	0	0	1	INTER
M/202/N3	M/91/CD	0	1	0	1	0	0	0	0	1	INTER
M/202/N2	M/91/CD	1	0	0	0	0	0	0	0	0	INTER
M/202/O1D	M/91/NH1	1	0	1	0	1	0	0	1	0	INTER
M/202/O2'	M/91/NH1	1	0	1	0	0	0	0	1	0	INTER
M/202/O2C	M/91/NH1	1	0	0	0	1	0	0	1	0	INTER
M/94/NZ	M/202/O1C	1	0	1	0	1	0	0	1	0	INTER
M/94/NZ	M/202/O2C	0	0	0	0	1	0	0	0	0	INTER
M/94/NZ	M/202/PC	0	1	0	0	0	0	0	0	0	INTER
M/91/CG	M/202/O2C	0	0	0	1	0	0	0	0	0	INTER
M/202/O3'	M/94/NZ	0	0	1	0	0	0	0	0	0	INTER
D/680/CB	M/202/N1	0	0	0	1	0	0	0	0	1	INTER
D/680/CB	M/202/C6	0	1	0	0	0	0	0	0	0	INTER
D/680/ND2	M/202/C5	1	0	0	0	0	0	0	0	0	INTER
D/680/ND2	M/202/C6	1	0	0	0	0	0	0	0	0	INTER
D/680/ND2	M/202/N7	0	0	0	0	0	0	0	1	0	INTER
D/680/ND2	M/202/O6	1	0	1	0	0	0	0	1	0	INTER
D/684/OD1	M/202/N1	0	0	0	0	0	0	0	1	0	INTER
D/684/OD2	M/202/O6	0	1	0	0	0	0	0	0	0	INTER
M/95/CD1	M/202/O6	1	0	0	0	0	0	0	0	1	INTER
M/95/CG	M/202/N7	1	0	0	1	0	0	0	0	1	INTER
M/202/O4'	M/98/NZ	1	0	1	0	0	0	0	1	0	INTER
M/202/O5'	M/98/NZ	0	0	1	0	0	0	0	0	0	INTER
M/202/O2A	M/98/NZ	1	0	1	0	1	0	0	1	0	INTER
M/202/O2A	M/98/CE	1	0	0	0	0	0	0	0	1	INTER
M/139/NZ	M/202/O1B	1	0	1	0	1	0	0	1	0	INTER

	M/139/NZ	M/202/O3A	0	0	0	0	0	0	0	0	0	INTER
	M/139/NZ	M/202/O2B	0	0	1	0	1	0	0	0	0	INTER
	M/139/CE	M/202/O1B	1	0	0	0	0	0	0	0	1	INTER
	D/362/CD	E/101/C2	0	0	0	0	0	0	0	0	0	INTER
	D/364/NE2	E/101/N3	0	0	0	0	0	0	1	0	0	INTER
	E/3/CG	E/101/O3'	0	1	0	0	0	0	0	0	1	INTER
	E/4/N	E/101/O2C	0	1	1	0	0	0	0	1	0	INTER
	E/3/CG	E/101/O2C	1	0	0	1	0	0	0	0	1	INTER
	E/3/N	E/101/O2C	1	0	1	0	0	0	0	1	0	INTER
	E/3/CZ	E/101/O2B	0	0	0	0	1	0	0	0	0	INTER
	E/3/NE	E/101/O2B	0	0	0	0	1	0	0	1	0	INTER
	E/3/CD	E/101/O2B	0	0	0	0	0	0	0	0	1	INTER
	E/3/NH1	E/101/O3A	0	0	0	0	0	0	0	1	0	INTER
	D/615/NZ	E/101/O2'	0	1	1	0	0	0	0	1	0	INTER
	E/101/N1	D/619/CG1	0	1	0	1	0	0	0	0	1	INTER
	D/622/OD2	E/101/N2	1	0	1	0	0	0	0	1	0	INTER
	D/619/CD1	E/101/C5	0	1	0	0	0	0	0	0	0	INTER
	D/619/CD1	E/101/C6	1	0	0	0	0	0	0	0	0	INTER
	D/619/CG1	E/101/C6	0	1	0	0	0	0	0	0	0	INTER
	E/4/CG1	E/101/O2C	1	0	0	1	0	0	0	0	1	INTER
	E/4/CG1	E/101/O1C	0	0	0	0	0	0	0	0	1	INTER
	D/615/NZ	E/101/O1C	1	0	1	0	1	0	0	1	0	INTER
	E/101/O3D	E/2/N	1	0	1	0	0	0	0	1	0	INTER
	E/101/O2C	E/2/N	0	0	1	0	0	0	0	0	0	INTER
	E/101/O1D	E/2/N	0	0	1	0	0	0	0	1	0	INTER
	A/601/PA	A/24/CA	0	1	0	0	0	0	0	0	0	INTER
3VR1	A/601/O1A	A/24/CA	1	0	0	1	0	0	0	0	1	INTER
	A/601/O3A	A/24/CA	1	0	0	0	0	0	0	0	1	INTER
	A/601/O5'	A/24/CA	0	0	0	1	0	0	0	0	0	INTER
	A/601/O3A	A/24/N	1	0	1	0	0	0	0	1	0	INTER
	A/601/O2B	A/23/N	0	1	1	0	0	0	0	1	0	INTER
	A/601/O2B	A/24/N	1	0	1	0	0	0	0	1	0	INTER
	A/601/C5'	A/22/O	0	0	0	1	0	0	0	0	0	INTER

A/601/O2B	A/25/N	1	0	1	0	0	0	0	1	0	INTER
A/26/CB	A/601/O1A	0	0	0	1	0	0	0	0	1	INTER
A/26/N	A/601/O1A	0	0	0	0	0	0	0	1	0	INTER
A/24/C	A/601/O1A	0	1	0	0	0	0	0	0	0	INTER
A/26/N	A/601/O3B	1	0	1	0	0	0	0	1	0	INTER
A/25/N	A/601/O3B	0	0	0	0	0	0	0	1	0	INTER
A/601/O3B	A/26/OG1	1	0	0	0	0	0	0	1	0	INTER
A/601/O3B	A/26/CB	1	0	0	0	0	0	0	0	1	INTER
A/49/NZ	A/601/O2D	1	0	1	0	1	0	0	1	0	INTER
A/601/O2D	A/49/CE	1	0	0	0	0	0	0	0	1	INTER
A/601/O2A	A/49/CE	0	0	0	1	0	0	0	0	0	INTER
A/601/O1A	A/27/N	1	0	1	0	0	0	0	1	0	INTER
A/27/OG1	A/601/O1A	1	0	0	0	0	0	0	1	0	INTER
A/601/O2'	A/49/NZ	0	0	1	0	0	0	0	1	0	INTER
A/27/CB	A/601/O1A	0	0	0	0	0	0	0	0	1	INTER
A/22/N	A/601/O1B	1	0	1	0	0	0	0	1	0	INTER
A/22/CA	A/601/O1B	0	1	0	0	0	0	0	0	1	INTER
A/601/O1B	A/91/NE2	1	0	0	0	1	0	0	1	0	INTER
A/601/O1B	A/91/CE1	0	0	0	0	1	0	0	0	0	INTER
A/601/O2B	A/22/N	0	0	0	0	0	0	0	1	0	INTER
A/22/CA	A/601/O3A	0	0	0	1	0	0	0	0	0	INTER
A/142/CA	A/601/O6	0	0	0	1	0	0	0	0	0	INTER
A/142/CA	A/601/N1	0	0	0	1	0	0	0	0	0	INTER
A/601/N1	A/144/OD2	0	1	1	0	0	0	0	1	0	INTER
A/601/O6	A/258/N	1	0	1	0	0	0	0	1	0	INTER
A/601/O6	A/258/CG2	0	0	0	1	0	0	0	0	1	INTER
A/601/O6	A/256/OG	0	0	1	0	0	0	0	1	0	INTER
A/257/N	A/601/O6	1	0	1	0	0	0	0	1	0	INTER
A/141/OD1	A/601/O6	0	1	0	0	0	0	0	0	0	INTER
A/256/C	A/601/O6	0	1	0	0	0	0	0	0	0	INTER
A/256/CA	A/601/O6	0	0	0	0	0	0	0	0	1	INTER
A/141/ND2	A/601/N7	0	0	1	0	0	0	0	1	0	INTER
A/601/C6	A/258/CB	1	0	0	0	0	0	0	0	0	INTER

	A/601/C6	A/258/CG2	1	0	0	0	0	0	0	0	0	INTER
	A/601/N1	A/258/CG2	1	0	0	0	0	0	0	0	1	INTER
	A/601/C5	A/258/CB	1	0	0	0	0	0	0	0	0	INTER
	A/144/OD1	A/601/N1	1	0	1	0	0	0	0	1	0	INTER
	A/601/C2	A/144/OD2	0	1	0	0	0	0	0	0	0	INTER
	A/601/N2	A/144/OD2	1	0	1	0	0	0	0	1	0	INTER
4HNX	A/901/C5	B/85/CE1	0	0	0	0	0	0	0	0	0	INTER
	A/901/N9	B/85/CE1	0	0	0	0	0	0	1	0	0	INTER
	A/464/NH2	A/901/O2C	0	1	1	0	1	0	0	1	0	INTER
	A/464/CZ	A/901/O2C	0	0	0	0	1	0	0	0	0	INTER
	B/82/CB	A/901/O4'	0	1	0	0	0	0	0	0	1	INTER
	A/464/NH1	A/901/O2C	0	0	0	0	1	0	0	0	0	INTER
	A/464/NH2	A/901/O1C	0	0	1	0	1	0	0	0	0	INTER
	A/464/CZ	A/901/O1C	0	0	0	0	1	0	0	0	0	INTER
	A/464/NH1	A/901/O1C	1	0	1	0	1	0	0	1	0	INTER
	A/464/NH1	A/901/O3D	0	0	0	0	0	0	0	0	0	INTER
	B/81/CA	A/901/N3	0	0	0	1	0	0	0	0	1	INTER
	B/82/N	A/901/N3	1	0	1	0	0	0	0	1	0	INTER
	A/901/C4	B/85/CG	0	0	0	0	0	0	1	0	0	INTER
	A/901/C4	B/85/CD1	0	0	0	0	0	0	1	0	0	INTER
	B/81/CB	A/901/N2	1	0	0	0	0	0	0	0	0	INTER
	A/901/N1	B/85/CG	0	0	0	0	0	0	1	0	0	INTER
	A/901/N3	B/85/CG	0	0	0	0	0	0	1	0	0	INTER
	A/901/N3	B/85/CD1	0	0	0	0	0	0	1	0	0	INTER
	B/85/CD2	A/901/C6	0	0	0	0	0	0	1	0	0	INTER
	B/88/CA	A/901/O6	0	1	0	1	0	0	0	0	1	INTER
	B/85/CD2	A/901/C5	0	0	0	0	0	0	1	0	0	INTER
	B/85/CG	A/901/C2	0	0	0	0	0	0	1	0	0	INTER
	B/82/O	A/901/N2	1	0	1	0	0	0	0	1	0	INTER
	A/901/O2'	B/80/O	0	0	1	0	0	0	0	1	0	INTER
	A/901/C1'	B/82/CB	0	1	0	0	0	0	0	0	0	INTER
	A/901/O1B	A/426/NH2	0	0	1	0	1	0	0	1	0	INTER
	A/901/O1B	A/426/NE	0	0	1	0	1	0	0	0	0	INTER

	A/901/O1B	A/426/CZ	0	0	0	0	1	0	0	0	0	INTER
	A/901/O3B	A/426/NE	0	0	1	0	1	0	0	1	0	INTER
	A/460/CE	A/901/O2C	0	1	0	0	0	0	0	0	1	INTER
	A/429/NZ	A/901/O2D	0	0	0	0	1	0	0	0	0	INTER
	A/460/CE	A/901/O1D	0	1	0	0	0	0	0	0	1	INTER
	A/349/NZ	A/901/O1A	0	0	1	0	1	0	0	1	0	INTER
	A/426/NH2	A/901/O3A	0	0	1	0	0	0	0	1	0	INTER
	A/460/NZ	A/901/O2C	1	0	1	0	1	0	0	1	0	INTER
	B/85/CE1	A/901/O4'	0	0	0	0	0	0	0	0	1	INTER
	A/901/O1D	A/460/NZ	1	0	1	0	1	0	0	1	0	INTER
	A/457/NZ	A/901/O1D	0	1	1	0	1	0	0	1	0	INTER
	A/457/NZ	A/901/O2D	1	0	1	0	1	0	0	1	0	INTER
	A/901/O2B	A/429/NZ	0	0	1	0	1	0	0	1	0	INTER
	A/901/O3B	A/429/NZ	1	0	1	0	1	0	0	1	0	INTER
	A/430/CD2	A/901/O1B	0	0	0	0	1	0	0	0	0	INTER
	A/901/O1B	A/430/CE1	1	0	0	0	1	0	0	0	0	INTER
	A/430/NE2	A/901/O1B	1	0	0	0	1	0	0	1	0	INTER
	A/901/O3D	A/429/NZ	1	0	1	0	1	0	0	1	0	INTER
	A/429/NZ	A/901/O1C	0	0	1	0	1	0	0	1	0	INTER
	A/461/CE1	A/901/O3D	0	1	0	0	0	0	0	0	1	INTER
	A/461/CZ	A/901/O3D	0	0	0	0	0	0	0	0	0	INTER
	A/461/OH	A/901/O3D	1	0	1	0	0	0	0	1	0	INTER
4XPD	A/901/O3B	A/460/NZ	1	0	1	0	1	0	0	1	0	INTER
	A/901/O2B	B/83/CD	0	0	0	1	0	0	0	0	1	INTER
	A/901/O1D	A/460/NZ	0	1	1	0	1	0	0	1	0	INTER
	B/91/NZ	A/901/O1A	0	0	0	0	1	0	0	0	0	INTER
	B/91/NZ	A/901/O4'	0	0	1	0	0	0	0	1	0	INTER
	B/91/NZ	A/901/N3	0	1	1	0	0	0	0	1	0	INTER
	A/901/O1D	A/457/CE	1	0	0	0	0	0	0	0	1	INTER
	A/901/O3D	A/457/NZ	1	0	1	0	1	0	0	1	0	INTER
	A/901/O3D	A/426/CG	0	1	0	0	0	0	0	0	1	INTER
	A/901/O3D	A/426/NE	0	0	0	0	0	0	0	0	0	INTER
	A/901/O1D	A/457/NZ	0	1	1	0	1	0	0	1	0	INTER

	A/901/PD	A/457/NZ	0	1	0	0	0	0	0	0	0	INTER
	A/461/OH	A/901/O3D	0	0	1	0	0	0	0	1	0	INTER
	A/901/C4	B/85/OH	1	0	0	0	0	0	0	0	0	INTER
	A/901/N3	B/85/OH	0	1	0	0	0	0	0	1	0	INTER
	A/429/NZ	A/901/O2D	1	0	1	0	1	0	0	1	0	INTER
	A/429/NZ	A/901/O1C	1	0	1	0	1	0	0	1	0	INTER
	A/429/NZ	A/901/PC	1	0	0	0	0	0	0	0	0	INTER
	A/460/NZ	A/901/O2D	0	1	1	0	1	0	0	1	0	INTER
	A/901/O2C	A/429/NZ	1	0	1	0	1	0	0	1	0	INTER
	A/353/NE2	A/901/N1	0	0	1	0	0	0	0	0	0	INTER
	A/353/NE2	A/901/C6	0	1	0	0	0	0	0	0	0	INTER
	A/353/NE2	A/901/O6	0	0	1	0	0	0	0	1	0	INTER
	A/901/O6	A/353/OE1	0	1	0	0	0	0	0	0	0	INTER
4Y49	A/901/C6	A/468/NZ	1	0	0	0	0	0	0	0	0	INTER
	A/901/O6	A/468/NZ	1	0	1	0	0	0	0	1	0	INTER
	A/901/O1C	A/437/CB	1	0	0	1	0	0	0	0	1	INTER
2J4R	A/19/CB	A/1307/N7	0	0	0	0	0	0	0	0	0	INTER
	A/17/CA	A/1307/O1A	1	0	0	0	0	0	0	0	1	INTER
	A/18/N	A/1307/O1A	1	0	1	0	0	0	0	1	0	INTER
	A/18/CA	A/1307/O1A	0	0	0	0	0	0	0	0	1	INTER
	A/1307/O2B	A/17/C	1	0	0	0	0	0	0	0	0	INTER
	A/1307/O2A	A/20/N	0	0	1	0	0	0	0	1	0	INTER
	A/20/CB	A/1307/O2A	1	0	0	1	0	0	0	0	1	INTER
	A/20/N	A/1307/O1A	1	0	1	0	0	0	0	1	0	INTER
	A/19/N	A/1307/O1A	1	0	1	0	0	0	0	1	0	INTER
	A/19/CA	A/1307/O1A	0	0	0	0	0	0	0	0	1	INTER
	A/1307/O1A	A/17/C	1	0	0	0	0	0	0	0	0	INTER
	A/1307/O3A	A/17/CA	0	0	0	1	0	0	0	0	0	INTER
	A/1307/O1C	A/22/NH2	0	0	0	0	1	0	0	0	0	INTER
	A/1307/O1C	A/22/NH1	0	0	0	0	1	0	0	0	0	INTER
	A/1307/O2B	A/87/OG1	0	0	0	0	0	0	0	1	0	INTER
	A/1307/O2B	A/17/CA	1	0	0	0	0	0	0	0	1	INTER
	A/1307/O2B	A/18/N	1	0	1	0	0	0	0	1	0	INTER

	A/1307/O2B	A/18/CA	0	0	0	0	0	0	0	0	1	INTER
	A/18/CB	A/1307/O2B	0	1	0	0	0	0	0	0	1	INTER
	A/18/OG	A/1307/O2B	0	0	1	0	0	0	0	1	0	INTER
	A/1307/O2C	A/2079/O	1	0	1	0	0	0	0	1	0	SELECTION_WATER
	A/1307/O2C	A/267/NH2	0	0	1	0	1	0	0	1	0	INTER
	A/1307/O1D	A/267/NH2	1	0	1	0	1	0	0	1	0	INTER
	A/1307/O1D	A/267/CZ	0	1	0	0	1	0	0	0	0	INTER
	A/1307/O2'	A/266/NH1	0	0	1	0	0	0	0	1	0	INTER
	A/267/NH2	A/1307/O2'	1	0	1	0	0	0	0	1	0	INTER
	A/2070/O	A/1307/O2'	0	1	1	0	0	0	0	1	0	SELECTION_WATER
	A/2070/O	A/1307/N3	0	1	1	0	0	0	0	1	0	SELECTION_WATER
	A/266/NH2	A/1307/C8	1	0	0	0	0	0	0	0	0	INTER
	A/266/NH2	A/1307/N9	1	0	1	0	0	0	0	1	0	INTER
	A/266/NH2	A/1307/N7	1	0	1	0	0	0	0	1	0	INTER
	A/266/NH2	A/1307/C4	1	0	0	0	0	0	0	0	0	INTER
	A/266/NH2	A/1307/C5	1	0	0	0	0	0	0	0	0	INTER
	A/266/CZ	A/1307/C4	1	0	0	0	0	0	0	0	0	INTER
	A/266/CZ	A/1307/C5	1	0	0	0	0	0	0	0	0	INTER
	A/1307/N7	A/19/OD1	1	0	0	0	0	0	0	0	0	INTER
	A/1307/O2D	A/143/CA	0	0	0	1	0	0	0	0	0	INTER
	A/211/N	A/1307/O2D	0	0	1	0	0	0	0	1	0	INTER
	A/267/NH1	A/1307/O1D	1	0	1	0	1	0	0	1	0	INTER
6PC1	B/369/CE1	A/601/C2	0	0	0	0	0	0	1	0	0	INTER
	B/369/CZ	A/601/C2	0	0	0	0	0	0	1	0	0	INTER
	B/369/CZ	A/601/N1	0	0	0	0	0	0	1	0	0	INTER
	B/369/CZ	A/601/C6	0	0	0	0	0	0	1	0	0	INTER
	B/369/CD1	A/601/N9	0	0	0	0	0	0	1	0	0	INTER
	B/369/CD1	A/601/C8	0	0	0	0	0	0	1	0	0	INTER
	B/369/CD1	A/601/C4	0	0	0	0	0	0	1	0	0	INTER
	B/369/CD1	A/601/N7	0	0	0	0	0	0	1	0	0	INTER
	B/369/CD1	A/601/C5	0	0	0	0	0	0	1	0	0	INTER
	B/369/CE1	A/601/N9	0	0	0	0	0	0	1	0	0	INTER
	B/369/CE1	A/601/C8	0	0	0	0	0	0	1	0	0	INTER

B/369/CE1	A/601/C4	1	0	0	0	0	0	1	0	0	INTER
B/369/CE1	A/601/N7	0	0	0	0	0	0	1	0	0	INTER
B/369/CE1	A/601/C5	0	0	0	0	0	0	1	0	0	INTER
B/369/CG	A/601/N7	0	0	0	0	0	0	1	0	0	INTER
B/369/CZ	A/601/C4	0	0	0	0	0	0	1	0	0	INTER
B/369/CZ	A/601/C5	0	0	0	0	0	0	1	0	0	INTER
B/369/CE1	A/601/N3	0	0	0	0	0	0	1	0	0	INTER
B/369/CZ	A/601/N3	0	0	0	0	0	0	1	0	0	INTER
A/601/O3D	A/205/NH2	0	0	1	0	1	0	0	1	0	INTER
A/601/O2D	A/256/NH2	1	0	1	0	1	0	0	1	0	INTER
A/601/O2D	A/205/NH2	0	0	0	0	1	0	0	0	0	INTER
A/601/O2D	A/256/NE	0	0	1	0	1	0	0	1	0	INTER
A/601/O2D	A/256/CZ	0	0	0	0	1	0	0	0	0	INTER
A/601/O2D	A/752/O	0	0	1	0	0	0	0	1	0	SELECTION_WATER
A/601/O2C	A/209/NZ	1	0	1	0	1	0	0	1	0	INTER
A/601/O2C	A/751/O	1	0	1	0	0	0	0	1	0	SELECTION_WATER
A/601/O4'	B/369/CD1	0	0	0	0	0	0	0	0	1	INTER
A/601/O3B	A/701/O	1	0	1	0	0	0	0	1	0	SELECTION_WATER
A/601/C5'	A/701/O	0	0	0	1	0	0	0	0	1	SELECTION_WATER
A/601/O1B	B/404/NZ	0	0	0	0	1	0	0	0	0	INTER
A/601/O1B	A/703/O	1	0	1	0	0	0	0	1	0	SELECTION_WATER
A/701/O	A/601/PB	0	1	0	0	0	0	0	0	0	SELECTION_WATER
A/701/O	A/601/O3A	0	0	1	0	0	0	0	0	0	SELECTION_WATER
B/666/O	A/601/O3A	0	0	0	0	0	0	0	1	0	SELECTION_WATER
A/703/O	A/601/O2B	0	0	1	0	0	0	0	1	0	SELECTION_WATER
A/601/O2B	B/404/CE	0	0	0	0	0	0	0	0	1	INTER
A/601/O2B	B/404/NZ	0	0	1	0	1	0	0	1	0	INTER
A/703/O	A/601/PB	1	0	0	0	0	0	0	0	0	SELECTION_WATER
A/601/O2A	A/703/O	0	1	1	0	0	0	0	1	0	SELECTION_WATER
A/35/NZ	A/601/O1C	0	0	1	0	1	0	0	1	0	INTER
A/601/O3C	A/735/O	1	0	1	0	0	0	0	1	0	SELECTION_WATER
A/601/PD	A/735/O	1	0	0	0	0	0	0	0	0	SELECTION_WATER
A/601/O2D	A/735/O	1	0	1	0	0	0	0	1	0	SELECTION_WATER

	A/601/O3D	A/35/NZ	0	0	1	0	1	0	0	1	0	INTER
	A/601/O3D	A/16/NH1	1	0	1	0	1	0	0	1	0	INTER
	A/601/O3C	A/35/NZ	0	0	0	0	0	0	0	1	0	INTER
	A/601/O3D	A/16/CZ	1	0	0	0	1	0	0	0	0	INTER
	A/16/NH2	A/601/O3D	1	0	1	0	1	0	0	1	0	INTER
	A/601/O2D	A/16/NH2	1	0	1	0	1	0	0	1	0	INTER
	A/601/O2C	A/253/ND2	0	0	0	0	0	0	0	1	0	INTER
	A/735/O	A/601/O2C	0	0	0	0	0	0	0	1	0	SELECTION_WATER
	A/601/N2	A/283/O	1	0	1	0	0	0	0	1	0	INTER
	A/601/N7	A/222/CD1	0	0	0	1	0	0	0	0	0	INTER
	B/369/CE2	A/601/C5	0	0	0	0	0	0	1	0	0	INTER
	A/601/O6	A/744/O	1	0	1	0	0	0	0	1	0	SELECTION_WATER
	A/283/O	A/601/C2	0	1	0	0	0	0	0	0	0	INTER
	A/283/O	A/601/N1	1	0	1	0	0	0	0	1	0	INTER
	A/283/CA	A/601/N1	1	0	0	0	0	0	0	0	1	INTER
	A/283/CA	A/601/O6	0	0	0	1	0	0	0	0	1	INTER
	A/601/C2	B/369/OH	0	1	0	0	0	0	0	0	0	INTER
	A/601/N1	B/369/OH	0	0	0	0	0	0	0	1	0	INTER
	A/601/C6	B/369/CE2	0	0	0	0	0	0	1	0	0	INTER
	A/768/O	A/601/C8	0	0	0	0	0	0	0	0	1	SELECTION_WATER
	A/768/O	A/601/N7	1	0	1	0	0	0	0	1	0	SELECTION_WATER
	A/205/NH2	A/601/O1D	0	0	1	0	1	0	0	1	0	INTER
	A/209/NZ	A/601/O2'	0	1	1	0	0	0	0	1	0	INTER
	A/752/O	A/601/O1D	1	0	1	0	0	0	0	1	0	SELECTION_WATER
4EDT	A/502/C4'	A/753/O	0	0	0	0	0	0	0	0	0	SELECTION_WATER
	A/502/O4'	A/230/NZ	1	0	1	0	0	0	0	1	0	INTER
	A/502/C5'	A/892/O	0	0	0	0	0	0	0	0	1	SELECTION_WATER
	A/312/OD2	A/502/O1D	0	1	0	0	0	0	0	0	0	INTER
	A/504/MN	A/502/O1D	1	0	0	0	1	1	0	0	0	INTER
	A/502/C3'	A/892/O	0	0	0	1	0	0	0	0	0	SELECTION_WATER
	A/504/MN	A/502/PD	1	0	0	0	0	0	0	0	0	INTER
	A/504/MN	A/502/O3C	1	0	0	0	0	0	0	0	0	INTER
	A/829/O	A/502/O1C	0	0	1	0	0	0	0	0	0	SELECTION_WATER

A/829/O	A/502/O3C	0	0	0	0	0	0	0	0	0	SELECTION_WATER
A/502/O3D	A/685/O	0	1	1	0	0	0	0	1	0	SELECTION_WATER
A/502/O2C	A/312/OD2	1	0	0	0	0	0	0	0	0	INTER
A/504/MN	A/502/PC	1	0	0	0	0	0	0	0	0	INTER
A/504/MN	A/502/O2C	1	0	0	0	1	1	0	0	0	INTER
A/874/O	A/502/O2C	0	0	0	0	0	0	0	1	0	SELECTION_WATER
A/829/O	A/502/PC	1	0	0	0	0	0	0	0	0	SELECTION_WATER
A/829/O	A/502/O2C	0	1	1	0	0	0	0	1	0	SELECTION_WATER
A/829/O	A/502/O3'	1	0	1	0	0	0	0	1	0	SELECTION_WATER
A/651/O	A/502/O1D	1	0	1	0	0	0	0	1	0	SELECTION_WATER
A/892/O	A/502/O1D	0	0	1	0	0	0	0	0	0	SELECTION_WATER
A/502/O1D	A/314/CB	0	1	0	1	0	0	0	0	1	INTER
A/230/N	A/502/O6	0	1	1	0	0	0	0	1	0	INTER
A/228/OE1	A/502/N2	1	0	1	0	0	0	0	1	0	INTER
A/228/OE1	A/502/N1	0	0	1	0	0	0	0	1	0	INTER
A/502/N2	A/228/OE2	0	0	1	0	0	0	0	1	0	INTER
A/502/N1	A/228/OE2	1	0	1	0	0	0	0	1	0	INTER
A/502/N1	A/228/CD	0	1	0	0	0	0	0	0	0	INTER
A/680/O	A/502/O6	1	0	1	0	0	0	0	1	0	SELECTION_WATER
A/680/O	A/502/N7	0	0	0	0	0	0	0	1	0	SELECTION_WATER
A/230/NZ	A/502/O1B	1	0	1	0	1	0	0	1	0	INTER
A/222/NH1	A/502/O1B	0	0	1	0	1	0	0	1	0	INTER
A/502/O2B	A/146/NH2	0	0	1	0	1	0	0	1	0	INTER
A/502/O2B	A/146/CZ	0	0	0	0	1	0	0	0	0	INTER
A/503/MN	A/502/PA	1	0	0	0	0	0	0	0	0	INTER
A/502/O3B	A/726/O	0	0	0	0	0	0	0	1	0	SELECTION_WATER
A/502/O3B	A/503/MN	0	0	0	0	0	1	0	0	0	INTER
A/502/O3B	A/808/O	0	0	1	0	0	0	0	1	0	SELECTION_WATER
A/146/NH2	A/502/PB	1	0	0	0	0	0	0	0	0	INTER
A/502/O2A	A/753/O	0	0	1	0	0	0	0	0	0	SELECTION_WATER
A/715/O	A/502/O2A	1	0	1	0	0	0	0	1	0	SELECTION_WATER
A/502/O1B	A/753/O	1	0	1	0	0	0	0	1	0	SELECTION_WATER
A/503/MN	A/502/O2A	1	0	0	0	1	1	0	0	0	INTER

	A/502/PB	A/503/MN	1	0	0	0	0	0	0	0	0	INTER
	A/808/O	A/502/O1B	1	0	1	0	0	0	0	1	0	SELECTION_WATER
	A/808/O	A/502/PB	1	0	0	0	0	0	0	0	0	SELECTION_WATER
	A/146/NH2	A/502/O3B	1	0	1	0	0	0	0	1	0	INTER
	A/502/O1A	A/837/O	1	0	1	0	0	0	0	1	0	SELECTION_WATER
	A/502/O1A	A/709/O	0	0	1	0	0	0	0	0	0	SELECTION_WATER
	A/230/NZ	A/502/O5'	0	0	1	0	0	0	0	1	0	INTER
	A/502/O2B	A/231/OH	1	0	0	0	0	0	0	1	0	INTER
	A/502/O2B	A/222/NH1	1	0	1	0	1	0	0	1	0	INTER
	A/222/CZ	A/502/O2B	1	0	0	0	1	0	0	0	0	INTER
	A/222/NE	A/502/O2B	1	0	1	0	1	0	0	1	0	INTER
	A/146/NH1	A/502/O2B	1	0	1	0	1	0	0	1	0	INTER
	A/231/CE2	A/502/O3A	0	0	0	1	0	0	0	0	1	INTER
	A/851/O	A/502/O3A	0	0	0	0	0	0	0	1	0	SELECTION_WATER
4EDV	A/231/CZ	A/502/PB	0	0	0	0	0	0	0	0	0	INTER
	A/502/PG	A/504/MN	1	0	0	0	0	0	0	0	0	INTER
	A/502/O2G	A/751/O	1	0	1	0	0	0	0	1	0	SELECTION_WATER
	A/502/O3G	A/611/O	0	0	0	0	0	0	0	1	0	SELECTION_WATER
	A/502/O3G	A/504/MN	0	0	0	0	0	1	0	0	0	INTER
	A/230/NZ	A/502/O4'	1	0	1	0	0	0	0	1	0	INTER
	A/502/O4'	A/762/O	0	0	1	0	0	0	0	0	0	SELECTION_WATER
	A/231/OH	A/502/O1G	1	0	1	0	0	0	0	1	0	INTER
	A/502/O3B	A/231/OH	0	0	1	0	0	0	0	1	0	INTER
	A/502/O3B	A/231/CE2	0	0	0	1	0	0	0	0	1	INTER
	A/502/O1G	A/222/NE	1	0	1	0	1	0	0	1	0	INTER
	A/502/O1G	A/222/CZ	1	0	0	0	1	0	0	0	0	INTER
	A/502/O1G	A/222/NH2	1	0	1	0	1	0	0	1	0	INTER
	A/502/O1G	A/146/NH1	1	0	1	0	1	0	0	1	0	INTER
	A/502/O2G	A/222/NH2	0	0	1	0	1	0	0	1	0	INTER
	A/502/O3G	A/146/NH1	0	0	1	0	0	0	0	1	0	INTER
	A/146/CZ	A/502/O1G	0	0	0	0	1	0	0	0	0	INTER
	A/502/O2G	A/230/NZ	0	1	1	0	1	0	0	1	0	INTER
	A/828/O	A/502/O2G	1	0	1	0	0	0	0	1	0	SELECTION_WATER

A/502/O3G	A/146/NH2	1	0	1	0	0	0	0	1	0	INTER
A/502/O6	A/230/N	1	0	1	0	0	0	0	1	0	INTER
A/502/O6	A/228/OE2	1	0	0	0	0	0	0	0	0	INTER
A/502/O6	A/229/C	0	0	0	0	0	0	0	0	0	INTER
A/502/O6	A/229/CA	0	1	0	0	0	0	0	0	1	INTER
A/502/O6	A/719/O	0	1	1	0	0	0	0	1	0	SELECTION_WATER
A/502/N7	A/719/O	1	0	1	0	0	0	0	1	0	SELECTION_WATER
A/228/CD	A/502/N1	1	0	0	0	0	0	0	0	0	INTER
A/228/OE2	A/502/C6	1	0	0	0	0	0	0	0	0	INTER
A/228/OE2	A/502/N1	1	0	0	0	0	0	0	1	0	INTER
A/228/OE1	A/502/C2	0	1	0	0	0	0	0	0	0	INTER
A/228/OE1	A/502/N1	1	0	0	0	0	0	0	1	0	INTER
A/228/OE1	A/502/N2	1	0	1	0	0	0	0	1	0	INTER
A/706/O	A/502/O2A	1	0	1	0	0	0	0	1	0	SELECTION_WATER
A/503/MN	A/502/PA	1	0	0	0	0	0	0	0	0	INTER
A/708/O	A/502/C5'	0	0	0	0	0	0	0	0	1	SELECTION_WATER
A/813/O	A/502/C5'	1	0	0	1	0	0	0	0	1	SELECTION_WATER
A/813/O	A/502/C4'	0	0	0	0	0	0	0	0	1	SELECTION_WATER
A/829/O	A/502/O2B	1	0	1	0	0	0	0	1	0	SELECTION_WATER
A/503/MN	A/502/O3A	0	1	0	0	0	0	0	0	0	INTER
A/709/O	A/502/O2B	0	0	1	0	0	0	0	1	0	SELECTION_WATER
A/503/MN	A/502/O2A	0	0	0	0	0	1	0	0	0	INTER
A/709/O	A/502/O2A	0	0	0	0	0	0	0	1	0	SELECTION_WATER
A/502/O2B	A/503/MN	0	1	0	0	0	0	0	0	0	INTER
A/502/PB	A/503/MN	1	0	0	0	0	0	0	0	0	INTER
A/502/O1B	A/799/O	0	0	1	0	0	0	0	1	0	SELECTION_WATER
A/502/O1B	A/503/MN	0	0	0	0	0	1	0	0	0	INTER
A/502/O1B	A/708/O	0	0	1	0	0	0	0	1	0	SELECTION_WATER
A/611/O	A/502/O2B	1	0	1	0	0	0	0	1	0	SELECTION_WATER
A/504/MN	A/502/O2B	1	0	0	0	0	0	0	0	0	INTER
A/504/MN	A/502/PB	1	0	0	0	0	0	0	0	0	INTER
A/504/MN	A/502/O1B	1	0	0	0	0	1	0	0	0	INTER
A/502/O1C	A/505/MN	1	0	0	0	1	1	0	0	0	INTER

	A/502/PC	A/505/MN	1	0	0	0	0	0	0	0	0	INTER
	A/502/O3C	A/505/MN	1	0	0	0	0	0	0	0	0	INTER
	A/502/O3D	A/505/MN	1	0	0	0	1	0	0	0	0	INTER
	A/502/PD	A/505/MN	1	0	0	0	0	0	0	0	0	INTER
	A/314/CB	A/502/O2D	1	0	0	0	0	0	0	0	1	INTER
	A/706/O	A/502/O1A	0	0	1	0	0	0	0	0	0	SELECTION_WATER
	A/502/O2D	A/706/O	0	0	1	0	0	0	0	1	0	SELECTION_WATER
	A/505/MN	A/502/O2D	1	0	0	0	1	1	0	0	0	INTER
	A/692/O	A/502/O2D	0	0	1	0	0	0	0	1	0	SELECTION_WATER
	A/798/O	A/502/O1C	1	0	1	0	0	0	0	1	0	SELECTION_WATER
	A/798/O	A/502/C5'	0	0	0	0	0	0	0	0	1	SELECTION_WATER
	A/798/O	A/502/C4'	0	1	0	0	0	0	0	0	1	SELECTION_WATER
	A/798/O	A/502/O3'	0	0	0	0	0	0	0	1	0	SELECTION_WATER
	A/502/O3D	A/826/O	0	0	0	0	0	0	0	1	0	SELECTION_WATER
	A/826/O	A/502/O1C	1	0	1	0	0	0	0	1	0	SELECTION_WATER
	A/639/O	A/502/O3D	1	0	1	0	0	0	0	1	0	SELECTION_WATER
3N75	E/716/O1C	E/3272/O	1	0	1	0	0	0	0	1	0	SELECTION_WATER
	E/3271/O	E/716/C3'	0	0	0	1	0	0	0	0	0	SELECTION_WATER
	E/1832/O	E/716/O3D	1	0	1	0	0	0	0	1	0	SELECTION_WATER
	E/1832/O	E/716/PD	1	0	0	0	0	0	0	0	0	SELECTION_WATER
	E/1832/O	E/716/O2D	0	0	1	0	0	0	0	1	0	SELECTION_WATER
	E/206/NH2	E/716/O2D	0	0	1	0	1	0	0	1	0	INTER
	E/716/O3'	E/3265/O	0	0	0	0	0	0	0	1	0	SELECTION_WATER
	E/716/O2B	C/585/NH1	0	0	0	0	1	0	0	0	0	INTER
	C/585/NH2	E/716/O1A	0	0	0	0	1	0	0	1	0	INTER
	C/585/CZ	E/716/O1A	0	1	0	0	1	0	0	0	0	INTER
	E/716/N2	E/3271/O	0	0	1	0	0	0	0	1	0	SELECTION_WATER
	C/565/NH1	E/716/O3B	0	0	0	0	1	0	0	0	0	INTER
	E/716/O3A	E/3223/O	0	0	0	0	0	0	0	1	0	SELECTION_WATER
	E/716/O2B	E/3233/O	1	0	1	0	0	0	0	1	0	SELECTION_WATER
	C/585/NH2	E/716/O1B	0	0	0	0	1	0	0	0	0	INTER
	E/716/O2C	E/3227/O	1	0	1	0	0	0	0	1	0	SELECTION_WATER
	E/716/O2C	E/1864/O	0	0	1	0	0	0	0	1	0	SELECTION_WATER

E/716/O2C	E/206/NE	0	0	0	0	1	0	0	0	0	INTER
E/716/O2C	E/206/CZ	0	0	0	0	1	0	0	0	0	INTER
E/716/O2C	E/206/NH2	0	0	0	0	1	0	0	1	0	INTER
E/716/O3C	E/1864/O	0	0	1	0	0	0	0	1	0	SELECTION_WATER
E/716/O3C	E/206/NH2	0	0	0	0	0	0	0	1	0	INTER
E/716/PC	E/417/CE	0	1	0	0	0	0	0	0	0	INTER
E/716/PC	E/3271/O	0	1	0	0	0	0	0	0	0	SELECTION_WATER
E/716/O1C	E/3271/O	1	0	1	0	0	0	0	1	0	SELECTION_WATER
E/716/O3C	E/417/CE	0	0	0	1	0	0	0	0	1	INTER
E/716/O2C	E/417/NZ	0	1	1	0	1	0	0	1	0	INTER
E/716/O2C	E/417/CE	0	0	0	0	0	0	0	0	1	INTER
E/716/O2C	E/3271/O	0	0	0	0	0	0	0	1	0	SELECTION_WATER
E/716/O1C	E/3273/O	0	1	1	0	0	0	0	1	0	SELECTION_WATER
E/716/O3C	E/1832/O	0	0	0	0	0	0	0	1	0	SELECTION_WATER
E/206/NE	E/716/O3B	1	0	1	0	1	0	0	1	0	INTER
E/206/CZ	E/716/O3B	0	0	0	0	1	0	0	0	0	INTER
E/206/NH1	E/716/O3B	0	0	0	0	1	0	0	0	0	INTER
E/716/O3B	E/3234/O	1	0	1	0	0	0	0	1	0	SELECTION_WATER
E/716/O3B	E/3223/O	1	0	1	0	0	0	0	1	0	SELECTION_WATER
E/716/O3B	E/206/CD	0	0	0	0	0	0	0	0	1	INTER
E/716/O1B	E/206/NE	0	0	0	0	1	0	0	0	0	INTER
E/716/O1B	E/206/CZ	0	0	0	0	1	0	0	0	0	INTER
E/206/NH1	E/716/O1B	1	0	1	0	1	0	0	1	0	INTER
E/716/O3'	E/206/NH2	0	0	0	0	0	0	0	1	0	INTER
C/568/ND2	E/716/O3A	0	0	0	0	0	0	0	1	0	INTER
E/716/O2A	C/565/N	1	0	1	0	0	0	0	1	0	INTER
E/716/O2A	C/568/ND2	1	0	1	0	0	0	0	1	0	INTER
E/716/O2B	C/565/NH1	0	1	1	0	1	0	0	1	0	INTER
E/716/O2B	C/565/CG	0	1	0	0	0	0	0	0	1	INTER
C/585/NH2	E/716/O2B	1	0	1	0	1	0	0	1	0	INTER
C/585/CZ	E/716/O2B	0	0	0	0	1	0	0	0	0	INTER
E/716/N7	E/97/CD	0	0	0	1	0	0	0	0	1	INTER
C/564/CD2	E/716/O4'	0	0	0	0	0	0	0	0	1	INTER

	E/3272/O	E/716/C2'	0	0	0	1	0	0	0	0	0	SELECTION_WATER
	E/3269/O	E/716/O1D	1	0	1	0	0	0	0	1	0	SELECTION_WATER
	E/418/N	E/716/O3D	1	0	1	0	0	0	0	1	0	INTER
	E/418/CA	E/716/O3D	1	0	0	0	0	0	0	0	1	INTER
	E/3265/O	E/716/O2D	1	0	1	0	0	0	0	1	0	SELECTION_WATER
	E/2268/O	E/716/O3D	0	0	1	0	0	0	0	1	0	SELECTION_WATER
	E/3229/O	E/716/O5'	0	0	1	0	0	0	0	0	0	SELECTION_WATER
	E/716/O2'	E/3265/O	0	0	1	0	0	0	0	1	0	SELECTION_WATER
	E/3229/O	E/716/O4'	1	0	1	0	0	0	0	1	0	SELECTION_WATER
	E/3229/O	E/716/C1'	0	0	0	0	0	0	0	0	1	SELECTION_WATER
	E/716/C6	C/564/CD1	0	1	0	0	0	0	0	0	0	INTER
	E/97/NE	E/716/N7	0	0	0	0	0	0	0	1	0	INTER
	C/558/NH2	E/716/O6	1	0	1	0	0	0	0	1	0	INTER
	C/558/NH1	E/716/N7	1	0	1	0	0	0	0	1	0	INTER
	E/716/O6	E/2573/O	1	0	1	0	0	0	0	1	0	SELECTION_WATER
	C/585/NH1	E/716/O1A	1	0	1	0	1	0	0	1	0	INTER
6GTM	A/114/CD	A/600/O3'	0	0	0	0	0	0	0	0	0	INTER
	A/114/NE2	A/600/O1D	0	0	1	0	0	0	0	1	0	INTER
	A/600/O2'	A/143/N	0	1	1	0	0	0	0	1	0	INTER
	A/142/N	A/600/N3	0	0	0	0	0	0	0	1	0	INTER
	A/111/CB	A/600/O4'	0	0	0	1	0	0	0	0	0	INTER
	A/600/C5'	A/112/O	0	0	0	1	0	0	0	0	0	INTER
	A/600/C4'	A/112/O	0	0	0	0	0	0	0	0	1	INTER
	A/600/O4'	A/112/N	0	0	1	0	0	0	0	1	0	INTER
	A/600/C1'	A/141/O	0	0	0	1	0	0	0	0	0	INTER
	A/600/O2'	A/142/CA	0	0	0	1	0	0	0	0	1	INTER
	A/600/O2A	A/111/ND2	0	0	1	0	0	0	0	1	0	INTER
	A/600/N2	A/142/O	0	0	1	0	0	0	0	0	0	INTER
	A/600/O1C	A/143/CB	0	0	0	1	0	0	0	0	1	INTER
	A/600/O2C	A/143/NE	0	0	0	0	1	0	0	0	0	INTER
	A/600/O6	A/78/NH1	0	0	1	0	0	0	0	1	0	INTER
	A/188/OE1	A/600/N2	1	0	1	0	0	0	0	1	0	INTER
	A/188/OE1	A/600/C2	0	1	0	0	0	0	0	0	0	INTER

	A/188/OE1	A/600/N1	1	0	0	0	0	0	0	1	0	INTER
	A/120/ND1	A/301/O5'	0	1	1	0	0	0	0	1	0	INTER
	A/120/CE1	A/301/O5'	1	0	0	0	0	0	0	0	0	INTER
5DED	A/120/ND1	A/301/O4'	0	0	0	0	0	0	0	0	0	INTER
	A/120/CE1	A/301/C4'	0	0	0	0	0	0	1	0	0	INTER
	A/120/CE1	A/301/O4'	0	0	0	0	0	0	1	0	0	INTER
	A/120/ND1	A/301/O2A	0	0	1	0	1	0	0	1	0	INTER
	A/107/OH	A/301/O2B	0	1	0	0	0	0	0	1	0	INTER
	A/112/NZ	A/301/PB	0	0	0	0	0	0	0	0	0	INTER
	A/112/NZ	A/301/O2B	1	0	1	0	1	0	0	1	0	INTER
	A/112/CE	A/301/O2B	0	1	0	0	0	0	0	0	1	INTER
	A/301/N3	A/155/CD2	0	0	0	0	0	0	1	0	0	INTER
	A/59/NH2	A/301/O2G	0	0	0	0	1	0	0	0	0	INTER
	A/59/NH2	A/301/PG	0	1	0	0	0	0	0	0	0	INTER
	A/56/NZ	A/301/O3G	1	0	1	0	1	0	0	1	0	INTER
	A/301/O2A	A/139/OE2	1	0	0	0	0	0	0	0	0	INTER
	A/301/PA	A/139/OE2	1	0	0	0	0	0	0	0	0	INTER
	A/139/OE2	A/301/C5'	0	1	0	0	0	0	0	0	1	INTER
	A/301/C5'	A/139/OE1	0	0	0	1	0	0	0	0	1	INTER
	A/105/NH2	A/301/O2A	0	0	0	0	1	0	0	0	0	INTER
	A/301/O2G	A/60/CD	0	1	0	1	0	0	0	0	1	INTER
	A/301/O2G	A/56/NZ	0	0	0	0	1	0	0	1	0	INTER
	A/301/O2G	A/60/NZ	1	0	1	0	1	0	0	1	0	INTER
	A/301/O1A	A/56/NZ	0	0	1	0	1	0	0	1	0	INTER
	A/301/O1B	A/105/NE	0	0	0	0	1	0	0	0	0	INTER
	A/301/O2A	A/105/NH1	1	0	0	0	1	0	0	1	0	INTER
	A/301/O2A	A/105/CZ	1	0	0	0	1	0	0	0	0	INTER
	A/301/O2A	A/105/NE	0	0	0	0	1	0	0	1	0	INTER
	A/301/O2A	A/105/CD	0	0	0	1	0	0	0	0	1	INTER
	A/301/O2'	A/155/CD2	1	0	0	0	0	0	0	0	0	INTER
	A/301/O2C	A/155/CD2	0	0	0	0	1	0	0	0	0	INTER
	A/301/O2C	A/155/NE2	0	1	0	0	1	0	0	1	0	INTER
	A/301/O1D	A/155/NE2	0	0	0	0	1	0	0	0	0	INTER

A/301/O1D	A/155/CE1	0	0	0	0	1	0	0	0	0	INTER
A/301/O3C	A/48/CE	0	0	0	0	0	0	0	0	1	INTER
A/301/O2D	A/46/NE	0	0	1	0	1	0	0	0	0	INTER
A/155/CD2	A/301/O3D	0	0	0	0	1	0	0	0	0	INTER
A/155/NE2	A/301/O3D	1	0	0	0	1	0	0	1	0	INTER
A/155/CE1	A/301/O3D	0	0	0	0	1	0	0	0	0	INTER
A/56/CE	A/301/O3G	0	1	0	0	0	0	0	0	1	INTER
A/59/NH2	A/301/O1G	0	0	0	0	1	0	0	0	0	INTER
A/59/NH2	A/301/O3G	1	0	1	0	1	0	0	1	0	INTER
A/48/NZ	A/301/O1C	1	0	1	0	1	0	0	1	0	INTER
A/59/NH1	A/301/O1G	0	0	1	0	1	0	0	1	0	INTER
A/59/NH1	A/301/O3G	0	0	0	0	1	0	0	0	0	INTER
A/59/CZ	A/301/O1G	0	0	0	0	1	0	0	0	0	INTER
A/59/CZ	A/301/O3G	0	1	0	0	1	0	0	0	0	INTER
A/116/CE1	A/301/N3	0	0	0	0	0	0	1	0	0	INTER
A/116/CE1	A/301/C2	0	0	0	0	0	0	1	0	0	INTER
A/116/CG	A/301/C6	0	1	0	0	0	0	1	0	0	INTER
A/116/CG	A/301/N1	0	0	0	0	0	0	1	0	0	INTER
A/116/CD1	A/301/N3	0	0	0	0	0	0	1	0	0	INTER
A/116/CD1	A/301/C6	0	0	0	0	0	0	1	0	0	INTER
A/116/CD1	A/301/C2	0	0	0	0	0	0	1	0	0	INTER
A/116/CD1	A/301/N1	0	0	0	0	0	0	1	0	0	INTER
A/116/CB	A/301/C6	1	0	0	0	0	0	0	0	0	INTER
A/116/CB	A/301/O6	1	0	0	0	0	0	0	0	1	INTER
A/301/C8	A/116/CE2	0	0	0	0	0	0	1	0	0	INTER
A/301/C8	A/116/CD2	0	0	0	0	0	0	1	0	0	INTER
A/301/N7	A/116/CD2	0	0	0	0	0	0	1	0	0	INTER
A/301/C5	A/116/CD2	0	0	0	0	0	0	1	0	0	INTER
A/301/N7	A/116/CG	0	0	0	0	0	0	1	0	0	INTER
A/301/C5	A/116/CG	1	0	0	0	0	0	1	0	0	INTER
A/301/C5	A/116/CD1	0	0	0	0	0	0	1	0	0	INTER
A/301/N9	A/116/CE2	0	0	0	0	0	0	1	0	0	INTER
A/301/N9	A/116/CZ	0	0	0	0	0	0	1	0	0	INTER

	A/301/N9	A/116/CD2	0	0	0	0	0	0	1	0	0	INTER
	A/301/C4	A/116/CE2	0	0	0	0	0	0	1	0	0	INTER
	A/301/C4	A/116/CZ	0	0	0	0	0	0	1	0	0	INTER
	A/301/C4	A/116/CD2	0	0	0	0	0	0	1	0	0	INTER
	A/301/C4	A/116/CE1	0	0	0	0	0	0	1	0	0	INTER
	A/301/C4	A/116/CG	0	0	0	0	0	0	1	0	0	INTER
	A/301/C4	A/116/CD1	0	0	0	0	0	0	1	0	0	INTER
	A/301/N7	A/112/CD	0	0	0	0	0	0	0	0	1	INTER
	A/301/N7	A/112/NZ	0	1	1	0	0	0	0	1	0	INTER
	A/154/CG	A/301/N2	0	1	0	0	0	0	0	0	0	INTER
	A/301/N2	A/151/O	1	0	1	0	0	0	0	1	0	INTER
	A/301/N2	A/155/CB	1	0	0	0	0	0	0	0	0	INTER
	A/301/O6	A/114/OG	1	0	0	0	0	0	0	1	0	INTER
	A/114/CB	A/301/O6	1	0	0	0	0	0	0	0	1	INTER
	A/154/OE2	A/301/C6	1	0	0	0	0	0	0	0	0	INTER
	A/154/OE2	A/301/O6	0	1	0	0	0	0	0	0	0	INTER
	A/154/OE2	A/301/N1	1	0	0	0	0	0	0	1	0	INTER
	A/154/CG	A/301/N1	0	0	0	0	0	0	0	0	1	INTER
6VCL	B/303/O3D	B/17/CG1	0	0	0	1	0	0	0	0	0	INTER
	B/303/O3D	B/17/N	0	0	0	0	0	0	0	1	0	INTER
	B/303/O3D	B/19/NZ	1	0	1	0	1	0	0	1	0	INTER
	B/303/O3C	B/19/NZ	1	0	1	0	0	0	0	1	0	INTER
	B/303/O3C	B/19/CE	1	0	0	0	0	0	0	0	1	INTER
	B/303/O2D	B/16/CA	1	0	0	0	0	0	0	0	1	INTER
	B/303/O2D	B/16/N	1	0	1	0	0	0	0	1	0	INTER
	B/301/MG	B/303/PC	1	0	0	0	0	0	0	0	0	INTER
	B/303/O2D	B/558/O	0	1	1	0	0	0	0	1	0	SELECTION_WATER
	B/301/MG	B/303/O2C	0	0	0	0	0	1	0	0	0	INTER
	B/302/K	B/303/C6	0	1	0	0	0	0	0	0	0	INTER
	B/302/K	B/303/O6	0	1	0	0	0	0	0	0	0	INTER
	B/303/O3G	B/558/O	1	0	1	0	0	0	0	1	0	SELECTION_WATER
	B/303/O3G	B/78/OH	0	1	1	0	0	0	0	1	0	INTER
	B/303/C5'	B/78/CE2	0	1	0	0	0	0	0	0	0	INTER

B/39/OG	B/303/O6	1	0	1	0	0	0	0	1	0	INTER
B/39/CB	B/303/O6	1	0	0	0	0	0	0	0	1	INTER
B/105/N	B/303/N3	0	1	1	0	0	0	0	1	0	INTER
B/104/CG2	B/303/C6	0	1	0	0	0	0	0	0	0	INTER
B/74/CD	B/303/N1	0	1	0	0	0	0	0	0	0	INTER
B/15/CA	B/303/O1C	0	1	0	1	0	0	0	0	1	INTER
B/406/O	B/303/O1C	1	0	1	0	0	0	0	1	0	SELECTION_WATER
B/303/O1C	B/19/NZ	1	0	1	0	0	0	0	1	0	INTER
B/303/PC	B/19/NZ	0	1	0	0	0	0	0	0	0	INTER
B/303/O1C	B/19/CE	0	0	0	0	0	0	0	0	1	INTER
B/303/N2	B/105/OE1	1	0	1	0	0	0	0	1	0	INTER
B/105/CG	B/303/N3	0	0	0	1	0	0	0	0	0	INTER
B/78/OH	B/303/O2C	1	0	0	0	0	0	0	1	0	INTER
B/558/O	B/303/O2C	1	0	1	0	0	0	0	1	0	SELECTION_WATER
B/74/OE1	B/303/N1	1	0	0	0	0	0	0	1	0	INTER
B/74/OE2	B/303/N1	0	0	0	0	0	0	0	1	0	INTER
B/303/N2	B/74/OE2	1	0	1	0	0	0	0	1	0	INTER
B/84/C	B/303/O6	0	1	0	0	0	0	0	0	0	INTER
B/84/CA	B/303/O6	0	0	0	0	0	0	0	0	1	INTER
B/84/N	B/303/O6	0	0	0	0	0	0	0	1	0	INTER
B/559/O	B/303/O3G	0	0	0	0	0	0	0	1	0	SELECTION_WATER
B/559/O	B/303/O2G	1	0	1	0	0	0	0	1	0	SELECTION_WATER
B/301/MG	B/303/O1B	1	0	0	0	1	1	0	0	0	INTER
B/301/MG	B/303/O3G	1	0	0	0	1	1	0	0	0	INTER
B/301/MG	B/303/O2G	0	0	0	0	1	0	0	0	0	INTER
B/301/MG	B/303/PG	1	0	0	0	0	0	0	0	0	INTER
B/560/O	B/303/O1B	1	0	1	0	0	0	0	1	0	SELECTION_WATER
B/301/MG	B/303/PB	1	0	0	0	0	0	0	0	0	INTER
B/506/O	B/303/O2B	0	1	1	0	0	0	0	1	0	SELECTION_WATER
B/560/O	B/303/O2B	1	0	1	0	0	0	0	1	0	SELECTION_WATER
B/560/O	B/303/PB	1	0	0	0	0	0	0	0	0	SELECTION_WATER
B/303/N9	B/83/CD2	0	0	0	0	0	0	1	0	0	INTER
B/302/K	B/303/C5	1	0	0	0	0	0	0	0	0	INTER

B/302/K	B/303/C8	1	0	0	0	0	0	0	0	0	INTER
B/302/K	B/303/N7	1	0	0	0	0	0	0	0	0	INTER
B/411/O	B/303/C8	0	0	0	0	0	0	0	0	1	SELECTION_WATER
B/19/CG	B/303/O3D	0	0	0	1	0	0	0	0	0	INTER
B/20/N	B/303/O1D	1	0	1	0	0	0	0	1	0	INTER
B/303/O3D	B/19/N	1	0	1	0	0	0	0	1	0	INTER
B/303/PD	B/560/O	1	0	0	0	0	0	0	0	0	SELECTION_WATER
B/303/O3C	B/560/O	0	0	1	0	0	0	0	1	0	SELECTION_WATER
B/303/O2D	B/525/O	1	0	1	0	0	0	0	1	0	SELECTION_WATER
B/301/MG	B/303/O1D	0	0	0	0	1	0	0	0	0	INTER
B/303/O3D	B/18/N	1	0	1	0	0	0	0	1	0	INTER
B/559/O	B/303/O2D	0	0	0	0	0	0	0	1	0	SELECTION_WATER
B/301/MG	B/303/PD	1	0	0	0	0	0	0	0	0	INTER
B/301/MG	B/303/O3C	0	1	0	0	0	0	0	0	0	INTER
B/301/MG	B/303/O2D	0	0	0	0	0	1	0	0	0	INTER
B/303/O1D	B/19/N	0	0	0	0	0	0	0	1	0	INTER
B/303/O1D	B/445/O	1	0	1	0	0	0	0	1	0	SELECTION_WATER
B/560/O	B/303/O1D	1	0	1	0	0	0	0	1	0	SELECTION_WATER
B/303/O2'	B/103/OE2	1	0	0	0	0	0	0	1	0	INTER
B/303/C2'	B/103/OE2	0	0	0	0	0	0	0	0	1	INTER
B/55/OH	B/303/O1A	1	0	0	0	0	0	0	1	0	INTER
B/303/O2A	B/43/NH2	0	0	0	0	1	0	0	0	0	INTER
B/303/O3A	B/46/NH1	0	0	1	0	0	0	0	1	0	INTER
B/303/O1G	B/46/NH1	0	0	0	0	1	0	0	0	0	INTER
B/303/O1G	B/46/NH2	0	0	1	0	1	0	0	0	0	INTER
B/303/O2A	B/46/NH1	0	0	0	0	1	0	0	0	0	INTER
B/303/O2A	B/43/CZ	0	0	0	0	1	0	0	0	0	INTER
B/303/O2A	B/43/NE	1	0	1	0	1	0	0	1	0	INTER
B/303/O2A	B/46/NH2	0	0	0	0	1	0	0	0	0	INTER
B/526/O	B/303/O3A	0	0	0	0	0	0	0	1	0	SELECTION_WATER
B/526/O	B/303/O1G	1	0	1	0	0	0	0	1	0	SELECTION_WATER
B/526/O	B/303/O2A	1	0	1	0	0	0	0	1	0	SELECTION_WATER
B/83/OH	B/303/O2A	1	0	1	0	0	0	0	1	0	INTER

	B/43/CZ	B/303/O1A	0	0	0	0	1	0	0	0	0	INTER
	B/43/NE	B/303/O1A	0	0	0	0	1	0	0	0	0	INTER
	B/303/O1A	B/561/O	1	0	1	0	0	0	0	1	0	SELECTION_WATER
	B/43/NH2	B/303/O1A	1	0	1	0	1	0	0	1	0	INTER
	B/303/C8	B/55/OH	0	0	0	1	0	0	0	0	1	INTER
	B/303/C8	B/55/CE1	0	0	0	0	0	0	1	0	0	INTER
	B/303/N7	B/55/CE1	0	0	0	1	0	0	1	0	0	INTER
	B/303/N7	B/39/OG	0	0	0	0	0	0	0	1	0	INTER
	B/83/CD2	B/303/C8	0	0	0	0	0	0	1	0	0	INTER
	B/83/CD2	B/303/N7	0	0	0	0	0	0	1	0	0	INTER
5U51	C/306/MG	A/301/PB	1	0	0	0	0	0	0	0	0	INTER
	C/306/MG	A/301/O2B	0	0	0	0	1	0	0	0	0	INTER
	A/64/NH2	A/301/O1B	0	0	0	0	1	0	0	0	0	INTER
	A/64/NH2	A/301/O2A	0	0	0	0	1	0	0	0	0	INTER
	C/100/ND2	A/301/O1B	0	0	1	0	0	0	0	1	0	INTER
	A/64/CZ	A/301/O1B	0	0	0	0	1	0	0	0	0	INTER
	A/64/CZ	A/301/O2A	1	0	0	0	1	0	0	0	0	INTER
	A/301/O4'	A/100/ND2	0	1	1	0	0	0	0	1	0	INTER
	A/301/C2	A/11/CD2	0	0	0	0	0	0	1	0	0	INTER
	A/301/C2	A/11/CZ	0	0	0	0	0	0	1	0	0	INTER
	A/301/N3	A/11/CZ	0	0	0	0	0	0	1	0	0	INTER
	A/301/N1	A/11/CD2	0	1	0	0	0	0	1	0	1	INTER
	A/301/N1	A/11/CZ	0	0	0	0	0	0	1	0	0	INTER
	A/301/N1	A/11/CG	0	0	0	0	0	0	1	0	0	INTER
	A/301/N1	A/11/CD1	0	0	0	0	0	0	1	0	0	INTER
	A/301/C4	A/11/CZ	0	0	0	0	0	0	1	0	0	INTER
	A/301/C6	A/11/CD2	0	0	0	0	0	0	1	0	0	INTER
	A/301/C6	A/11/CZ	0	0	0	0	0	0	1	0	0	INTER
	A/301/C6	A/11/CG	0	0	0	0	0	0	1	0	0	INTER
	A/301/C6	A/11/CE1	0	1	0	0	0	0	1	0	0	INTER
	A/301/C6	A/11/CD1	0	1	0	0	0	0	1	0	0	INTER
	A/301/C5	A/11/CZ	0	0	0	0	0	0	1	0	0	INTER
	A/301/C5	A/11/CE1	0	1	0	0	0	0	1	0	0	INTER

	A/301/C5	A/11/CD1	0	0	0	0	0	0	1	0	0	INTER
	A/301/O6	A/11/CD1	1	0	0	0	0	0	0	0	1	INTER
	C/306/MG	A/301/O3D	0	0	0	0	0	1	0	0	0	INTER
	A/301/O3B	C/100/OD1	1	0	0	0	0	0	0	0	0	INTER
	A/301/O3B	A/64/NH2	0	0	1	0	0	0	0	1	0	INTER
	C/306/MG	A/301/PD	0	1	0	0	0	0	0	0	0	INTER
	C/306/MG	A/301/O2C	1	0	0	0	1	1	0	0	0	INTER
	A/301/O3B	C/306/MG	0	0	0	0	0	1	0	0	0	INTER
	A/64/NH2	A/301/O2D	1	0	1	0	1	0	0	1	0	INTER
	A/64/NH2	A/301/O3D	0	1	1	0	0	0	0	1	0	INTER
	A/64/CZ	A/301/O2D	0	0	0	0	1	0	0	0	0	INTER
	C/100/OD1	A/301/O3D	1	0	0	0	0	0	0	0	0	INTER
	A/301/N1	A/52/O	1	0	0	0	0	0	0	1	0	INTER
	A/11/CE2	A/301/C6	0	0	0	0	0	0	1	0	0	INTER
	A/11/CE2	A/301/C2	0	0	0	0	0	0	1	0	0	INTER
	A/11/CE2	A/301/N3	0	0	0	0	0	0	1	0	0	INTER
	A/11/CE2	A/301/N1	0	0	0	0	0	0	1	0	1	INTER
	A/301/O6	A/53/CD	0	0	0	1	0	0	0	0	1	INTER
	A/100/ND2	A/301/O5'	1	0	1	0	0	0	0	1	0	INTER
	A/100/ND2	A/301/O1A	0	0	1	0	0	0	0	1	0	INTER
	A/101/NZ	A/301/O2B	0	0	1	0	1	0	0	1	0	INTER
	A/101/CE	A/301/O3A	1	0	0	0	0	0	0	0	1	INTER
	A/301/PA	A/64/NH1	0	1	0	0	0	0	0	0	0	INTER
	C/65/NZ	A/301/O1B	0	0	1	0	1	0	0	0	0	INTER
	C/65/NZ	A/301/O2A	0	0	0	0	1	0	0	0	0	INTER
	A/301/O1B	A/64/NH1	0	0	1	0	1	0	0	1	0	INTER
	A/301/O2A	A/64/NH1	1	0	1	0	1	0	0	1	0	INTER
	A/11/CE1	A/301/N7	0	0	0	0	0	0	1	0	0	INTER
	A/65/CB	A/301/N7	0	0	0	0	0	0	0	0	1	INTER
	A/65/N	A/301/N7	0	1	1	0	0	0	0	1	0	INTER
6G15	A/61/OD2	A/301/N1	0	0	0	0	0	0	0	1	0	INTER
	A/301/C2'	A/135/OG1	0	0	0	1	0	0	0	0	1	INTER
	A/301/O5'	A/135/OG1	0	0	0	0	0	0	0	1	0	INTER
	A/301/O5'	A/132/CA	0	0	0	1	0	0	0	0	0	INTER

A/301/O2A	A/134/OG	0	1	0	0	0	0	0	1	0	INTER
A/301/O1B	A/133/N	1	0	1	0	0	0	0	1	0	INTER
A/301/O3A	A/132/CA	0	1	0	0	0	0	0	0	1	INTER
A/301/O1B	A/133/CB	0	0	0	0	0	0	0	0	1	INTER
A/301/O1B	A/133/CG	0	0	0	0	0	0	0	0	1	INTER
A/414/O	A/301/O2B	0	1	1	0	0	0	0	1	0	SELECTION_WATER
A/301/O3G	A/434/O	0	0	0	0	0	0	0	1	0	SELECTION_WATER
A/301/O2G	A/434/O	1	0	1	0	0	0	0	1	0	SELECTION_WATER
A/301/O6	A/58/OD1	1	0	0	0	0	0	0	0	0	INTER
A/301/N1	A/59/CA	0	0	0	1	0	0	0	0	0	INTER
A/301/O6	A/59/CA	0	1	0	0	0	0	0	0	1	INTER
A/301/O6	A/59/N	0	1	1	0	0	0	0	1	0	INTER
A/301/N7	A/58/ND2	1	0	1	0	0	0	0	1	0	INTER
A/135/OG1	A/301/O1A	1	0	0	0	0	0	0	1	0	INTER
A/135/CB	A/301/O1A	0	0	0	0	0	0	0	0	1	INTER
A/88/N	A/301/O6	0	0	1	0	0	0	0	0	0	INTER
A/87/N	A/301/O6	1	0	1	0	0	0	0	1	0	INTER
A/86/CA	A/301/O6	0	1	0	1	0	0	0	0	1	INTER
A/86/OD1	A/301/N1	0	0	0	0	0	0	0	1	0	INTER
A/301/N1	A/61/OD1	1	0	0	0	0	0	0	1	0	INTER
A/301/O1A	A/132/CA	0	0	0	1	0	0	0	0	1	INTER
A/135/N	A/301/O1A	1	0	1	0	0	0	0	1	0	INTER
A/134/CB	A/301/O2B	0	0	0	0	0	0	0	0	1	INTER
A/134/N	A/301/O1A	0	0	0	0	0	0	0	1	0	INTER
A/134/N	A/301/O2B	1	0	1	0	0	0	0	1	0	INTER
A/134/OG	A/301/O1A	1	0	1	0	0	0	0	1	0	INTER
A/301/O2B	A/134/OG	1	0	0	0	0	0	0	1	0	INTER
A/434/O	A/301/O2B	1	0	1	0	0	0	0	1	0	SELECTION_WATER
A/301/C5'	A/130/O	0	0	0	1	0	0	0	0	0	INTER
A/301/O2A	A/414/O	0	1	1	0	0	0	0	1	0	SELECTION_WATER
A/301/N9	A/59/CE	0	0	0	1	0	0	0	0	0	INTER
A/301/O4'	A/59/CE	0	1	0	0	0	0	0	0	1	INTER
A/301/N2	A/61/OD2	1	0	1	0	0	0	0	1	0	INTER
A/301/O4'	A/59/NZ	0	1	1	0	0	0	0	1	0	INTER
A/301/O1D	A/88/NZ	1	0	1	0	1	0	0	1	0	INTER
A/133/NZ	A/301/O1B	1	0	1	0	1	0	0	1	0	INTER
A/301/O3B	A/130/CA	0	0	0	0	0	0	0	0	1	INTER
A/301/O3B	A/130/N	1	0	1	0	0	0	0	1	0	INTER
A/301/O1G	A/130/N	0	0	1	0	0	0	0	1	0	INTER
A/301/O3A	A/130/CA	0	0	0	1	0	0	0	0	0	INTER
A/301/O1B	A/130/N	0	0	0	0	0	0	0	1	0	INTER
A/301/O1B	A/132/N	0	1	1	0	0	0	0	1	0	INTER

	A/301/O3A	A/132/N	1	0	1	0	0	0	0	1	0	INTER
	A/131/N	A/301/O1B	0	0	1	0	0	0	0	1	0	INTER
	A/301/O2G	A/129/CA	0	0	0	1	0	0	0	0	0	INTER
	A/301/O1G	A/129/CB	0	1	0	1	0	0	0	0	1	INTER
	A/301/O3G	A/414/O	1	0	1	0	0	0	0	1	0	SELECTION_WATER
	A/133/NZ	A/301/O2G	1	0	1	0	1	0	0	1	0	INTER
6EX0	A/301/N7	A/147/NZ	0	0	1	0	0	0	0	1	0	INTER
	A/301/C4	A/151/CE2	0	0	0	0	0	0	1	0	0	INTER
	A/301/N7	A/151/CE2	0	0	0	0	0	0	1	0	0	INTER
	A/301/N7	A/147/CE	0	0	0	0	0	0	0	0	1	INTER
	A/301/N9	A/151/CE2	0	0	0	0	0	0	1	0	0	INTER
	A/112/NH2	A/301/O2'	0	1	1	0	0	0	0	1	0	INTER
	A/151/CZ	A/301/C8	0	0	0	0	0	0	1	0	0	INTER
	A/112/CZ	A/301/O1C	0	0	0	0	1	0	0	0	0	INTER
	A/112/NH2	A/301/O1C	1	0	1	0	1	0	0	1	0	INTER
	A/151/CD1	A/301/N1	0	0	0	0	0	0	1	0	0	INTER
	A/151/CD1	A/301/C6	0	1	0	0	0	0	1	0	0	INTER
	A/151/CD1	A/301/C5	0	0	0	0	0	0	1	0	0	INTER
	A/151/CD1	A/301/C4	0	0	0	0	0	0	1	0	0	INTER
	A/151/CG	A/301/C6	0	0	0	0	0	0	1	0	0	INTER
	A/151/CG	A/301/C5	0	0	0	0	0	0	1	0	0	INTER
	A/151/CE1	A/301/C5	0	0	0	0	0	0	1	0	0	INTER
	A/151/CE1	A/301/C4	1	0	0	0	0	0	1	0	0	INTER
	A/151/CE1	A/301/N9	0	0	0	0	0	0	1	0	0	INTER
	A/151/CD2	A/301/C5	0	0	0	0	0	0	1	0	0	INTER
	A/151/CD2	A/301/N7	0	0	0	0	0	0	1	0	0	INTER
	A/151/CZ	A/301/C4	0	1	0	0	0	0	1	0	0	INTER
	A/151/CZ	A/301/N9	0	0	0	0	0	0	1	0	0	INTER
	A/301/O2D	A/193/NH1	0	0	1	0	1	0	0	1	0	INTER
	A/301/N1	A/189/OE1	1	0	0	0	0	0	0	1	0	INTER
	A/301/O6	A/149/ND2	1	0	1	0	0	0	0	1	0	INTER
	A/193/NH2	A/301/O6	0	0	1	0	0	0	0	1	0	INTER
	A/193/NH2	A/301/C6	1	0	0	0	0	0	0	0	0	INTER
	A/301/C5	A/193/NH2	1	0	0	0	0	0	0	0	0	INTER
	A/301/N2	A/186/O	1	0	1	0	0	0	0	1	0	INTER
	A/301/N2	A/186/C	1	0	0	0	0	0	0	0	0	INTER
	A/301/N2	A/186/CA	0	1	0	0	0	0	0	0	0	INTER
	A/151/CD1	A/301/C2	0	0	0	0	0	0	1	0	0	INTER
	A/151/CE1	A/301/C2	0	0	0	0	0	0	1	0	0	INTER
	A/301/N3	A/151/CD1	0	0	0	0	0	0	1	0	0	INTER
	A/151/CE1	A/301/N3	1	0	0	0	0	0	1	0	1	INTER
	A/151/CZ	A/301/N3	0	0	0	0	0	0	1	0	0	INTER

	A/78/NH2	A/301/O2C	0	0	0	0	1	0	0	0	0	INTER
	A/401/O	A/301/O3G	1	0	1	0	0	0	0	1	0	SELECTION_WATER
	A/301/O2A	A/147/NZ	0	1	1	0	1	0	0	1	0	INTER
	A/176/NE2	A/301/O2'	1	0	1	0	0	0	0	1	0	INTER
	A/176/NE2	A/301/O3'	0	0	1	0	0	0	0	0	0	INTER
	A/155/CE1	A/301/O4'	1	0	0	0	0	0	0	0	0	INTER
	A/301/C8	A/151/CE2	0	0	0	0	0	0	1	0	0	INTER
	A/301/C1'	A/151/OH	0	0	0	1	0	0	0	0	1	INTER
6S2T	A/401/N7	A/44/CB	0	0	0	1	0	0	0	0	1	INTER
	A/401/N7	A/44/N	1	0	1	0	0	0	0	1	0	INTER
	A/401/PC	A/404/MN	0	1	0	0	0	0	0	0	0	INTER
	A/401/O1C	A/404/MN	0	0	0	0	0	1	0	0	0	INTER
	A/49/OH	A/401/O2'	1	0	1	0	0	0	0	1	0	INTER
	A/402/O2	A/401/C1'	0	0	0	1	0	0	0	0	0	INTER
	A/150/O	A/401/N2	1	0	1	0	0	0	0	1	0	INTER
	A/401/O2A	A/161/CD	0	0	0	1	0	0	0	0	1	INTER
	A/401/O2A	A/161/NZ	1	0	1	0	1	0	0	1	0	INTER
	A/401/O2A	A/161/CE	0	0	0	0	0	0	0	0	1	INTER
	A/150/OD1	A/401/C3'	0	0	0	1	0	0	0	0	0	INTER
	A/401/O2D	A/405/MG	0	0	0	0	1	0	0	0	0	INTER
	A/147/NH1	A/401/O3D	0	0	0	0	1	0	0	0	0	INTER
	A/401/O3C	A/150/OD1	0	1	0	0	0	0	0	0	0	INTER
	A/405/MG	A/401/O3C	1	0	0	0	0	0	0	0	0	INTER
	A/401/PD	A/405/MG	1	0	0	0	0	0	0	0	0	INTER
	A/401/O3D	A/143/NZ	1	0	1	0	1	0	0	1	0	INTER
	A/401/O3D	A/405/MG	1	0	0	0	1	0	0	0	0	INTER
	A/147/NH1	A/401/O2D	0	0	1	0	1	0	0	1	0	INTER
	A/164/NH2	A/401/O1A	0	0	1	0	1	0	0	1	0	INTER
