# Supplementary material for: Structural Analysis of (p)ppGpp Reveals Its Versatile Binding Pattern for Diverse Types of Target Proteins
Source: Front Microbiol. 2020 Nov 5;11:575041. doi: 10.3389/fmicb.2020.575041 (PMC7674647; doi:10.3389/fmicb.2020.575041)
Supplement: Supplementary file 2 [file Data_Sheet_2.PDF]

Structural analysis of (p)ppGpp reveals its versatile binding pattern for diverse types of target proteins

Gajraj Singh Kushwaha[1*] Anupam Patra[1], and Neel Sarovar Bhavesh[1*]
[1]Transcription Regulation Group, International Centre for Genetic Engineering and Biotechnology (ICGEB), ArunaAsaf Ali Marg, New Delhi 110067, India.

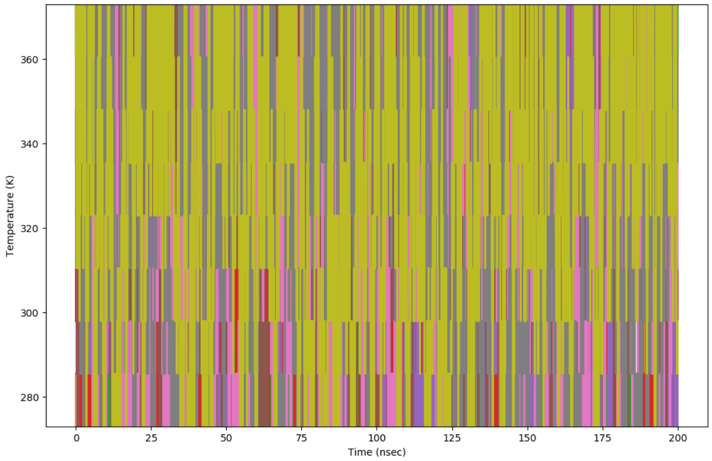

Table-1S: Replica exchange molecular dynamic simulation analysis for ppGpp

Replica pairs (K)	Total exchanges	No of accepts	No of rejects	Accept ratio
273.00 – 285.50	105575	2010	103565	0.019
285.50 – 298.00	61091	1552	59539	0.025
298.00 – 310.50	74901	2363	72538	0.032
310.50 – 323.00	71485	3037	68448	0.042
323.00 – 335.50	71379	3640	67739	0.051
335.50 – 348.00	74977	4595	70382	0.061
348.00 – 360.50	61037	4367	56670	0.072
360.50 – 373.00	105629	8776	96853	0.083

(A)

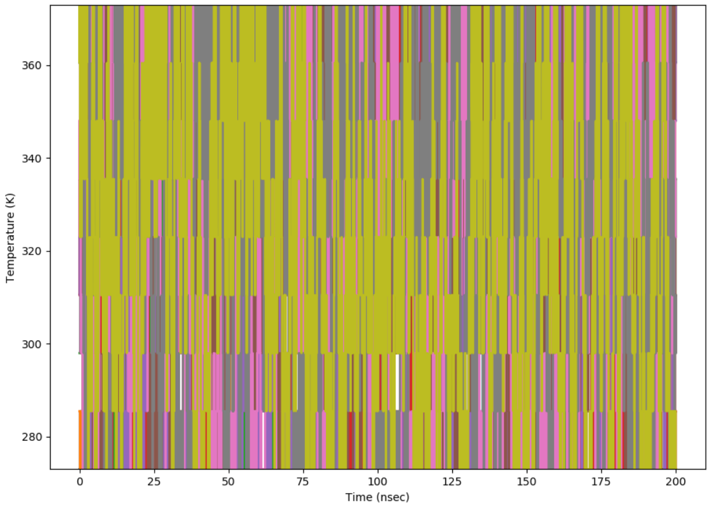

Table-2S: Replica exchange molecular dynamic simulation analysis for pppGpp

Replica pairs (K)	Total exchanges	No of accepts	No of rejects	Accept ratio
273.00 – 285.50	105575	1421	104154	0.013
285.50 – 298.00	61091	1217	59874	0.020
298.00 – 310.50	74901	1966	72935	0.026
310.50 – 323.00	71485	2414	69071	0.034
323.00 – 335.50	71379	2991	68388	0.042
335.50 – 348.00	74977	3670	71307	0.049
348.00 – 360.50	61037	3506	57531	0.057
360.50 – 373.00	105629	7347	98282	0.070

(B)

Figure S1- Replica exchange molecular dynamics simulation of (A) ppGpp, and (B) pppGpp shows the exchange of replicas at various temperatures.All eight replicas are colored uniquely to show its temperature as a function of time. The adjacent tables (Table 1S for ppGpp and Table 2S for pppGpp) show the values replica exchanges at different temperature steps.

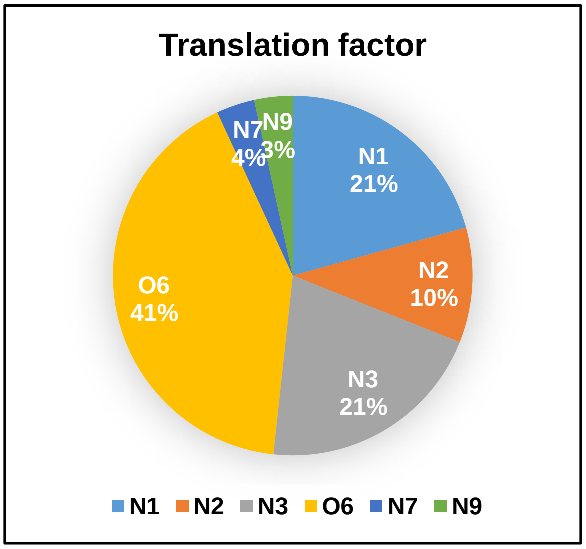

Figure S2A

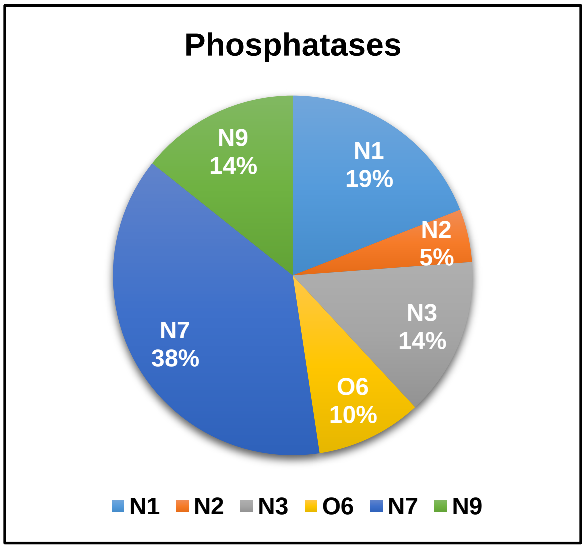

Figure S2B

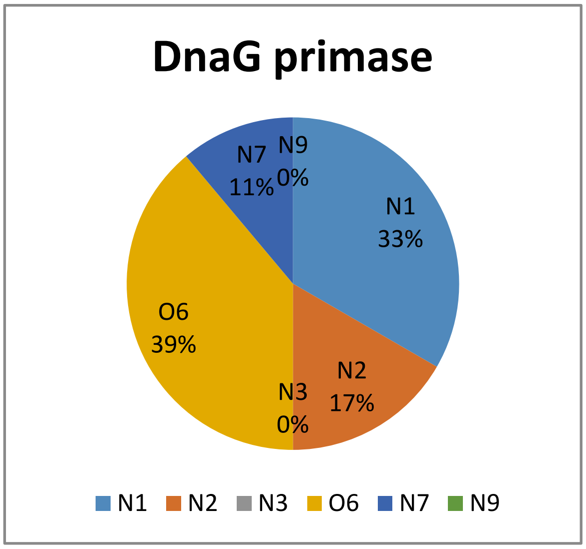

Figure S2C

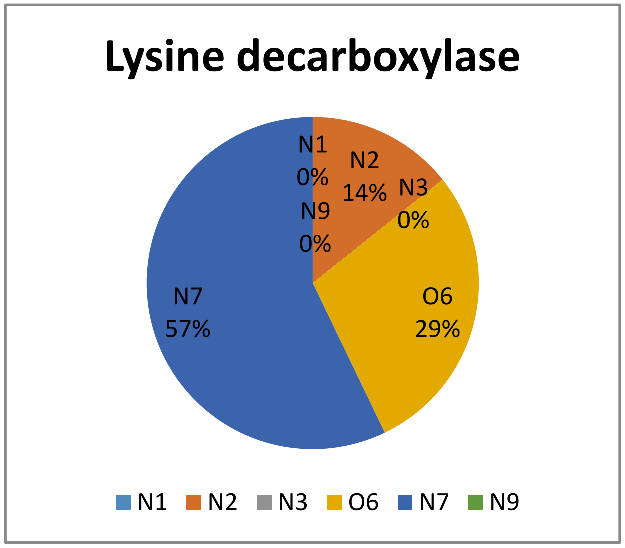

Figure S2D

Figure-S2: Occurrence ofinteractions between (p)ppGpp atoms and protein are identified in the crystal structures of (p)ppGpp-protein complexes.
